# New 6,19-oxidoandrostan derivatives obtained by biotransformation in environmental filamentous fungi cultures

**DOI:** 10.1186/s12934-020-01303-6

**Published:** 2020-02-17

**Authors:** Ewa Kozłowska, Agata Matera, Jordan Sycz, Anna Kancelista, Edyta Kostrzewa-Susłow, Tomasz Janeczko

**Affiliations:** 1 Department of Chemistry, Wrocław University of Environmental and Life Sciences, Norwida 25, 50-375 Wrocław, Poland; 2 Department of Biotechnology and Food Microbiology, Wrocław University of Environmental and Life Sciences, Chełmońskiego 37, 51-630 Wrocław, Poland

**Keywords:** Biotransformation, Steroids, Baeyer–Villiger oxidation, Oxidoandrostan-17-one, Oxirane bridge, *Beauveria bassiana*

## Abstract

**Background:**

Steroid compounds with a 6,19-oxirane bridge possess interesting biological activities including anticonvulsant and analgesic properties, bacteriostatic activity against Gram-positive bacteria and selective anti-glucocorticoid action, while lacking mineralocorticoid and progestagen activity.

**Results:**

The study aimed to obtain new derivatives of 3β-acetyloxy-5α-chloro-6,19-oxidoandrostan-17-one by microbial transformation. Twelve filamentous fungal strains were used as catalysts, including entomopathogenic strains with specific activity in the transformation of steroid compounds. All selected strains were characterised by high biotransformation capacity for steroid compounds. However, high substrate conversions were obtained in the cultures of 8 strains: *Beauveria bassiana* KCh BBT, *Beauveria caledonica* KCh J3.4, *Penicillium commune* KCh W7, *Penicillium chrysogenum* KCh S4, *Mucor hiemalis* KCh W2, *Fusarium acuminatum* KCh S1, *Trichoderma atroviride* KCh TRW and *Isaria farinosa* KCh KW1.1. Based on gas chromatography (GC) and nuclear magnetic resonance (NMR) analyses, it was found that almost all strains hydrolysed the ester bond of the acetyl group. The strain *M. hiemalis* KCh W2 reduced the carbonyl group additionally. From the *P. commune* KCh W7 and *P. chrysogenum* KCh S4 strain cultures a product of D-ring Baeyer–Villiger oxidation was isolated, whereas from the culture of *B. bassiana* KCh BBT a product of hydroxylation at the 11α position and oxidation of the D ring was obtained. Three 11α-hydroxy derivatives were obtained in the culture of *I. farinosa* KCh KW1.1: 3β,11α-dihydroxy-5α-chloro-6,19-oxidoandrostan-17-one, 3β,11α,19-trihydroxy-5α-chloro-6,19-oxidoandrostan-17-one and 3β,11α-dihydroxy-5α-chloro-6,19-oxidoandrostan-17,19-dione. They are a result of consecutive reactions of hydrolysis of the acetyl group at C-3, 11α- hydroxylation, then hydroxylation at C-19 and its further oxidation to lactone.

**Conclusions:**

As a result of the biotransformations, seven steroid derivatives, not previously described in the literature, were obtained: 3β-hydroxy-5α-chloro-6,19-oxidoandrostan-17-one, 3β,17α-dihydroxy-5α-chloro-6,19-oxidoandrostane, 3β-hydroxy-5α-chloro-17α-oxa-D-homo-6,19-oxidoandrostan-17-one, 3β,11α-dihydroxy-5α-chloro-17α-oxa-D-homo-6,19-oxidoandrostan-17-one and the three above–mentioned 11α-hydroxy derivatives. This study will allow a better understanding and characterisation of the catalytic abilities of individual microorganisms, which is crucial for more accurate planning of experiments and achieving more predictable results.

## Background

Steroid drugs are the second largest group of medicines that are used to treat and prevent various diseases. There is a close relationship between the structure of the steroid compound and its biological activity, and it is important to develop efficient methods for producing active pharmaceutical ingredients, key intermediates and new derivatives [1–4]. Biotransformations are a good alternative to chemical synthesis in obtaining steroid derivatives, with regio- and stereoselectively introduced substituents in nonactivated positions [5, 6]. Biotransformations can replace a multi-step synthesis with a single microbial transformation [7, 8].

The 6,19-oxirane bridge in the structure of steroid compounds causes the skeleton to flex between the A and B rings [9]. It has been shown that some compounds having such a moiety have interesting biological activities. The 21-hydroxyprogesterone analogue is a selective anti-glucocorticoid lacking mineralocorticoid and progestagen activity [10], and the pregnenolone analogue is a potent anticonvulsant [11, 12]. There are also reports of preventing ventricular fibrillation of 6,19-oxido androstanes [13]. They have analgesic properties and are bacteriostatic against Gram-positive bacteria [13]. Steroid compounds having a 6,19-oxirane bridge are precursors of the contraceptive 19-norsteroids [14].

In this study, biotransformations were carried out in cultures of filamentous fungi, including entomopathogenic fungi. All strains used belong to the collection of the Department of Chemistry of Wrocław University of Environmental and Life Sciences, and all but one (the strain *Beauveria bassiana* KCh BBT obtained Tenerife) come from the Lower Silesia district. These strains are: *Beauveria bassiana* KCh J1, *Beauveria bassiana* KCh BBT, *Isaria fumosorosea* KCh J2, *Isaria farinosa* KCh KW1.1, *Beauveria caledonica* KCh J3.3 and KCh J3.4, *Aspergillus niger* KCh M1, *Penicillium commune* KCh W7, *Penicillium chrysogenum* KCh S4, *Mucor hiemalis* KCh W2, *Fusarium acuminatum* KCh S1 and *Trichoderma atroviride* KCh TRW.

*Beauveria* is an amorphous, cosmopolitan type of filamentous fungus that belongs to the Ascomycota. The natural habitat of these organisms is soil. Species belonging to the genus *Beauveria*, including *B. bassiana*, have entomopathogenic properties and are used as insecticides. The mentioned species is a parasite of such orders of insects as Lepidoptera, Hemiptera, Coleoptera, Hymenoptera, Homoptera, Hemiptera and Orthoptera [15]. The teleomorphic form of *B. bassiana* is *Cordyceps bassiana* [16].

The possibility of using *B. bassiana* against a wide spectrum of insects has made it the object of numerous studies, and over time also its catalytic properties have been investigated. Many studies have shown that the *B. bassiana* enzyme apparatus has the ability to transform a variety of substrates including aromatic amines [17], amino acids [18], terpenes [19], flavonoids [20] and steroids [21–23] and is able to carry out, among other reactions, hydroxylation [21, 22], acetylation [17], epoxidation [24], Baeyer–Villiger oxidation [22, 23], glycosylation [17, 25], sulfoxidation [18], dealkylation, reduction and ester hydrolysis [26]. Among the biotransformations of steroid compounds carried out in *B. bassiana* cultures, the following transformations have been described: hydroxylation at the 11α [23, 26] or 7α position [21] and Baeyer–Villiger D-ring oxidation [22, 23]. *B. bassiana* KCh BBT and *B. bassiana* KCh J1 strains were used for biotransformation of DHEA (dehydroepiandrosterone). The *B. bassiana* KCh J1 strain was capable of stereoselective hydroxylation of DHEA at the 7α and 11α positions and oxidation of the hydroxyl group at C-7. This strain also showed a high rate of conversion – after 24 h, 70% conversion of DHEA to 7α-hydroxy-DHEA was observed, and after 72 h degradation of all biotransformation products began. *B. bassiana* KCh BBT also carried out hydroxylation reactions at the 7α and 11α positions. Baeyer–Villiger oxidation of the D-ring products has also been observed [27].

*Beauveria caledonica*, like *B. bassiana*, is classified as an entomopathogenic fungus – it parasitises beetles of the species *Hylurgus ligniperda* and *Hylastes ater*, which are pine pests [28]. *B. caledonica* has high resistance to heavy metals such as cadmium, copper, lead and zinc, which is associated with the ability to excrete a large amount of oxalic acid—a chelating agent [29]. This strain’s ability to dissolve minerals and reconstruct them is also described [30]. In the field of biotransformation of organic compounds, *B. caledonica* has shown the ability to oxidise sulphur amino acids [18, 31]. In our previous studies, *Beauveria caledonica* KCh J3.3 and KCh J3.4 were used for biotransformation of DHEA, resulting in effective hydroxylation of the substrate at the 7α, 7β and 11α positions, as well as oxidation of the C-7 hydroxyl group [27]. *B. caledonica* KCh J3.4 additionally showed the ability to hydrogenate the double bond between C-5 and C-6 and oxidised the hydroxyl group located at the carbon C-3.

The species *Isaria fumosorosea*, like the biocatalysts discussed above, belongs to cosmopolitan entomopathogenic fungi. In many countries, it is used as an insecticide—it parasitises insects such as aphids and whiteflies [32]. Promising results were also obtained using this species as a biocatalyst. It has been proven that strains from this species can perform effective glycosylation of flavonoid compounds [33–37]. *I. fumosorosea* KCh J2 was also used to transform steroid compounds such as DHEA, androstenedione, adrenosterone, 17α-methyltestosterone and estrone. As a result of these reactions, hydroxylation products at the 6β, 7α, 7β, 12β and 15β positions were isolated. Additionally, in the case of DHEA, Baeyer–Villiger oxidation of the D ring and oxidation of the hydroxyl group at C-7 were observed [38, 39]. Both estrone and estradiol underwent cascade transformations leading to the formation of many derivatives having in their structure a lactone group, a hydroxyl group and a sugar unit [40].

The high virulence of *Isaria farinosa*, like other mentioned entomopathogenic strains, is closely connected with high chitinase, lipase and protease activities [41]. During the biotransformation of several steroid compounds in the culture of twelve strains from this species, its high ability to hydroxylate DHEA leading to 7α- and 7β-hydroxy derivatives was found. During incubation of progesterone in the culture of the *Isaria farinosa* KCh KW1.1 strain, 6β,11α-dihydroxyprogesterone was obtained with high conversion [42].

Belonging to the order Ascomycota, *Aspergillus niger* is a common filamentous fungus. In biotechnology, it is used primarily for the production of citric acid and enzymes (including glucose oxidase, glucoamylase, pectinesterase, chitinase), which are used in the food, pharmaceutical and biocatalysis industries [43]. Enantioselective epoxide hydrolase was also isolated from *A. niger* [44]. The species was used for biocatalysis of terpenoids [45], furanocoumarins [46], flavonoids [47–50] and saponin [51]. The strains of *A. niger* have also been used for biotransformation of steroid compounds. They can carry out reactions such as hydroxylation at the 6β, 7α, 7β, 11β, 15β, 16α, 16β positions, ester bond hydrolysis, oxidation, double bond isomerisation [52, 53], epoxidation, chlorine atom substitution [54] and dehydrogenation [55].

*Penicillium commune* is known to be responsible, among other things, for food spoilage. It produces aflatoxins such as cyclopiazonic acid [56]. This species was used as a biocatalyst in the synthesis of chiral hydroxyphosphonates [57] and for DHEA biotransformation, in which it performed D-ring Baeyer–Villiger oxidation and double-bond isomerisation [58].

The species *Penicillium chrysogenum* is known primarily for the production of antibiotics, including penicillin. It is common in the temperate and subtropical climate zones. Lipases of this species are used to acylate primary and secondary alcohols [59]. *P. chrysogenum* is also used for the production of enzymes, including glucose oxidase [60]. Some strains have also been used for biotransformation of steroid compounds. *P. chrysogenum* performed hydrogenation of the double bond in testosterone [61] and Baeyer–Villiger oxidation of the D ring of DHEA [58].

Mucorales are cosmopolitan soil fungi. Most of the species included in this order cause food spoilage and several of them are pathogenic to mammals and plants [62]. *Mucor hiemalis* is the most common representative of the Mucorales order and has found several applications in the food and biotechnology industries. According to the results of their research, Heidary et al. achieved comparable yields in the production of ethanol to *S. cerevisiae* [62]. *M. hiemalis* has been tested for the ability to transform steroid compounds, including testosterone and progesterone. It has been shown that it carries out hydroxylation at the 14α position, and with prolonged incubation introduces hydroxyl groups also at the 6β and 7α positions [63]. Another study also managed to isolate the oxidation products of the hydroxyl group at the C-17 of testosterone [8]. As a result of DHEA biotransformation in the culture of the *M. hiemalis* KCh W2 strain, hydroxylation products at the 7α position and reduction of the carbonyl group at C-17 were obtained [58].

The genus *Fusarium* is one of the most important plant pathogens. It causes root rot and is especially dangerous for maize, soybean and wheat crops. *F. acuminatum* can produce mycotoxins [64]. This species was used as a biocatalyst in the transformation of DHEA, as a result of which its 7α-hydroxy derivative was obtained with high efficiency [58]. *Trichoderma atroviride* is a cosmopolitan filamentous fungus commonly found in soil. It is used for biological control of phytopathogens [65]. Research is also being conducted into the antibacterial and antioxidant properties of metabolites of this species [66]. To date, *T. atroviride* has not been used as a biocatalyst in the transformation of steroid compounds.

Given the current observations of the versatility of microorganisms in biotransformation, it is justified to study new species and relevant groups of chemical compounds in this regard. This study will allow a better understanding and characterisation of the catalytic abilities of individual microorganisms, which is crucial for more accurate planning of experiments and achieving more predictable results.

## Materials and methods

### Materials

The substrate, 3β-acetyloxy-5α-chloro-6,19-oxidoandrostan-17-one (**1**), was obtained from the resources of the Department of Chemistry, Wrocław University of Environmental and Life Sciences, Poland.

Microorganisms: The entomopathogenic strains—two strains of *Beauveria caledonica* (KCh J3.3 and KCh J3.4), two strains of *Beauveria bassiana* (KCh BBT, KCh J1), *Isaria fumosorosea* KCh J2 and *Isaria farinosa* KCh KW1.1—were isolated in the Lower Silesia district and on Tenerife (*Beauveria bassiana* KCh BBT). Their isolation and genetic determination was described previously [27, 33, 42]. The method of isolation and determination of *Penicillium commune* KCh W7, *Penicillium chrysogenum* KCh S4, *Mucor hiemalis* KCh W2, *Fusarium acuminatum* KCh S1 and *Trichoderma atroviride* KCh TRW was also described [58, 67]. Due to the unsatisfactory reaction, the strain *Aspergillus niger* KCh M1 was determined only on the basis of morphological features. All strains used in this study are from the collection of the Department of Chemistry of the Wrocław University of Environmental and Life Sciences.

### Screening procedure

One hundred millilitres of the sterile cultivation medium (3 g of glucose and 1 g of aminobac dissolved in water) in Erlenmeyer flasks (300 mL) was inoculated with a suspension of microorganisms and then incubated for 3 days at 25 °C on a rotary shaker (150 rpm). Then 10 mg of 3β-acetyloxy-5α-chloro-6,19-oxidoandrostan-17-one **(1)** dissolved in 1 mL of DMSO was added. After 1, 3, 7 and 10 days of incubation under the above conditions, portions of 10 mL of the transformation mixture were taken out and extracted with chloroform. The extracts were dried over anhydrous MgSO_4_, concentrated *in vacuo* and analysed by gas chromatography (GC) and thin-layer chromatography (TLC). All the experiments were repeated three times.

### Preparative biotransformation

Selected transformations were performed on the preparative scale in 2000 mL flasks, each containing 500 mL of the cultivation medium. After 3-day incubation (conditions as above) 100 mg of substrate **1** dissolved in 2 mL of DMSO was added. After the time specified for each transformation, the medium was extracted with CHCl_3_ (3 × 300 mL), dried (anhydrous MgSO_4_) and concentrated *in vacuo*. The transformation products were separated by preparative TLC and analysed (TLC, GC, NMR).

### Analytical methods

The course of biotransformation was monitored using TLC. The composition of product mixtures was established by GC. Products were separated using preparative TLC plates (Silica Gel GF, 20 × 20 cm, 500 μm, Analtech) and a hexane/acetone mixture (2:1, v/v) as an eluent. Analytical TLC was carried out on silica gel G (Merck). Compounds were detected by spraying the plates with an H_2_SO_4_/CH_3_OH mixture (1:1, v/v). GC analysis was performed using a Hewlett-Packard 5890A (Series II) GC instrument fitted with a flame ionisation detector (FID). An HP-5 (crosslinked phenyl methyl siloxane) capillary column (30 m × 0.32 mm × 0.25 μm) was used to determine the composition of product mixtures. The following temperature programme was used: 220 °C (1 min)/4 °C/min/260 °C (1 min)/30 °C/min/300 °C (5 min). For gas chromatography–mass spectrometry GC–MS analysis, a GCMS-SATURN 2000 instrument (Varian, nowadays Agilent, Santa Clara, CA, USA) was used with a ZB-1 (crosslinked phenyl-methylsiloxane) capillary column (30 m × 0.25 mm × 0.25 μm). The following temperature programme was used: 250 °C (1 min)/5 °C/min/300 °C (6 min) (Additional file 1). The NMR spectra were recorded on a DRX 500 MHz Bruker spectrometer and measured in CDCl_3_ and DMSO-*d*_6_. The products’ structures were determined by means of elemental analysis, ^1^H-NMR, ^13^C-NMR and correlation spectroscopy (HMBC, HMQC and COSY).

## Results and discussion

### Spectral data and isolated yields of products

#### Substrate—3β-acetyloxy-5α-chloro-6,19-oxidoandrostan-17-one (**1**)

^1^H NMR (600 MHz) (ppm) (CDCl_3_) δ: 0.90 (s, 3H, 18-H); 1.16 (qd, 1H, *J* = 12.9, 3.9 Hz, 11-Hβ); 1.33 (td, 1H, *J* = 13.1, 4.1 Hz, 7-Hα); 1.47–1.58 (m, 5H, 1-Hα, 2-Hα, 11-Hα, 14-H, 15-Hβ); 1.63–1.71 (m, 2H, 1-Hβ, 9-H); 1.72–1.80 (m, 2H, 8-H, 12-Hα); 1.83 (dt, 1H, *J* = 12.9, 3.1 Hz, 7-Hβ); 1.86–1.90 (m, 1H, 15-Hα); 1.93 (dd, 1H, *J* = 12.9, 11.1 Hz, 12-Hβ); 1.98–2.03 (m, 1H, 2-Hβ); 2.03 (s, 3H, –COC*H*_3_); 2.07 (dt, 1H, *J* = 19.3, 8.9 Hz, 16-Hα); 2.14 (dd, 1H, *J* = 13.6, 11.6 Hz, 4-Hα); 2.22 (ddd, 1H, *J* = 13.6, 4.5, 2.2 Hz, 4-Hβ); 2.43 (dd, 1H, *J* = 19.2, 8.6 Hz, 16-Hβ); 3.82 (d, 1H, *J* = 8.6 Hz, one of 19-H); 3.95 (d, 1H, *J* = 8.6 Hz, one of 19-H); 4.04 (d, 1H, *J* = 4.6 Hz, 6-Hα); 5.10 (tt, 1H, *J* = 11.4, 4.6 Hz, 3-Hα).

^1^H NMR (600 MHz) (ppm) (DMSO-*d*_6_) δ: 0.81 (s, 3H, 18-H); 1.14–1.20 (m, 2H, 7-Hα, 11-Hβ); 1.32 (td, 1H, *J* = 13.8, 4.7 Hz, 1-Hα); 1.35–1.43 (m, 2H, 11-Hα, 14-H); 1.48 (tt, 1H, *J* = 12.6, 9.0 Hz, 15-Hβ); 1.51–1.57 (m, 2H, 2-Hα, 9-H); 1.57–1.65 (m, 3H, 1-Hβ, 7-Hβ, 12-Hα); 1.68 (td, 1H, *J* = 10.7, 5.4 Hz, 8-H); 1.73–1.83 (m, 3H, 2-Hβ, 12-Hβ, 15-Hα); 1.96 (s, 3H, –COC*H*_3_); 2.00 (dd, 1H *J* = 19.3, 8.7 Hz, 16-Hα); 2.01 (t, 1H, *J* = 12.6 Hz, 4-Hα); 2.13 (ddd, 1H, *J* = 13.5, 4.2, 2.1 Hz, 4-Hβ); 2.33 (dd, 1H, *J* = 19.2, 8.2 Hz, 16-Hβ); 3.79 (d, 1H, *J* = 8.9 Hz, one of 19-H); 3.86 (d, 1H, *J* = 8.8 Hz, one of 19-H); 4.01 (d, 1H, *J* = 4.6 Hz, 6-Hα); 4.92 (tt, 1H, *J* = 11.5, 4.5 Hz, 3-Hα);

#### 3β-hydroxy-5α-chloro-6,19-oxidoandrostan-17-one (**2**)

After 3 days’ transformation of 100 mg of (**1**) in the *Mucor hiemalis* KCh W2 culture the isolation yield of (**2**) was 16 mg.

^1^H NMR (600 MHz) (ppm) (CDCl_3_) δ: 0.91 (s, 3H, 18-H); 1.18 (qd, 1H, *J* = 12.8, 3.8 Hz, 11-Hβ); 1.33 (td, 1H, *J* = 13.0, 4.0 Hz, 7-Hα); 1.40–1.57 (m, 5H, 1-Hα, 2-Hα, 11-Hα, 14-H, 15-Hβ); 1.62–1.70 (m, 2H, 1-Hβ, 9-H); 1.71–1.78 (m, 2H, 8-H, 12-Hα); 1.78–1.83 (m, 1H, 7-Hβ);

1.83–1.90 (m, 1H, 15-Hα); 1.90–1.98 (m, 2H, 2-Hβ, 12-Hβ); 2.05 (dd, 1H, *J* = 13.6, 11.6 Hz, 4-Hα); 2.08 (dt, 1H, *J* = 19.3, 8.9 Hz, 16-Hα); 2.20 (ddd, 1H, *J* = 13.8, 4.4, 2.3 Hz, 4-Hβ); 2.44 (dd, 1H, *J* = 19.1, 7.8 Hz, 16-Hβ); 3.81 (d, 1H, *J* = 8.6 Hz, one of 19-H); 3.95 (d, 1H, *J* = 8.5 Hz, one of 19-H); 4.05 (d, 1H, *J* = 4.4 Hz, 6-Hα); 4.07 (tt, 1H, *J* = 11.4, 4.6 Hz, 3-Hα).

#### 3β,17α-dihydroxy-5α-chloro-6,19-oxidoandrostan (**3**)

After 3 days’ transformation of 100 mg of (**1**) in the *Mucor hiemalis* KCh W2 culture the isolation yield of (**3**) was 38 mg.

^1^H NMR (600 MHz) (ppm) (CDCl_3_) δ: 0.70 (s, 3H, 18-H); 1.20 (dt, 1H, *J* = 12.9, 3.9 Hz, 11-Hβ); 1.28–1.37 (m, 1H, 7-Hα); 1.44–1.57 (m, 6H, 1-Hα, 2-Hα, 11-Hα, 14-H, 15-Hβ, 16-Hα); 1.58–1.73 (m, 6H, 1-Hβ, 7-Hβ, 8-H, 9-H, 12-Hα, 15-Hα); 1.84–1.98 (m, 3H, 2-Hβ, 12-Hβ, 16-Hβ); 2.04 (dd, 1H, *J* = 13.6, 11.3 Hz, 4-Hα); 2.19 (ddd, 1H, *J* = 13.5, 4.3, 2.4 Hz, 4-Hβ); 3.75 (d, 1H, *J* = 5.9 Hz, 17-Hβ); 3.81 (d, 1H, *J* = 8.5 Hz, one of 19-H); 3.91 (d, 1H, *J* = 8.3 Hz, one of 19-H); 4.00 (d, 1H, *J* = 4.4 Hz, 6-Hα); 4.07 (tt, 1H, *J* = 11.4, 4.2 Hz, 3-Hα);

^1^H NMR (600 MHz) (ppm) (DMSO-*d*_6_) δ: 0.62 (s, 3H, 18-H); 1.07 (qd, 1H, *J* = 11.0, 6.6 Hz, 15-Hβ); 1.14 (qd, 1H, *J* = 12.2, 3.4 Hz, 11-Hβ); 1.27 (td, 1H, *J* = 13.7, 4.2 Hz, 1-Hα); 1.30–1.37 (m, 3H, 2-Hα, 7-Hα, 12-Hα); 1.37–1.43 (m, 1H, 11-Hα); 1.45–1.55 (m, 5H, 8-H, 9-H, 12-Hβ, 14-H, 15-Hα); 1.55–1.61 (m, 2H, 1-Hβ, 7-Hβ); 1.66–1.75 (m, 2H, 2-Hβ, 16-Hα); 1.81 (dd, 1H, *J* = 13.7, 11.2 Hz, 4-Hα); 1.94 (d, 1H, *J* = 14.3 6.0, 2.5 Hz, 16-Hβ); 2.01 (ddd, 1H, *J* = 13.6, 3.9, 2.0 Hz, 4-Hβ); 3.51 (dd, 1H, *J* = 5.4, 4.5 Hz, 17-Hβ); 3.70–3.77 (m, 2H, 3-Hα and one of 19-H); 3.79 (d, 1H, *J* = 8.6 Hz, one of 19-H); 3.93 (d, 1H, *J* = 4.5 Hz, 6-Hα); 4.31 (d, 1H, *J* = 4.3 Hz, 17-O*H*); 4.70 (d, 1H, *J* = 5.1 Hz,).

#### 3β-hydroxy-5α-chloro-17a-oxa-D-homo-6,19-oxidoandrostan-17-one (**4**)

71 mg of (**4**) was isolated after 3 days’ transformation of (**1**) (100 mg) in the *Penicillium commune* KCh W7 culture; after 6 days’ transformation of 100 mg of (**1**) in the *Beauveria bassiana* KCh BBT culture 52 mg of (**4**) was isolated.

^1^H NMR (600 MHz) (ppm) (CDCl_3_) δ: 1.06 (qd, 1H, *J* = 13.0, 3.5 Hz, 11-Hβ); 1.34 (s, 3H, 18-H); 1.43 (qd, 1H, *J* = 11.3, 5.8 Hz, 8-H); 1.45–1.49 (m, 2H, 1-Hα, 7-Hα); 1.54 (tt, 1H, *J* = 13.2, 8.6 Hz, 15-Hβ); 1.60–1.68 (m, 1H, 1-Hβ, 11-Hα, 14-H); 1.72 (dd, 1H, *J* = 12.6, 3.5 Hz, 12-Hα); 1.75 (ddd, 1H, *J* = 13.3, 5.6, 5.1 Hz, 2-Hα); 1.82–1.92 (m, 3H, 2-Hβ, 9-H, 15-Hα); 1.96 (ddd, 1H, *J* = 10.0, 5.0, 2.4 Hz, 7-Hβ); 2.01 (dt, 1H, *J* = 12.3, 3.2 Hz, 12-Hβ); 2.03 (dd, 1H, *J* = 13.6, 11.3 Hz, 4-Hα); 2.21 (ddd, 1H, *J* = 13.7, 4.3, 2.3 Hz, 4-Hβ); 2.57 (ddd, 1H, *J* = 19.0, 9.1, 8.4 Hz, 16-Hα); 2.68 (ddd, 1H, *J* = 19.0, 8.6, 2.4 Hz, 16-Hβ); 3.72 (d, 1H, *J* = 8.7 Hz, one of 19-H); 3.92 (dd, 1H, *J* = 8.7, 0.9 Hz, one of 19-H); 4.04 (d, 1H, *J* = 4.8 Hz, 6-Hα); 4.07 (tt, 1H, *J* = 11.1, 4.6 Hz, 3-Hα).

#### 3β,11α-dihydroxy-5α-chloro-17a-oxa-D-homo-6,19-oxidoandrostan-17-one (**5**)

After 6 days’ transformation of 100 mg of (**1**) in the *Beauveria bassiana* KCh BBT culture the isolation yield of (**5**) was 7 mg.

^1^H NMR (600 MHz) (ppm) (CDCl_3_) δ: 1.36 (s, 3H, 18-H); 1.41 (dd, 1H, *J* = 11.4, 5.8 Hz, 8-H); 1.43–1.49 (m, 1H, 7-Hα); 1.52 (tt, 1H, *J* = 13.4, 8.7 Hz, 15-Hβ); 1.66 (ddd, 1H, *J* = 13.1, 11.4, 4.4 Hz, 14-H); 1.73–1.82 (m, 2H, 1-Hα, 2-Hα, 12-Hα); 1.89 (tdd, 1H, *J* = 10.6, 4.3, 2.3 Hz, 15-Hα); 1.91–1.98 (m, 3H, 2-Hβ, 7-Hβ, 9-H); 2.03 (dd, 1H, *J* = 13.7, 11.4 Hz, 4-Hα); 2.20 (dt, 1H, *J* = 11.3, 3.4 Hz, 1-Hβ); 2.22 (ddd, 1H, *J* = 13.5, 4.1, 2.4 Hz, 4-Hβ); 2.31 (dd, 1H, *J* = 12.2, 4.6 Hz, 12-Hβ); 2.59 (dt, 1H, *J* = 19.1, 9.1 Hz, 16-Hα); 2.68 (ddd, 1H, *J* = 19.1, 8.8, 2.1 Hz, 16-Hβ); 3.59–3.65 (m, 1H, 11-Hβ); 3.76 (d, 1H, *J* = 9.0 Hz, one of 19-H); 4.01 (d, 1H, *J* = 4.8 Hz, 6-Hα); 4.02–4.07 (m, 2H, one of 19-H and 3-Hα).

#### 3β,11α-dihydroxy-5α-chloro-6,19-oxidoandrostan-17-one (**6**)

After 6 days’ transformation of 100 mg of (**1**) in the *Isaria farinosa* KCh KW1.1 culture the isolation yield of (**6**) was 5 mg.

^1^H NMR (600 MHz) (ppm) (CDCl_3_) δ: 0.92 (s, 3H, 18-H); 1.30 (dd, 1H, *J* = 12.2, 10.9 Hz, 12-Hα); 1.47 (dtd, 1H, *J* = 13.4, 11.8, 4.3 Hz, 2-Hα); 1.50–1.58 (m, 2H, 14-H, 15-Hβ); 1.74 (dt, 1H, *J* = 13.2, 5.0 Hz, 7-Hα); 1.78–1.84 (m, 3H, 1-Hα, 8-H, 9-H); 1.87–1.92 (m, 1H, 15-Hα); 1.93 (dm, 1H, *J* = 13.0 Hz, 2-Hβ); 1.98 (dd, 1H, *J* = 13.0, 11.0 Hz, 7-Hβ); 2.04 (dd, 1H, *J* = 13.7, 11.4 Hz, 4-Hα); 2.09–2.14 (m, 2H, 1-Hβ, 16-Hα); 2.17 (dd, 1H, *J* = 12.8, 5.2 Hz, 12-Hβ); 2.20 (ddd, 1H, *J* = 13.7, 4.2, 2.5 Hz, 4-Hβ); 2.47 (dd, 1H, *J* = 19.6, 8.2 Hz, 16-Hβ); 3.78 (td, 1H, *J* = 10.2, 4.9 Hz, 11-Hβ); 3.82 (d, 1H, *J* = 8.9 Hz, one of 19-H); 4.01 (tt, 1H, *J* = 11.4, 4.4 Hz, 3-Hα); 4.02 (d, 1H, *J* = 4.8 Hz, 6-Hα); 4.05 (d, 1H, *J* = 8.9 Hz, one of 19-H).

^1^H NMR (600 MHz) (ppm) (DMSO-*d*_6_) δ: 0.83 (s, 3H, 18-H); 1.15 (dd, 1H, *J* = 12.6, 10.5 Hz, 12-Hα); 1.34 (dtd, 1H, *J* = 13.4, 11.9, 4.2 Hz, 2-Hα); 1.40–1.48 (m, 2H, 14-H, 15-Hβ); 1.58–1.67 (m, 4H, 1-Hα, 7-Hα, 8-H, 9-H); 1.68–1.74 (m, 1H, 2-Hβ); 1.74–1.79 (m, 1H, 15-Hα); 1.82 (dd, 1H, *J* = 13.0, 11.6 Hz, 7-Hβ); 1.83–1.88 (m, 2H, 4-Hα, 12-Hβ); 2.01–2.08 (m, 3H, 1-Hβ, 4-Hβ, 16-Hα); 2.36 (dd, 1H, *J* = 18.9, 7.9 Hz, 16-Hβ); 3.59–3.65 (m, 1H, 11-Hβ); 3.66–3.72 (m, 1H, 3-Hα); 3.82 (d, 1H, *J* = 8.9 Hz, one of 19-H); 3.88 (d, 1H, *J* = 8.9 Hz, one of 19-H); 3.95 (d, 1H, *J* = 4.6 Hz, 6-Hα); 4.27 (d, 1H, *J* = 6.9 Hz, 3-O*H*); 4.67 (d, 1H, *J* = 5.1 Hz, 11-O*H*).

#### 3β,11α,19-trihydroxy-5α-chloro-6,19-oxidoandrostan-17-one (**7**)

After 6 days’ transformation of 100 mg of (**1**) in the *Isaria farinosa* KCh KW1.1 culture the isolation yield of (**7**) was 38 mg.

^1^H NMR (600 MHz) (ppm) (CDCl_3_) δ: 0.91 (s, 3H, 18-H); 1.24–1.33 (m, 2H, 7-Hα, 12-Hα); 1.47 (td, 1H, *J* = 12.4, 5.8 Hz, 2-Hα); 1.49–1.55 (m, 2H, 14-H, 15-Hβ); 1.58 (t, 1H, *J* = 10.3 Hz, 9-H); 1.71–1.77 (m, 2H, 7-Hβ, 8-H); 1.86–1.94 (m, 2H, 2-Hβ, 15-Hα); 1.97 (ddd, 1H, *J* = 15.0, 11.7, 3.2 Hz, 1-Hα); 2.13 (dt, 1H, *J* = 19.4, 8.8 Hz, 16-Hα); 2.21 (dd, 1H, *J* = 12.7, 5.2 Hz, 12-Hβ); 2.27 (d, 1H, *J* = 14.3 Hz, 4-Hα); 2.35 (ddd, 1H, *J* = 15.0, 12.3, 5.7 Hz, 1-Hβ); 2.47 (dd, 1H, *J* = 19.4, 8.0 Hz, 16-Hβ); 2.62 (dt, 1H, *J* = 14.3, 3.6 Hz, 4-Hβ); 3.90 (td, 1H, *J* = 10.2, 5.2 Hz, 11-Hβ); 3.97–4.01 (m, 2H, 3-Hα, 6-Hα); 5.27 (s, 1H, 19-H).

#### 3β,11α-dihydroxy-5α-chloro-6,19-oxidoandrostan-17,19-dione (**8**)

After 6 days’ transformation of 100 mg of (**1**) in the *Isaria farinosa* KCh KW1.1 culture the isolation yield of (**8**) was 4 mg.

^1^H NMR (600 MHz) (ppm) (DMSO-*d*_6_) δ: 0.71 (s, 3H, 18-H); 0.90–0.97 (m, 1H, 7-Hα); 1.15 (dd, 1H, *J* = 12.6, 10.5 Hz, 12-Hα); 1.42 (dtd, 1H, *J* = 13.4, 11.8, 4.1 Hz, 2-Hα); 1.45–1.50 (m, 1H, 15-Hβ); 1.52 (td, 1H, *J* = 10.8, 5.0 Hz, 14-H); 1.58–1.67 (m, 3H, 1-Hα, 4-Hα, 8-H); 1.67–1.73 (m, 2H, 2-Hβ, 9-H); 1.74–1.80 (m, 2H, 7-Hβ, 15-Hα); 1.86 (dd, 1H, *J* = 12.9, 5.2 Hz, 12-Hβ); 2.04 (dt, 1H, *J* = 19.3, 8.9 Hz, 16-Hα); 2.28 (ddd, 1H, *J* = 14.2, 3.6, 2.9 Hz, 4-Hβ); 2.37 (dd, 1H, *J* = 19.0, 7.9 Hz, 16-Hβ); 2.40 (dt, 1H, *J* = 11.3, 3,4 Hz, 1-Hβ); 3.54–3.62 (m, 2H, 3-Hα, 11-Hβ); 4.59 (d, 1H, *J* = 6.6 Hz, 3-O*H*); 4.68 (d, 1H, *J* = 4.6 Hz, 6-Hα); 4.80 (d, 1H, *J* = 5.2 Hz, 11-O*H*).

### Interpretation of results

The aim of the conducted research was to evaluate the ability of strains (with defined activity in the transformation of steroid compounds) to transform a steroid having an oxirane bridge and to obtain new derivatives of 3β-acetyloxy-5α-chloro-6,19-oxidoandrostan-17-one (**1**). The substrate was obtained from the resources of the Department of Chemistry of the Wrocław University of Environmental and Life Sciences, and due to its structure it can be a precursor to many compounds with interesting biological properties [10–14].

A total of twelve strains of filamentous fungi belonging to ten species were used as biocatalysts in this experiment: *Beauveria bassiana* KCh BBT and KCh J1, *Isaria fumosorosea* KCh J2, *Isaria farinosa* KCh KW1.1, *Beauveria caledonica* KCh J3.3 and KCh J3.4, *Aspergillus niger* KCh M1, *Penicillium commune* KCh W7, *Penicillium chrysogenum* KCh S4, *Mucor hiemalis* KCh W2, *Fusarium acuminatum* KCh S1 and *Trichoderma atroviride* KCh TRW. In the cultures of most of the tested biocatalysts, the test compound **1** was hydrolysed to the corresponding 3β-hydroxy-5α-chloro-6,19-oxidoandrostan-17-one (**2**). Only a few tested strains converted compound **2** into further products. Based on TLC and GC analyses, it was observed that in the cultures of *I. farinosa* KCh KW 1.1, *P. chrysogenum* KCh S4, *M. hiemalis* KCh W2, *P. commune* KCh W7 and *B. bassiana* KCh BBT products with higher polarity were created (Table 1).

**Table 1:** Products’ accumulation during the conversions of the substrate (**1**)

Microorganism	Compounds found in the reaction mixture	Retention time by GC (min)	Biotransformation time (days)			
			1	3	6	10
*Mucor hiemalis* KCh W2	**2**	3.94	67	36	11	7
	**3**	4.07	21	56	73	68
	**1**	4.61	11	1	–	–
*Penicillium commune* KCh W7	**2**	3.94	9	1	–	–
	**1**	4.61	91	2	–	–
	**4**	6.47	–	97	100	100
*Penicillium chrysogenum* KCh S4	**2**	3.94	18	–	–	–
	**1**	4.61	82	1	–	
	**4**	6.47	–	99	100	100
*Beauveria bassiana* KCh BBT	**2**	3.94	40	78	–	–
	**1**	4.61	45	–	–	–
	**4**	6.47	15	12	84	81
	**5**	8.44	–	–	9	9
*Beauveria bassiana* KCh J1	**2**	3.94	7	14	15	21
	**1**	4.61	93	86	85	79
*Isaria fumosorosea* KCh J2	**2**	3.94	22	29	52	64
	**1**	4.61	78	71	48	36
*Beauveria caledonica* KCh J3.3	**2**	3.94	18	31	31	31
	**1**	4.61	75	64	65	65
*Beauveria caledonica* KCh J3.4	**2**	3.94	94	100	100	100
	**1**	4.61	6	–	–	–
*Fusarium acuminatum* KCh S1	**2**	3.94	72	90	89	87
	**1**	4.61	25	5	2	2
*Trichoderma atroviride* KCh TRW	**2**	3.94	72	93	92	94
	**1**	4.61	24	2	–	–
*Isaria farinosa KCh* KW1.1	**2**	3.94	3	0	–	–
	**1**	4.61	0	–	–	–
	**6**	6.59	42	26	9	2
	**7**	5.11	53	59	65	58
	**8**	8.72	0	1	6	9

Biotransformation conditions: 100 mL of cultivation medium (3% glucose, 1% aminobac) in 300 mL Erlenmeyer flasks, 25 °C, 150 r/min for 10-day transformation. Data are the average of 3 independent experiments. Standard errors were in the range: 0–5

**Fig. 1:**
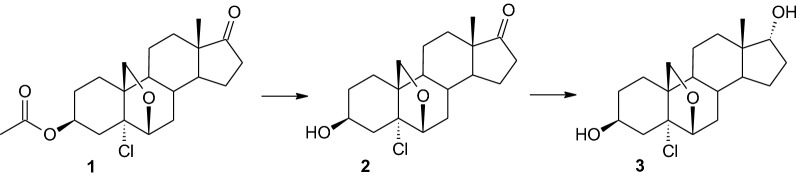
Transformations of **1** in the *Mucor hiemalis* KCh W2 culture. Biotransformation conditions: 100 mL of cultivation medium (3% glucose, 1% aminobac) in 300 mL Erlenmeyer flasks, 25 °C, 150 r/min for 10-day transformation

Two products were obtained in the culture of the *Mucor hiemalis* KCh W2 strain. One is compound **2** with a retention time of 3.94 min – a product of hydrolysis of the acetyl group at C-3, which is observed in all biotransformations presented in this research. The other (**3**) is characteristic only for this strain. It is a product of hydrolysis followed by reduction of the carbonyl group at C-17. Its retention time is 4.07 min and its isolated yield from preparative biotransformation is 38%. Due to the decreasing percentage of compound **2**, it can be assumed that it is an intermediate during this biotransformation (Fig. 1).

A characteristic difference between the ^1^H NMR spectrum obtained for compound **2** and the spectrum of the substrate (**1**) is the absence of a singlet from acetyl group protons. The position of the signal from the C-3 proton has also changed, moving upfield from 5.10 ppm to 4.07 ppm. In the slightly higher field on the spectrum of the discussed product, there are also signals from 2-Hβ and 4-Hα protons. The ^13^C NMR spectrum of compound **2** also does not contain signals from the carbons of the acetyl group. Moreover, the signal from the carbon C-3 was slightly shifted towards the higher field, while towards the lower signal from the carbons C-2 and C-4, confirming the structural changes within this molecule (Table 2, Additional file 1).

**Table 2:** ^13^C NMR chemical shifts of products

Atom number	1^a^	1^b^	2^a^	3^a^	3^b^	4^a^	5^a^	6^a^	6^b^	7^a^	8^b^
1	23.25	22.84	23.61	23.91	23.30	23.40	25.88	25.73	25.46	19.03	24.95
2	26.99	26.33	30.83	30.89	30.49	30.97	30.87	30.72	30.53	26.44	29.63
3	69.19	68.67	66.41	66.53	64.45	66.34	66.03	66.04	64.27	64.52	63.36
4	40.26	39.66	44.03	44.14	43.84	43.72	44.44	44.69	44.65	48.81	45.80
5	75.67	76.70	76.84	76.31	77.49	75.61	75.83	76.47	77.80	69.50	74.55
6	82.01	80.54	82.08	82.36	80.98	81.76	81.62	81.99	80.85	82.63	80.39
7	31.52	31.29	31.59	31.59	31.45	30.70	31.22	31.25	31.13	28.96	32.53
8	33.05	32.40	33.09	33.60	33.06	35.73	33.37	30.86	30.34	30.81	31.13
9	46.76	46.57	46.92	46.92	46.43	45.64	52.50	53.55	52.91	49.34	52.73
10	45.95	45.27	45.92	45.82	45.11	45.71	81.41	47.64	47.08	46.68	47.56
11	21.81	31.07	21.91	22.15	21.49	23.34	68.72	68.73	66.88	68.42	65.76
12	30.83	30.48	30.91	32.23	32.05	39.32	50.39	43.36	42.83	43.63	42.38
13	48.36	47.70	48.40	46.02	45.39	83.08	47.45	48.62	47.95	48.37	50.50
14	49.57	48.95	49.65	46.93	46.60	44.84	44.10	48.69	48.27	48.78	47.79
15	21.43	20.96	21.49	23.71	23.43	19.55	19.59	21.34	20.91	21.33	20.55
16	35.85	35.28	35.88	32.69	32.08	28.63	28.58	35.94	35.49	35.94	35.42
17	220.26	219.18	220.38	79.71	77.54	171.39	170.90	218.80	218.50	218.64	217.87
18	14.31	13.86	14.33	17.55	17.31	20.47	21.41	15.09	14.63	14.87	14.38
19	68.65	67.62	68.76	68.70	67.70	68.30	69.21	69.74	68.74	101.94	174.95
–CO*C*H_3_	21.46	21.02					–	–	–		
–*C*OCH_3_	170.45	169.80					–	–	–		

^a^Dissolved in CDCl_3_

^b^In DMSO-*d*6

Mass spectroscopy analysis was performed to confirm the presence of a chlorine atom in the resulting product molecule. According to calculations based on the molecular formula of compound **2** (C_19_H_27_O_3_Cl), its mass is equal to 338.45 Da. The MS spectrum shows a signal corresponding to a compound with a molecular weight of 338 Da, which confirms the presence of a chlorine atom in the structure of the product in question.

In the ^1^H NMR spectrum obtained for compound **3**, no signals from protons of the acetyl group were observed, and the multiplet derived from the carbon C-3 proton shifted from 5.10 ppm to 4.07 ppm. However, a doublet from the proton at C-17 (3.75 ppm) appeared, which indicates a reduction of the carbonyl group to hydroxyl. All the above is confirmed by the ^13^C NMR spectrum, in which the signal from the carbon C-17 has shifted from 220.26 ppm to 79.71 ppm. Signals from the 16-Hα and 16-Hβ protons shifted from 2.07 ppm and 2.43 ppm to 1.44–1.57 ppm and 1.84–1.98 ppm, respectively (Table 2, Additional file 1). The shape and location of the signal coming from the proton located at the C-17 carbon indicate the location of the hydroxyl group at the carbon in the axial position. Due to incomplete dissolution of this compound in CDCl_3_, NMR analysis was also performed in DMSO-*d*_6_ as a solvent (Table 2).

In the case of 3β,17α-dihydroxy-5α-chloro-6,19-oxidoandrostane (**3**), mass spectroscopy analysis also confirmed the presence of a chlorine atom in the molecule. Based on the molecular formula of product **3** (C_19_H_28_O_3_Cl), its molecular weight is 339.45 Da. The MS spectrum shows a signal corresponding to a compound with a molecular weight of 322.2 Da (Additional file 1). The difference (~ 17 Da) results from the detachment of the hydroxyl group from the product molecule during the analysis.

It is known from our previous study that the *M. hiemalis* KCh W2 strain carries out hydroxylation reactions at the 7α position and reduction of the C-17 carbonyl group to form 17α-hydroxysteroid [58]. In the case of biotransformation of the tested substrate (**1**), no hydroxyl attachment to the C-7 carbon was observed, which may be due to the effect of the oxirane bridge on the structure of the molecule. However, a C-17 carbonyl group reduction product was obtained. Steroid compounds with a hydroxyl group at the 17α position are found in lower concentrations in the human body than their 17β-epimers. Their functions are still unclear, but the mere fact of identifying these compounds in mammals may indicate their importance [68, 69]. Some of them are already known, for example 17α-androstenediol inhibits proliferation and mediates apoptosis in tumour cells of murine and human origin. In contrast, its epimer 17β-androstenediol does not [70–72]. The antiproliferative functions of 17α-androstenediol are not dependent on either the estrogen or androgen receptors [73, 74]. 17α-androstenes protect the host from lethal infection by DNA or RNA viruses such as herpes virus type 2, coxsackie virus B4, influenza, and arthropod borne viruses [71]. It has been proved that 3β,7α,17α-trihydroxyandrost-5-ene with such location of the hydroxyl groups has the most potent anticancer activity toward lymphoma [75]. Also, 17α-estrogens and their biological activity were observed. 17α- Estradiol has neuroprotective potential in an in vivo model of injury to the immature brain [76] as well as in Alzheimer’s disease [77].

17α-Steroids have interesting biological activities, but they are difficult to synthesise by chemical methods. Hydroxyderivatives with a hydroxyl group at the 17β position are obtained using chemical catalysts (e.g. lithium aluminium hydride). Epitestosterone may be obtained with 34% yield by two-step inversion of the hydroxyl group at the 17-position via tosylation [78]. The decarboxylation of unsaturated steroidal acids leads in four steps to 17α-steroids, with about 30% yield [79]. Mouse, rat, rabbit and pig 17α-hydroxysteroid dehydrogenases are known and may be over–expressed in *Escherichia coli* cells to produce 17α-hydroxysteroids [80]. Using microbiological methods to reduce the carbonyl group in C-17, 17β stereoisomers are usually obtained [81]. The use of dehydrogenase from the *M. hiemalis* KCh W2 strain may prove to be an effective solution for obtaining 17α-hydroxysteroids.

Strains of the genus *Penicillium*, i.e. *P. commune* KCh W7 and *P. chrysogenum* KCh S4, transformed the test compound analogously. In both cases, 3β-acetyloxy-5α-chloro-6,19-oxidoandrostan-17-one (**1**) was almost completely converted in about three days (Table 1). The main product of the transformations was compound **4**, with a retention time of approximately 6.50 min. Additionally, compound **2**—a hydrolysis product of the tested substrate **1**—was identified on the chromatograms after 24 h of transformation. In order to isolate the main product (**4**), increased scale transformation of the test compound was performed in the culture of the *P. commune* KCh W7 strain (isolated yield—71%). The isolated product **4** turned out to be a product of hydrolysis of the C-3 acetyl group and Baeyer–Villiger oxidation of the D ring.

**Fig. 2:**
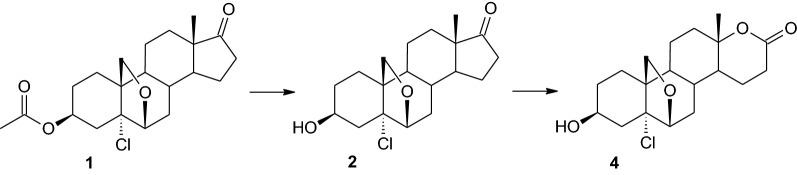
Baeyer-Villiger oxidation by *Penicillium commune* KCh W7 and *Penicillium chrysogenum* KCh S4 culture. Biotransformation conditions: 100 mL of cultivation medium (3% glucose, 1% aminobac) in 300 mL Erlenmeyer flasks, 25 °C, 150 r/min for 10-day transformation

NMR spectra of 3β-hydroxy-5α-chloro-17α-oxa-D-homo-6,19-oxidoandrostan-17-one (**4**), as well as previous products, do not have signals derived from protons and carbons of the acetyl group. Also, in this case, signals from C-3 protons were shifted towards the higher field (from 5.10 ppm to 4.07 ppm). In relation to the spectrum for compound **2**, the change is also visible in the position of signals coming from the surroundings of the carbonyl group. The C-17 carbon signal is shifted from 220.26 ppm to 171.39 ppm, C-18 from 14.31 ppm to 20.47 ppm, C-16 carbon from 35.85 ppm to 28.63 ppm and C-13 carbon from 48.36 ppm to 83.08 ppm (Table 2, Additional file 1). Mass spectroscopy analysis of 3β-hydroxy-5α-chloro-17α-oxa-D-homo-6,19-oxidoandrostan-17-one (**4**) confirmed the structure of this compound. The MS spectrum shows a signal corresponding to a compound with a mass of 355.2 Da (Additional file 1). According to the molecular formula of product **4** (C_19_H_27_O_4_Cl), its mass is 354.45 Da. Identification of the transformation products of 3β-acetyloxy-5α-chloro-6,19-oxidoandrostan-17-one (**1**) allowed us to develop a putative metabolic pathway carried out by *P. commune* KCh W7 (Fig. 2).

The biotransformation results obtained for the *Penicillium commune* KCh W7 and *Penicillium chrysogenum* KCh S4 strains coincide with the results described for other substrates transformed by these biocatalysts. Both strains perform Baeyer–Villiger oxidation of the D ring of steroids [58], which has also been achieved in this study. In addition, during the transformation of DHEA, these biocatalysts oxidised the 3β-hydroxyl group to the carbonyl group. For the tested substrate, only hydrolysis of the acetyl group was observed. As suggested above, the reason for differences in the metabolism of the tested substrate may be a different spatial structure of the steroid, resulting from the presence of an oxirane bridge, or the influence of a highly electronegative chlorine atom.

**Fig. 3:**
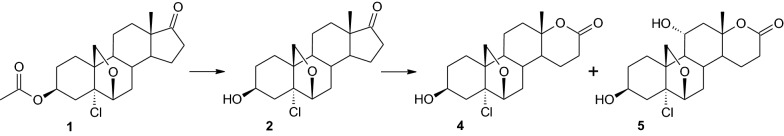
Transformations of the substrate (**1)** in *Beauveria bassiana* KCh BBT culture. Biotransformation conditions: 100 mL of cultivation medium (3% glucose, 1% aminobac) in 300 mL Erlenmeyer flasks, 25 °C, 150 r/min for 10 day transformation

The analysis of biotransformation carried out by the *Beauveria bassiana* KCh BBT strain showed that after the third day, all the substrate **1** was transformed. In tests from the first and third day, compound **2**, with a retention time of 3.94 min, was observed. It was further transformed by the sixth day. Finally, after 10 days, two potential substrate biotransformation products with a retention time of 6.52 min and 8.44 min were detected (Additional file 1). To determine their structures, the transformation was carried out at an increased scale. Based on the results obtained, it was found that the compound with a retention time of 6.52 min is a product of Baeyer–Villiger oxidation—3β-hydroxy-5α-chloro-17α-oxa-D-homo-6,19-oxidoandrostan-17-one (**4**). The other compound, with a retention time of 8.44 min, is 3β,11α-dihydroxy-5α-chloro-17α-oxa-D-homo-6,19-oxidoandrostan-17-one (**5**)—with 7% of isolated yield. It results from the hydrolysis of the acetyl group at the carbon C-3, hydroxylation at the 11α position and Baeyer–Villiger oxidation of the D ring (Fig. 3). This compound has not been described in the literature.

In the ^1^H NMR spectrum made for the obtained product **5**, no signal from the acetyl protons was observed, which is visible as a singlet at 2.03 ppm in the spectrum of the substrate. The ^13^C NMR spectrum also indicates the absence of carbons from the –COCH_3_ group, whose signals should be present at 21.46 ppm (–CH_3_) and 170.45 ppm (carboxyl carbon). These observations prove the disconnection of this group from the substrate molecule. The multiplet derived from C-3 carbon protons has shifted towards the higher field from 4.92 ppm to 4.02 ppm, similarly to the previously discussed products. The difference between the spectra of the substrate and product **5** is also visible in the signals coming from protons at the C-11 carbon. In the spectrum of the substrate they are located in the range of 1.16–1.58 ppm, while in the spectrum of product **5** they are located in a much lower field around 3.59–3.65 ppm, which is the result of the unfolding action of the attached hydroxyl group. Also, signals from carbons C-9, C-10, C-11 and C-12 in the ^13^C NMR spectrum shifted towards the lower field. Additionally, more proton signals were shifted: C-16 (Hα from 2.07 ppm to 2.59 ppm and Hβ from 2.43 ppm to 2.68 ppm), C-18 (from 0.81 ppm to 1.36 ppm) and the signal from the carbon C-17 (from 220.26 ppm to 170.90 ppm), which is characteristic for lactonisation of the steroid D ring (Table 2, Additional file 1).

The result of the transformation carried out by *B. bassiana* KCh BBT partly overlaps with the results obtained earlier, where DHEA transformations in cultures of strains from the genus *Beauveria* were analysed [27]. In that work, the *B. bassiana* KCh BBT strain, similar to the present study, performed Baeyer–Villiger oxidation of the D ring and hydroxylation at the 11α position. Also, the introduction of a hydroxyl group at the 7α position and its subsequent oxidation has been described, but it was not observed in the case of transformation of 3β-acetyloxy-5α-chloro-6,19-oxidoandrostan-17-one (**1**). Moreover, the introduction of a hydroxyl group in the 7α position and its subsequent oxidation has been described in the literature, but it was not observed in the case of transformation of 3β-acetyloxy-5α-chloro-6,19-oxidoandrostan-17-one (**1**). It is possible that the presence of an oxirane bridge and a chlorine atom in the close vicinity of the C-7 carbon causes that the test compound does not adapt to the active site of the enzymes responsible for this reaction.

*Beauveria bassiana* KCh J1 and *Isaria fumosorosea* KCh J2 strains gave similar biotransformation results (Table 1). In both cases, only one product was observed with a retention time of approximately 3.94 min—an acetyl group hydrolysis product (3β-hydroxy-5α-chloro-6,19-oxidoandrostan-17-one (**2**)). However, these microorganisms did not entirely convert the substrate. After 10 days of transformation in the cultures of *Beauveria bassiana* KCh J1 and *Isaria fumosorosea* KCh J2, the substrate accounted for as much as 79% and 36% of reaction mixtures, respectively. Compared to the results of our previous studies, testing the ability of these strains to transform steroids, this result was surprising. The strain *B. bassiana* KCh J1 transformed DHEA in less than 24 h by hydroxylation reaction at the 7α and 11α position [27]. If the necessary condition for transformation of steroids by this strain is the introduction of the hydroxyl group into the C-7 carbon first, it is possible that, as in the case of the *B. bassiana* KCh BBT strain, the oxirane bridge and chlorine atom change the spatial structure of the molecule so much that it does not match the active site of the relevant enzyme.

In the case of the *I. fumosorosea* KCh J2 strain, DHEA, androstenedione, adrenosterone and 17α-methyltestosterone were transformed, and all compounds except adrenosterone were transformed within 24 h. The strain carried out hydroxylation reactions of these substrates at the 6β, 7α, 7β, 12β, 15β positions, Baeyer–Villiger oxidation of the D ring and oxidation of the hydroxyl group [38]. While the lack of specific hydroxylation products can be justified—C-6 and C-7 carbons are close to the oxirane bridge and chlorine atom, and hydroxylation at C-12 and C-15 carbons occurred only in a compound without a carbonyl group at the C-17 carbon—the lack of Baeyer–Villiger oxidation is surprising, especially as it was possible to isolate the products of this reaction for other strains tested here. The hydroxylation at the 7α or 7β positions is crucial for Baeyer–Villiger oxidation of DHEA in the *I. fumosorosea* KCh J2 culture [38], and absence of hydroxylation prevents the chain of reactions as mentioned above.

Despite belonging to one species, strains *Beauveria caledonica* KCh J3.3 and KCh J3.4 demonstrated different capacities for converting 3β-acetyloxy-5α-chloro-6,19-oxidoandrostan-17-one (**1**). In both cases, only hydrolysis product **2** was produced. However, in the *B. caledonica* KCh J3.3 strain culture, significantly lower substrate conversion was observed. Over 10 days, the product content in the *B. caledonica* KCh J3.3 culture reached slightly over 30%. Additionally, two other potential biotransformation products with retention times of 6.43 min and 7.29 min were observed on the chromatograms, but neither of them constituted more than 7% of the post-reaction mixture, and therefore preparative biotransformation was abandoned in order to isolate them (Table 1). For *B. caledonica* KCh J3.4, unreacted substrate **1** was only observed in the mixture after 1 day of biotransformation. No hydroxylation products were observed in any of the biotransformations with strains of *B. caledonica* species, which were expected based on the results of another experiment using these biocatalysts [27]. *B. caledonica* KCh J3.3 and KCh J3.4 strains have previously been described as capable of hydroxylation at the 7α, 7β, 11α positions and oxidation of the hydroxyl group at the C-7 carbon of the steroid skeleton. In addition, *B. caledonica* KCh J3.4 strain hydrogenated the double bond. Moreover, the substrate (DHEA) was entirely transformed by both strains within 24 h. At this stage, it is difficult to determine the type of influence of the oxirane bridge and chlorine atom on the course of catalytic processes.

Similar results were obtained for the strains *Fusarium acuminatum* KCh S1 and *Trichoderma atroviride* KCh TRW. As in the case of *B. caledonica* KCh J3.4, only the acetyl group hydrolysis product **2** (retention time 3.94 min) was obtained, which after ten days of biotransformation constituted approximately 90% of the reaction mixtures (Table 1). The strain *Fusarium acuminatum* KCh S1 has already been used for transformation of steroids and has demonstrated the capacity for effective stereoselective hydroxylation at the 7α position [58]. However, 3β-acetyloxy-5α-chloro-6,19-oxidoandrostan-17-one (**1**) did not undergo this reaction. Again, the causes can be traced to the presence of an oxirane bridge or chlorine atom in the molecule of the substrate being examined.

Due to a large number of products observed on chromatograms made from samples taken from *Aspergillus niger* KCh M1 culture, they proved to be quite challenging to interpret. After the third day of transformation, about ten compounds appeared in the mixture with retention times in the range of 4.00–6.50 min. Most of them constituted 2 to 4% of the reaction mixture. In the following days, the number of detected compounds increased. It is likely that the peaks observed are derived both from biotransformation products and from the microorganism’s metabolites, which unfortunately have retention times in terms of potential transformation products. Also, a significant percentage of unreacted substrate (37% of the reaction mixture) was observed after 10 days. The above data were the reason for not isolating potential products.

**Fig. 4:**
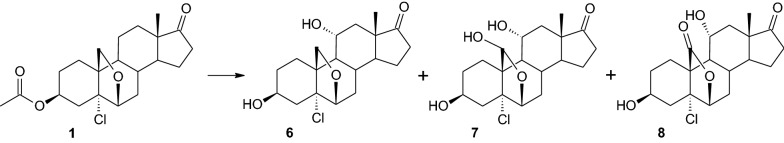
Products obtained during biotransformation of **1** in the *Isaria farinosa* KCh KW1.1 culture. Biotransformation conditions: 100 mL of cultivation medium (3% glucose, 1% aminobac) in 300 mL Erlenmeyer flasks, 25 °C, 150 r/min for 10-day transformation

In the culture of the *Isaria farinosa* KCh KW1.1 strain, three 11α-hydroxy derivatives were obtained. They are the result of consecutive hydrolysis of the acyl group at C-3, 11α-hydroxylation (3β,11α-dihydroxy-5α-chloro-6,19-oxidoandrostan-17-one (**6**)) followed by hydroxylation of the C-19 carbon atom (3β,11α,19-trihydroxy-5α-chloro-6,19-oxidoandrostan-17-one (**7**)) and its further oxidation leading to the formation of lactone (3β,11α-dihydroxy-5α-chloro-6,19-oxidoandrostan-17,19-dione (**8**)) (Fig. 4, Table 1). As a result of larger-scale biotransformation, the products were purified, and their structures determined. Compound **2** (hydrolysis product) was identified as an intermediate, as in all previously described biotransformations. In the ^13^C NMR spectrum, 3β,11α-dihydroxy-5α-chloro-6,19-oxidoandrostan-17-one (**6**) compared to compound **2** has a characteristic signal for hydroxyl-associated carbon atoms at 68.73 ppm. This carbon couples to a proton located on the ^1^H NMR spectrum at a position of 3.78 ppm. Location, multiplicity, and couplings with protons present at C-12 and C-9 carbon visible in the COSY spectrum clearly show the presence of the hydroxylation group present at position 11α (Additional file 1).

The main product formed in the culture of the *Isaria farinosa* KCh KW1.1 strain is 3β,11α,19-trihydroxy-5α-chloro-6,19-oxidoandrostan-17-one (**7**) obtained with 38% isolated yield. In the COSY spectrum of compound **7**, there is a signal from C-11, which is coupled to the characteristic proton 11β. The signal from the C-19 carbon was shifted from 69.74 ppm (position for compound **6**) to 101.94 ppm. This location, characteristic for a hemiacetal carbon atom, indicates the presence of an additional hydroxyl group at carbon C-19. Confirmation of the presence of this moiety is the coupling of C-19 carbon with the singlet located at 5.27 ppm derived from one proton.

In the culture of the *Isaria farinosa* KCh KW1.1 strain, after a prolonged process, the hydroxyl group at the C-19 of compound **7** undergoes oxidation leading to lactone—3β,11α-dihydroxy-5α-chloro-6,19-oxidoandrostan-17,19-dione (**8**). Due to the low solubility in CDCl_3_ of this compound, NMR analysis was performed in DMSO-*d*_6_ as a solvent. Most of the signals visible in ^1^H and ^13^C NMR spectra made for this compound are in positions analogous to the other two 11α-hydroxy derivatives. However, the shift of the signal from C-19 towards the lower field to the value of 174.95 ppm indicates the presence of a lactone group in the structure of this product (Table 2).

Our earlier studies described the ability of this strain to efficient hydroxylation DHEA to its 7α- and 7β-hydroxy derivatives. Progesterone was transformed into 6β,11α-dihydroxy derivative by this biocatalyst [42]. Most likely, the presence of an oxygen substituent located at the 6β position in the structure of the tested compound causes that it, similarly to progesterone, undergoes hydroxylation leading to obtaining the corresponding 11α-hydroxy derivatives.

## Conclusions

Most of the tested strains efficiently performed hydrolysis of the acetyl group at the C-3 carbon. As a result of the conducted experiments, seven new steroid compounds not previously described in the literature were obtained.

During the biotransformation of the test compound (3β-acetyloxy-5α-chloro-6,19-oxidoandrostan-17-one (**1**)) in the culture of the *Mucor hiemalis* KCh W2 strain, products of acetyl group hydrolysis and reduction of the carbonyl group at the C-17 (3β-hydroxy-5α-chloro-6,19-oxidoandrostan-17-one (**2**) and 3β,17α-dihydroxy-5α-chloro-6,19-oxidoandrostan (**3**)) were obtained. The strain *M. hiemalis* KCh W2 due to the stereoselective reduction of the carbonyl group may prove to be a useful catalyst for the synthesis of 17α-hydroxysteroids, which are difficult to obtain by chemical methods. *Penicillium commune* KCh W7 and *P. chrysogenum* KCh S4 strains hydrolysed the acetyl group and oxidised the D ring to lactone, resulting in 3β-hydroxy-5α-chloro-17α-oxa-D-homo-6,19-oxidoandrostan-17-one (**4**). The *Beauveria bassiana* KCh BBT strain is capable of carrying out hydrolysis of an acetyl group, hydroxylation at the 11α position and Baeyer–Villiger oxidation of the D ring of the tested substrate, resulting in 3β,11α-dihydroxy-5α-chloro-17α-oxa-D-homo-6,19-oxidoandrostan-17-one (**5**). No expected C-7 hydroxylation products were observed, among others in cultures of *Beauveria bassiana* KCh BBT, *B. bassiana* KCh J1, *Isaria fumosorosea* KCh J2, *B. caledonica* KCh J3.3, *B. caledonica* KCh J3.4, *Mucor hiemalis* KCh W2 and *Fusarium acuminatum* KCh S1. Hydroxylation of C-7 carbon is probably the critical stage of enzymatic transformations carried out by *B. caledonica* KCh J3.3, *B. caledonica* KCh J3.4, *B. bassiana* KCh J1 and *I. fumosorosea* KCh J2, the lack of which resulted in arresting the cascade changes observed for DHEA. The presence of an oxirane bridge that causes skeleton deflection between the A and B rings of the steroid and the electronegative chlorine atom in the steroid molecule affects the ability of biocatalysts to transform the tested substrate (especially hydroxylation at positions 7α and 7β).

## Supplementary information

**Additional file 1.**  Spectral data of the substrate and all obtained products.

## Data Availability

All data generated or analysed during this study are included in this published article and its additional file.
