# Supplementary material for: New 6,19-oxidoandrostan derivatives obtained by biotransformation in environmental filamentous fungi cultures
Source: Microb Cell Fact. 2020 Feb 17;19:37. doi: 10.1186/s12934-020-01303-6 (PMC7026961; doi:10.1186/s12934-020-01303-6)

Additional file 1

New 6,19-oxidoandrostan derivatives obtained by biotransformation in environmental filamentous fungi cultures

Ewa Kozłowska,*[a] Agata Matera,[a] Jordan Sycz,[a] Anna Kancelista,[b] Edyta Kostrzewa-Susłow,[a] Tomasz Janeczko*[a]

[a] Department of Chemistry, Wrocław University of Environmental and Life Sciences, Norwida 25, 50-375 Wrocław, Poland.
[b] Department of Biotechnology and Food Microbiology, Wrocław University of Environmental and Life Sciences, Chełmońskiego 37, 51-630 Wrocław, Poland.
Corresponding authors at: Department of Chemistry, Wrocław University of Environmental and Life Sciences, Norwida 25, 50-375 Wrocław, Poland.
E-mail addresses: e.a.kozlowska@gmail.com (E. Kozłowska), janeczko13@interia.pl (T. Janeczko)

Contents:

Fig.S1. GC-MS spectra of 3β-acetyloxy-5α-chloro-6,19-oxidoandrostan-17-one (**1**)
Fig.S2. ^{1}H NMR spectral of 3β-acetyloxy-5α-chloro-6,19-oxidoandrostan-17-one (**1**) ($CDCl_3$, 600 MHz)
Fig.S3. ^{13}C NMR spectral of 3β-acetyloxy-5α-chloro-6,19-oxidoandrostan-17-one (**1**) ($CDCl_3$, 151 MHz)
Fig.S4. HSQC spectral of 3β-acetyloxy-5α-chloro-6,19-oxidoandrostan-17-one (**1**) ($CDCl_3$, 151 MHz)
Fig.S5. COSY spectral of 3β-acetyloxy-5α-chloro-6,19-oxidoandrostan-17-one (**1**) ($CDCl_3$, 151 MHz)
Fig.S6. ^{1}H NMR spectral of 3β-acetyloxy-5α-chloro-6,19-oxidoandrostan-17-one (**1**) (DMSO-d_6, 600 MHz)
Fig.S7. ^{13}C NMR spectral of 3β-acetyloxy-5α-chloro-6,19-oxidoandrostan-17-one (**1**) (DMSO-d_6, 151 MHz)
Fig.S8. HMQC spectral of 3β-acetyloxy-5α-chloro-6,19-oxidoandrostan-17-one (**1**) (DMSO-d_6, 151 MHz)
Fig.S9. COSY spectral of 3β-acetyloxy-5α-chloro-6,19-oxidoandrostan-17-one (**1**) (DMSO-d_6, 151 MHz)
Fig.S10. HMBC spectral of 3β-acetyloxy-5α-chloro-6,19-oxidoandrostan-17-one (**1**) (DMSO-d_6, 151 MHz)
Fig.S11. GC-MS spectra of 3β-hydroxy-5α-chloro-6,19-oxidoandrostan-17-one (**2**)
Fig.S12. ^{1}H NMR spectral of 3β-hydroxy-5α-chloro-6,19-oxidoandrostan-17-one (**2**) ($CDCl_3$, 600 MHz)
Fig.S13. ^{13}C NMR spectral of 3β-hydroxy-5α-chloro-6,19-oxidoandrostan-17-one (**2**) ($CDCl_3$, 151 MHz)
Fig.S14. HMQC spectral of 3β-hydroxy-5α-chloro-6,19-oxidoandrostan-17-one (**2**) ($CDCl_3$, 151 MHz)
Fig.S15. HMBC spectral of 3β-hydroxy-5α-chloro-6,19-oxidoandrostan-17-one (**2**) ($CDCl_3$, 151 MHz)
Fig.S16. COSY spectral of 3β-hydroxy-5α-chloro-6,19-oxidoandrostan-17-one (**2**) ($CDCl_3$, 151 MHz)
Fig.S17. GC-MS spectra of 3β,17α-dihydroxy-5α-chloro-6,19-oxidoandrostan (**3**)
Fig.S18. ^{1}H NMR spectral of 3β,17α-dihydroxy-5α-chloro-6,19-oxidoandrostan (**3**) ($CDCl_3$, 600 MHz)
Fig.S19. ^{13}C NMR spectral of 3β,17α-dihydroxy-5α-chloro-6,19-oxidoandrostan (**3**) ($CDCl_3$, 151 MHz)
Fig.S20. HMQC spectral of 3β,17α-dihydroxy-5α-chloro-6,19-oxidoandrostan (**3**) ($CDCl_3$, 151 MHz)
Fig.S21. HMBC spectral of 3β,17α-dihydroxy-5α-chloro-6,19-oxidoandrostan (**3**) ($CDCl_3$, 151 MHz)
Fig.S22. COSY spectral of 3β,17α-dihydroxy-5α-chloro-6,19-oxidoandrostan (**3**) ($CDCl_3$, 151 MHz)
Fig.S23. ^{1}H NMR spectral of 3β,17α-dihydroxy-5α-chloro-6,19-oxidoandrostan (**3**) (DMSO-d_6, 600 MHz)
Fig.S24. ^{13}C NMR spectral of 3β,17α-dihydroxy-5α-chloro-6,19-oxidoandrostan (**3**) (DMSO-d_6, 151 MHz)
Fig.S25. HMQC spectral of 3β,17α-dihydroxy-5α-chloro-6,19-oxidoandrostan (**3**) (DMSO-d_6, 151 MHz)
Fig.S26. HMBC spectral of 3β,17α-dihydroxy-5α-chloro-6,19-oxidoandrostan (**3**) (DMSO-d_6, 151 MHz)
Fig.S27. COSY spectral of 3β,17α-dihydroxy-5α-chloro-6,19-oxidoandrostan (**3**) (DMSO-d_6, 151 MHz)
Fig.S28. GC-MS spectra of 3β-hydroxy-5α-chloro-17a-oxa-D-homo-6,19-oxidoandrostan-17-one (**4**)
Fig.S29. ^{1}H NMR spectral of 3β-hydroxy-5α-chloro-17a-oxa-D-homo-6,19-oxidoandrostan-17-one (**4**) ($CDCl_3$, 600 MHz)
Fig.S30. ^{13}C NMR spectral of 3β-hydroxy-5α-chloro-17a-oxa-D-homo-6,19-oxidoandrostan-17-one (**4**) ($CDCl_3$, 151 MHz)
Fig.S31. HMQC spectral of 3β-hydroxy-5α-chloro-17a-oxa-D-homo-6,19-oxidoandrostan-17-one (**4**) ($CDCl_3$, 151 MHz)

Fig.S32. HMBC spectral of 3β-hydroxy-5α-chloro-17a-oxa-D-homo-6,19-oxidoandrostan-17-one (**4**) ($CDCl_3$, 151 MHz)
Fig.S33. COSY spectral of 3β-hydroxy-5α-chloro-17a-oxa-D-homo-6,19-oxidoandrostan-17-one (**4**) ($CDCl_3$, 151 MHz)
Fig.S34. GC-MS spectra of 3β,11α-dihydroxy-5α-chloro-17a-oxa-D-homo-6,19-oxidoandrostan-17-one (**5**)
Fig.S35. ^{1}H NMR spectral of 3β,11α-dihydroxy-5α-chloro-17a-oxa-D-homo-6,19-oxidoandrostan-17-one (**5**) ($CDCl_3$, 600 MHz)
Fig.S36. ^{13}C NMR spectral of 3β,11α-dihydroxy-5α-chloro-17a-oxa-D-homo-6,19-oxidoandrostan-17-one (**5**) ($CDCl_3$, 151 MHz)
Fig.S37. HMQC spectral of 3β,11α-dihydroxy-5α-chloro-17a-oxa-D-homo-6,19-oxidoandrostan-17-one (**5**) ($CDCl_3$, 151 MHz)
Fig.S38. HMBC spectral of 3β,11α-dihydroxy-5α-chloro-17a-oxa-D-homo-6,19-oxidoandrostan-17-one (**5**) ($CDCl_3$, 151 MHz)
Fig.S39. COSY spectral of 3β,11α-dihydroxy-5α-chloro-17a-oxa-D-homo-6,19-oxidoandrostan-17-one (**5**) ($CDCl_3$, 151 MHz)
Fig.S40. GC-MS spectra of 3β,11α-dihydroxy-5α-chloro-6,19-oxidoandrostan-17-one (**6**)
Fig.S41. ^{1}H NMR spectral of 3β,11α-dihydroxy-5α-chloro-6,19-oxidoandrostan-17-one (**6**) ($CDCl_3$, 600 MHz)
Fig.S42. ^{13}C NMR spectral of 3β,11α-dihydroxy-5α-chloro-6,19-oxidoandrostan-17-one (**6**) ($CDCl_3$, 151 MHz)
Fig.S43. HMQC spectral of 3β,11α-dihydroxy-5α-chloro-6,19-oxidoandrostan-17-one (**6**) ($CDCl_3$, 151 MHz)
Fig.S44. HMBC spectral of 3β,11α-dihydroxy-5α-chloro-6,19-oxidoandrostan-17-one (**6**) ($CDCl_3$, 151 MHz)
Fig.S45. COSY spectral of 3β,11α-dihydroxy-5α-chloro-6,19-oxidoandrostan-17-one (**6**) ($CDCl_3$, 151 MHz)
Fig.S46. GC-MS spectra of 3β,11α,19-trihydroxy-5α-chloro-6,19-oxidoandrostan-17-one (**7**)
Fig.S47. ^{1}H NMR spectral of 3β,11α,19-trihydroxy-5α-chloro-6,19-oxidoandrostan-17-one (**7**) ($CDCl_3$, 600 MHz)
Fig.S48. ^{13}C NMR spectral of 3β,11α,19-trihydroxy-5α-chloro-6,19-oxidoandrostan-17-one (**7**) ($CDCl_3$, 151 MHz)
Fig.S49. HMQC spectral of 3β,11α,19-trihydroxy-5α-chloro-6,19-oxidoandrostan-17-one (**7**) ($CDCl_3$, 151 MHz)
Fig.S50. HMBC spectral of 3β,11α,19-trihydroxy-5α-chloro-6,19-oxidoandrostan-17-one (**7**) ($CDCl_3$, 151 MHz)
Fig.S51. COSY spectral of 3β,11α,19-trihydroxy-5α-chloro-6,19-oxidoandrostan-17-one (**7**) ($CDCl_3$, 151 MHz)
Fig.S52. GC-MS spectra of 3β,11α,19-trihydroxy-5α-chloro-6,19-oxidoandrostan-17-one (**7**)
Fig.S53. ^{1}H NMR spectral of 3β,11α-dihydroxy-5α-chloro-6,19-oxidoandrostan-17-one (**6**) and 3β,11α-dihydroxy-5α-chloro-6,19-oxidoandrostan-17,19-dione (**8**) (DMSO-d_6, 600 MHz)
Fig.S54. ^{13}C NMR spectral of 3β,11α-dihydroxy-5α-chloro-6,19-oxidoandrostan-17-one (**6**) and 3β,11α-dihydroxy-5α-chloro-6,19-oxidoandrostan-17,19-dione (**8**) (DMSO-d_6, 151 MHz). Signals from carbons of compound **8** were assigned.
Fig.S55. ^{13}C NMR spectral of 3β,11α-dihydroxy-5α-chloro-6,19-oxidoandrostan-17-one (**6**) and 3β,11α-dihydroxy-5α-chloro-6,19-oxidoandrostan-17,19-dione (**8**) (DMSO-d_6, 151 MHz). Signals from carbons of compound **6** were assigned.
Fig.S56. HMQC spectral of 3β,11α-dihydroxy-5α-chloro-6,19-oxidoandrostan-17-one (**6**) and 3β,11α-dihydroxy-5α-chloro-6,19-oxidoandrostan-17,19-dione (**8**) (DMSO-d_6, 151 MHz)
Fig.S57. COSY spectral of 3β,11α-dihydroxy-5α-chloro-6,19-oxidoandrostan-17-one (**6**) and 3β,11α-dihydroxy-5α-chloro-6,19-oxidoandrostan-17,19-dione (**8**) (DMSO-d_6, 151 MHz)

Fig.S1. GC-MS spectra of 3β-acetyloxy-5α-chloro-6,19-oxidoandrostan-17-one (**1**)

Molecular Formula = $C_{21}H_{29}ClO_4$
Formula Weight = 380.90556

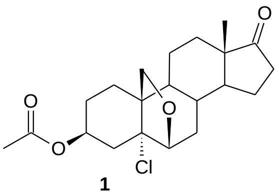

1

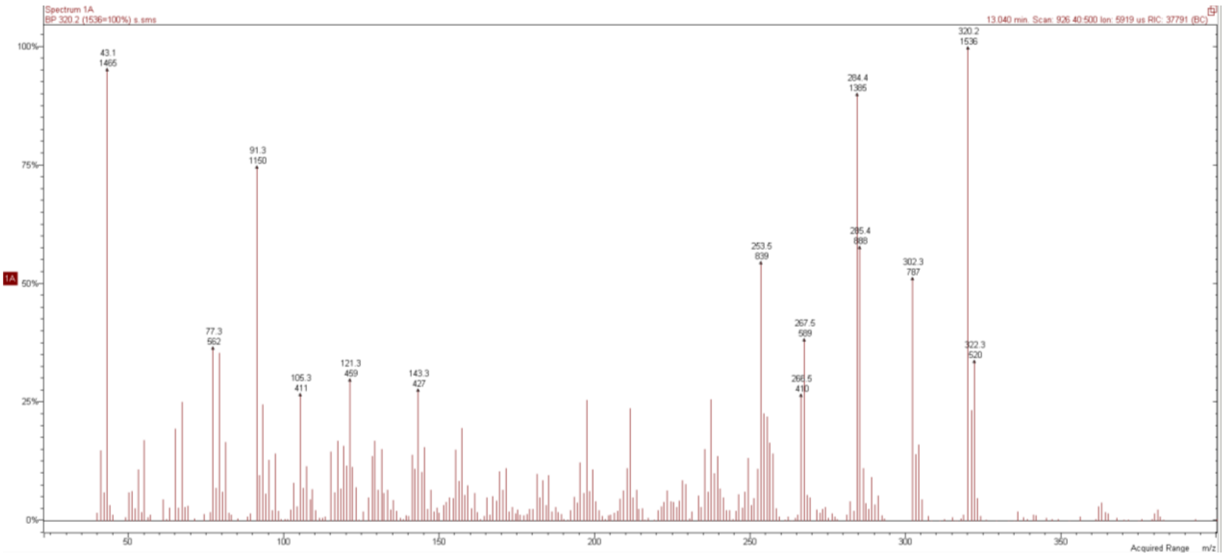

Fig.S2. ^{1}H NMR spectral of 3β-acetyloxy-5α-chloro-6,19-oxidoandrostan-17-one (**1**) ($CDCl_3$, 600 MHz)

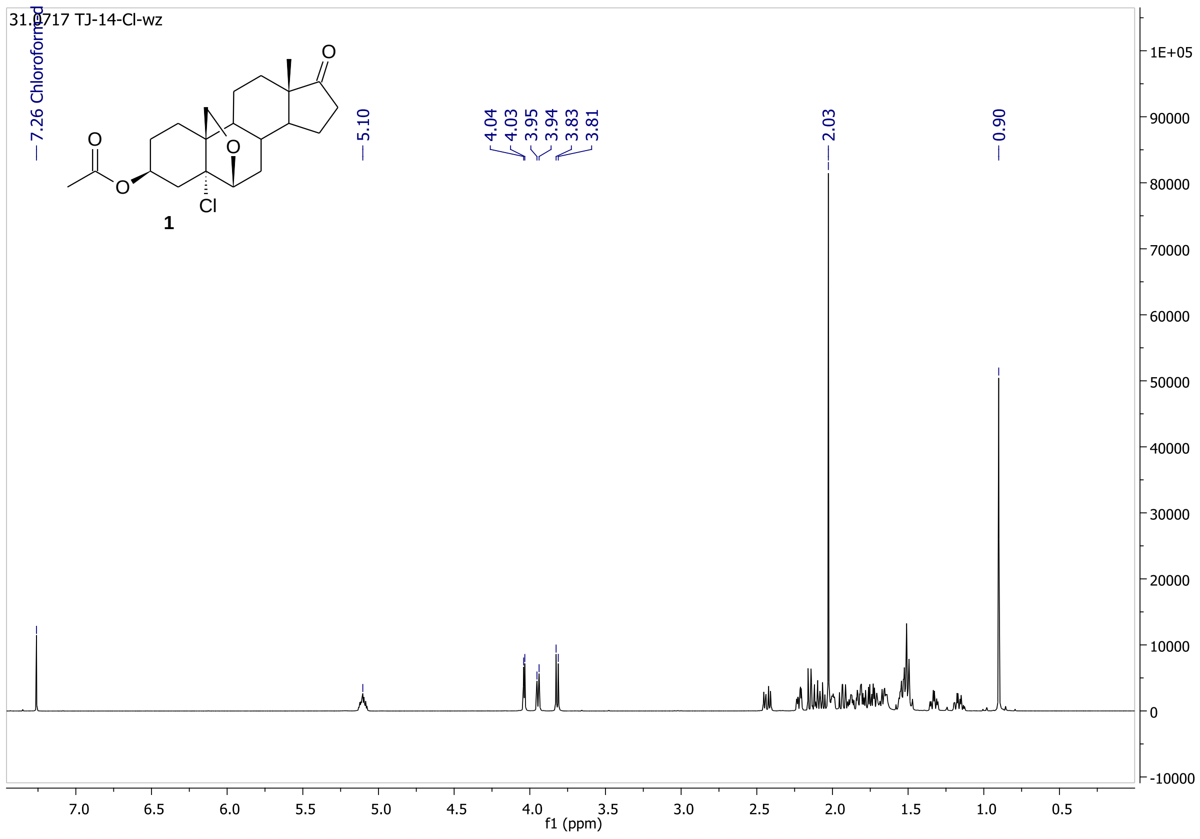

Fig.S3. ^{13}C NMR spectral of 3β-acetyloxy-5α-chloro-6,19-oxidoandrostan-17-one (**1**) ($CDCl_3$, 151 MHz)

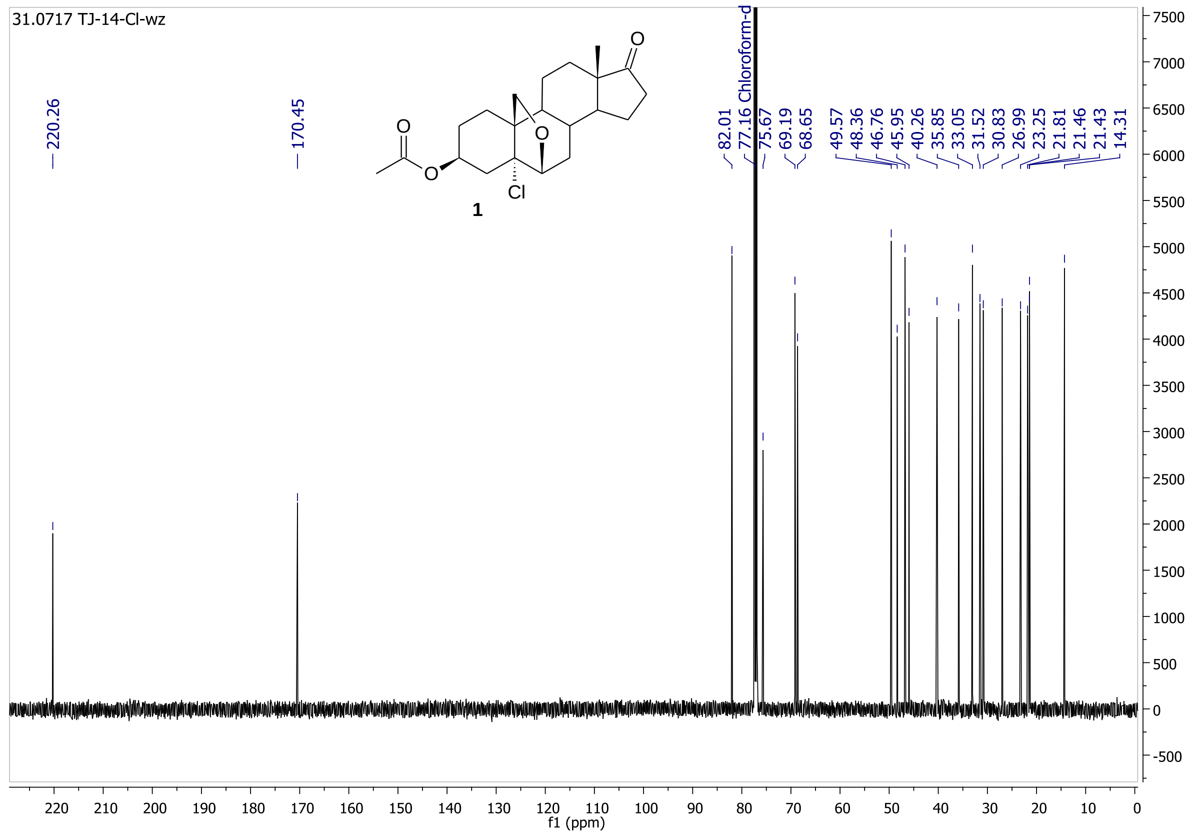

Fig.S4. HSQC spectral of 3β-acetyloxy-5α-chloro-6,19-oxidoandrostan-17-one (**1**) ($CDCl_3$, 151 MHz)

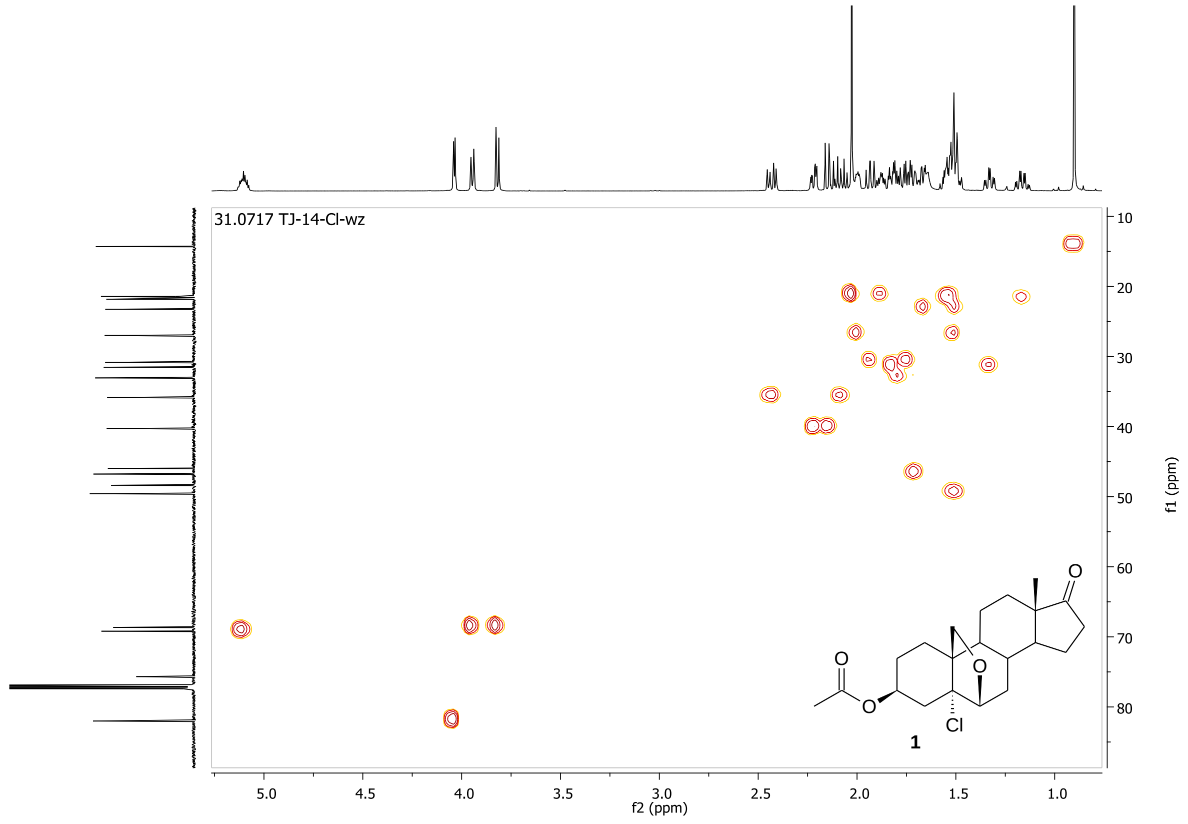

Fig.S5. COSY spectral of 3β-acetyloxy-5α-chloro-6,19-oxidoandrostan-17-one (**1**) ($CDCl_3$, 151 MHz)

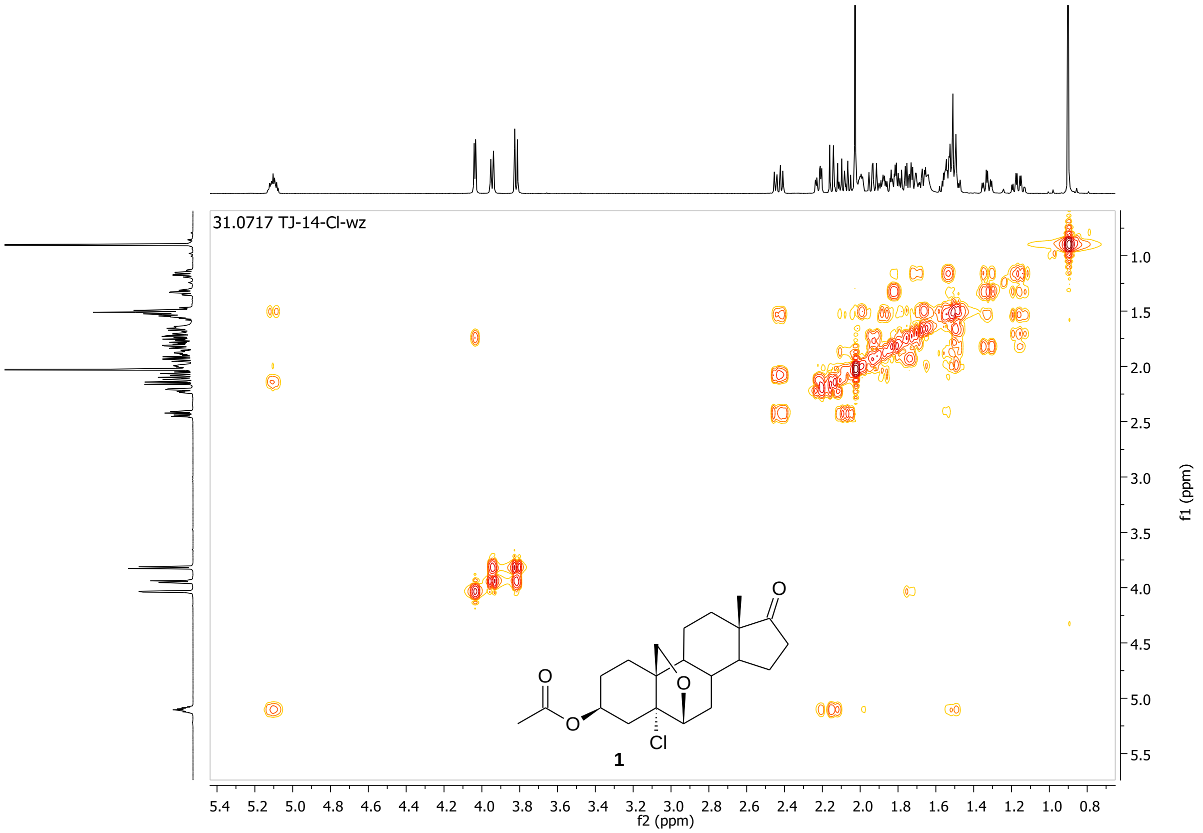

Fig.S6. ^{1}H NMR spectral of 3β-acetyloxy-5α-chloro-6,19-oxidoandrostan-17-one (**1**) (DMSO-d_6, 600 MHz)

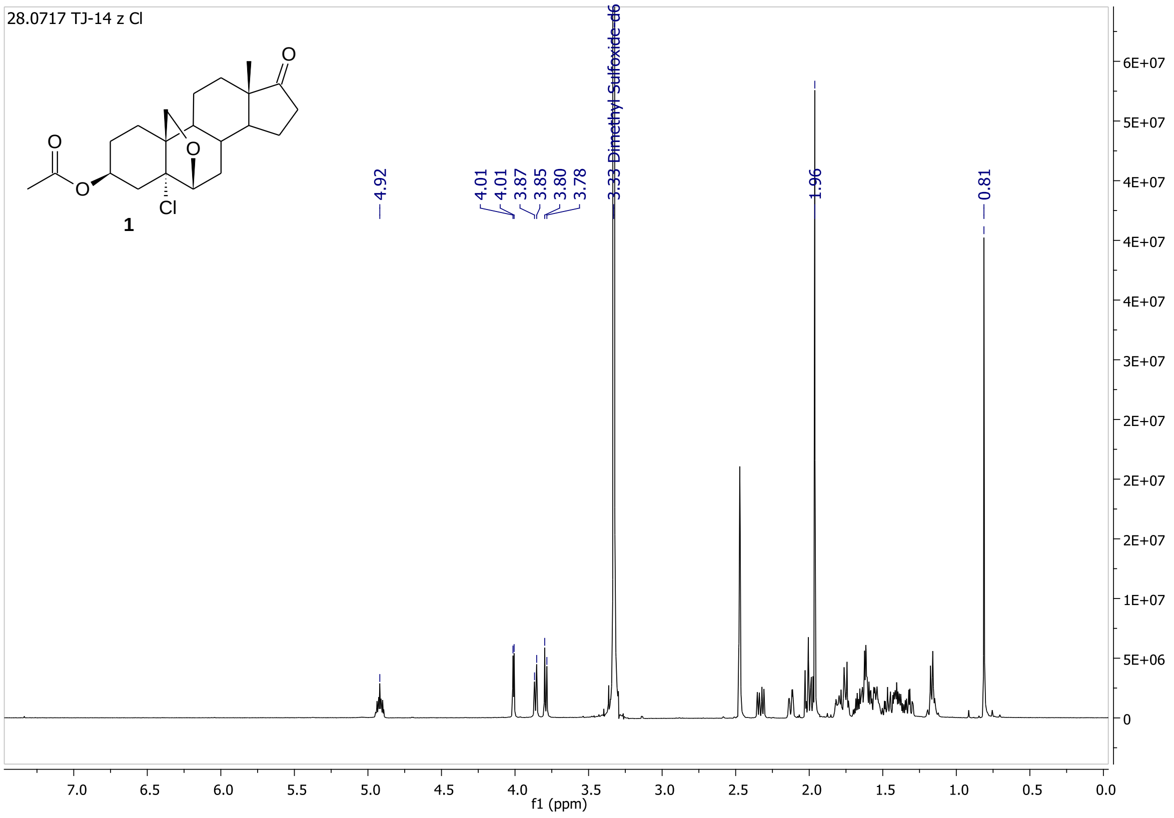

Fig.S7. ^{13}C NMR spectral of 3β-acetyloxy-5α-chloro-6,19-oxidoandrostan-17-one (**1**) (DMSO-d_6, 151 MHz)

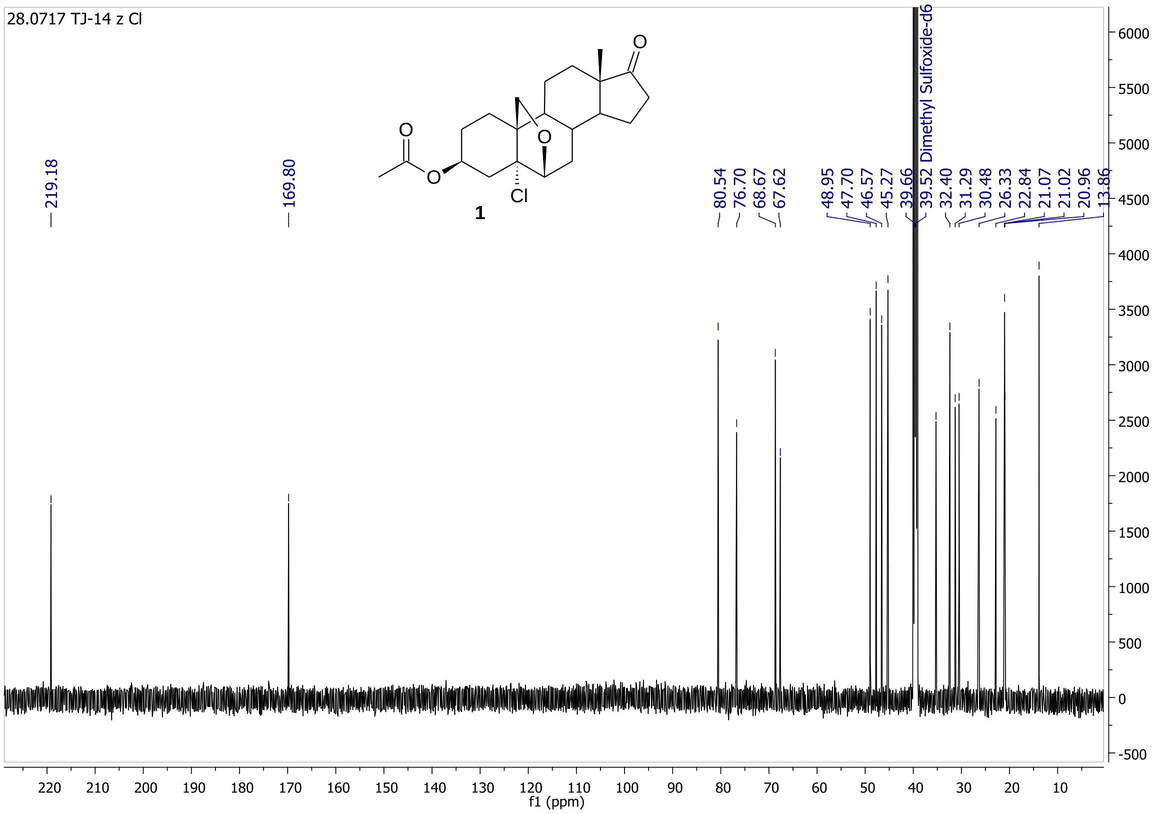

Fig.S8. HMQC spectral of 3β-acetyloxy-5α-chloro-6,19-oxidoandrostan-17-one (**1**) (DMSO-d_6, 151 MHz)

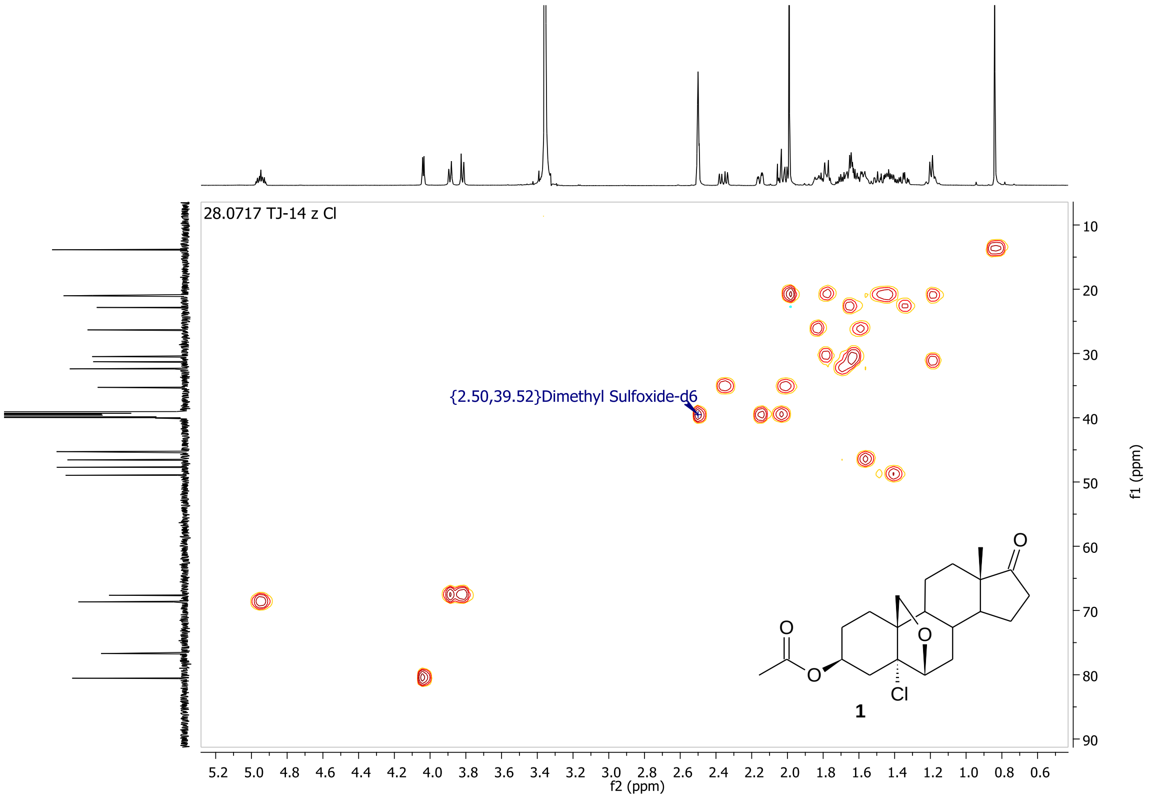

Fig.S9. COSY spectral of 3β-acetyloxy-5α-chloro-6,19-oxidoandrostan-17-one (**1**) (DMSO-d_6, 151 MHz)

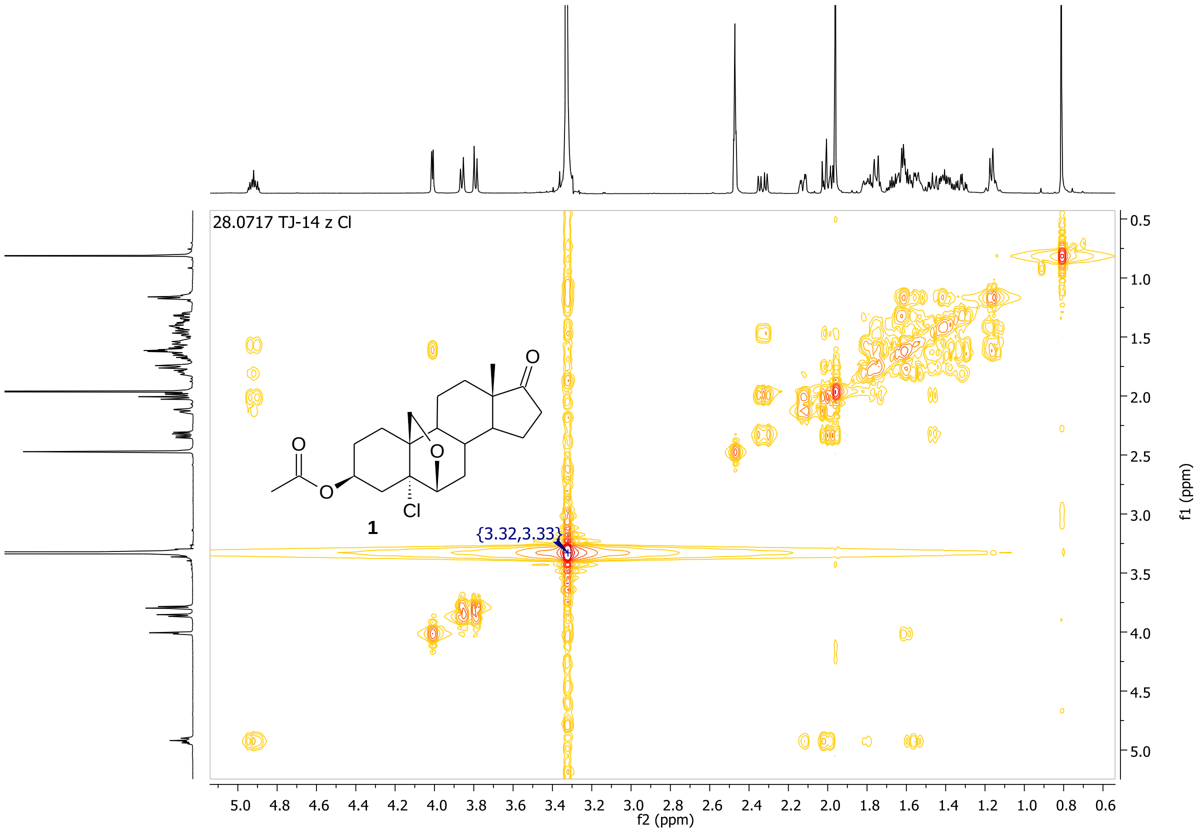

Fig.S10. HMBC spectral of 3β-acetyloxy-5α-chloro-6,19-oxidoandrostan-17-one (**1**) (DMSO-d_6, 151 MHz)

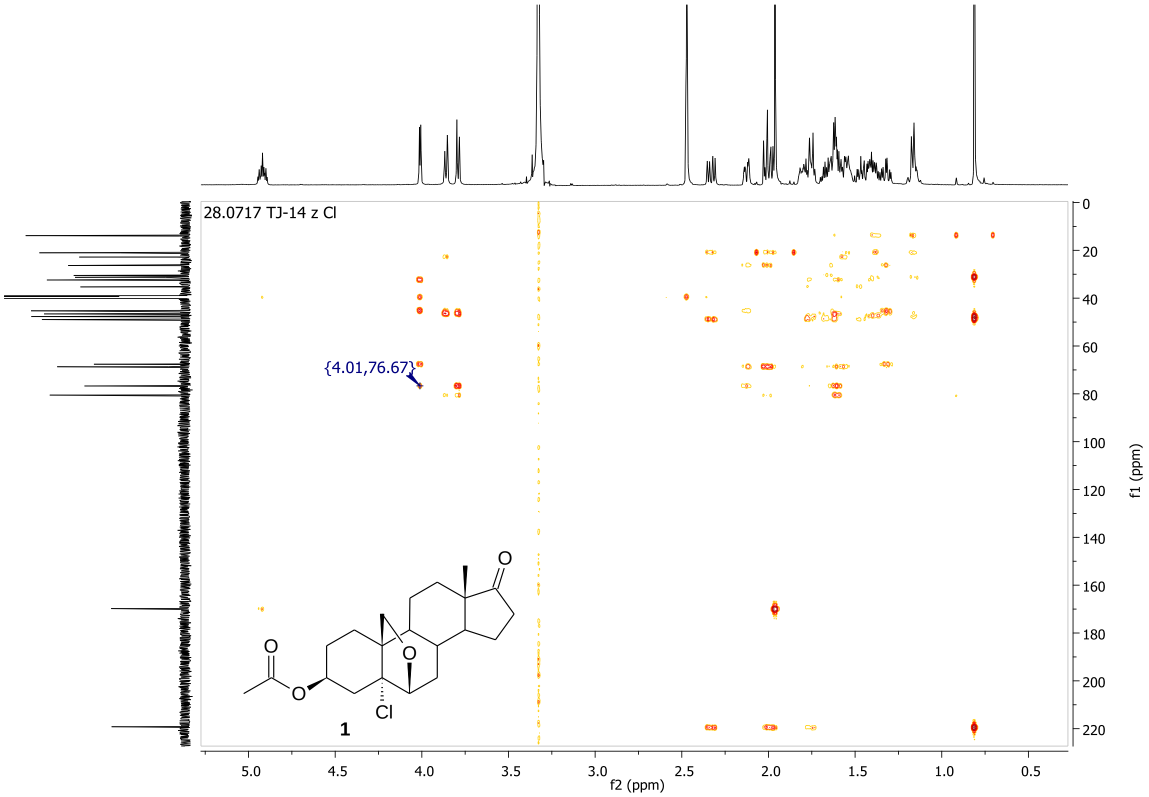

Fig.S11. GC-MS spectra of 3β-hydroxy-5α-chloro-6,19-oxidoandrostan-17-one (**2**)

Molecular Formula = $C_{19}H_{27}ClO_3$
Formula Weight = 338.86888

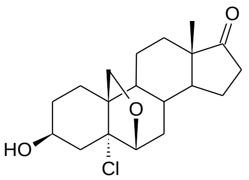

2

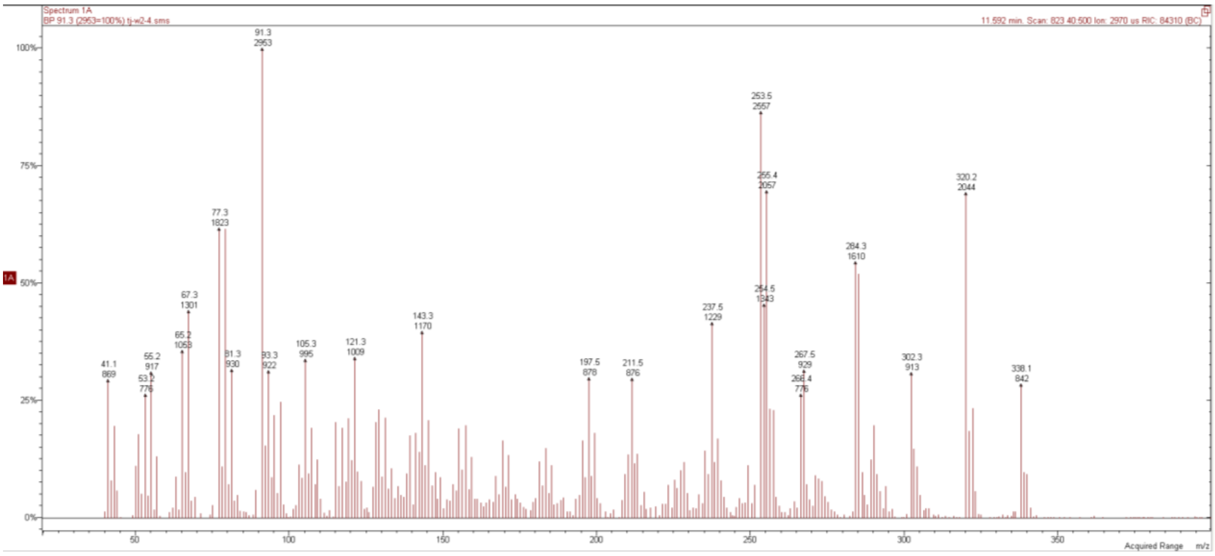

Fig.S12. ^{1}H NMR spectral of 3β-hydroxy-5α-chloro-6,19-oxidoandrostan-17-one (**2**) ($CDCl_3$, 600 MHz)

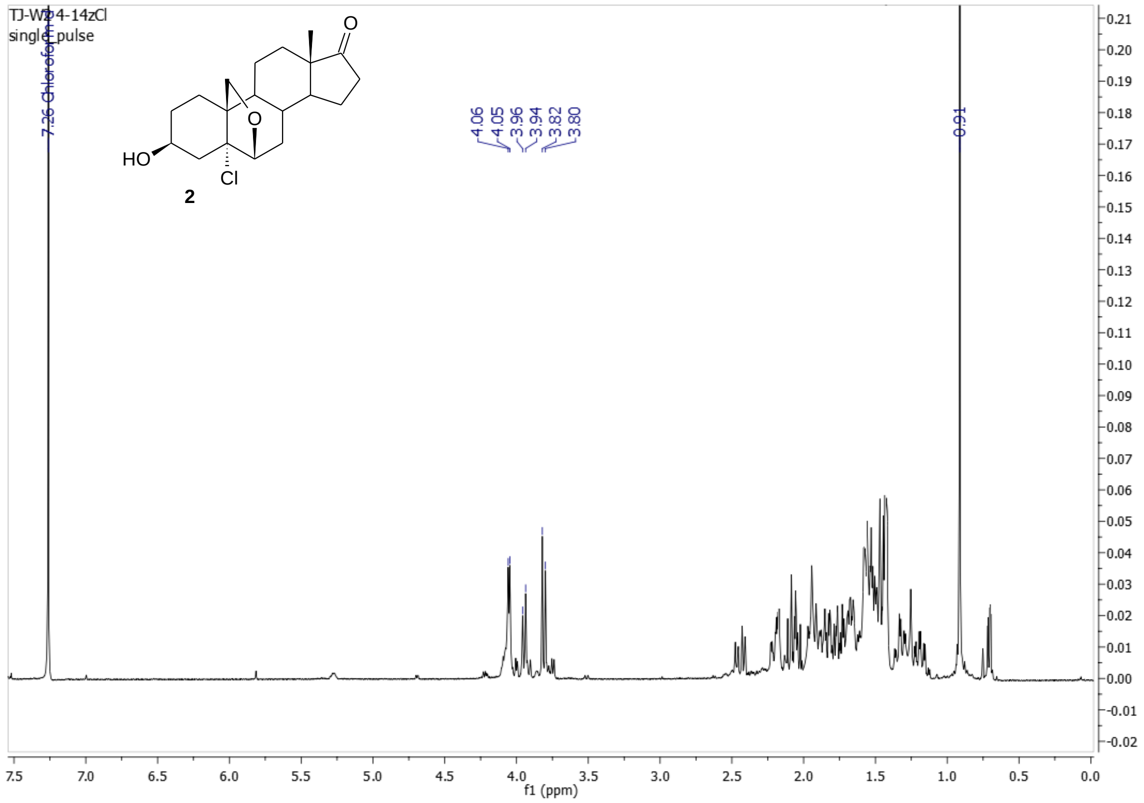

Fig.S13. ^{13}C NMR spectral of 3β-hydroxy-5α-chloro-6,19-oxidoandrostan-17-one (**2**) ($CDCl_3$, 151 MHz)

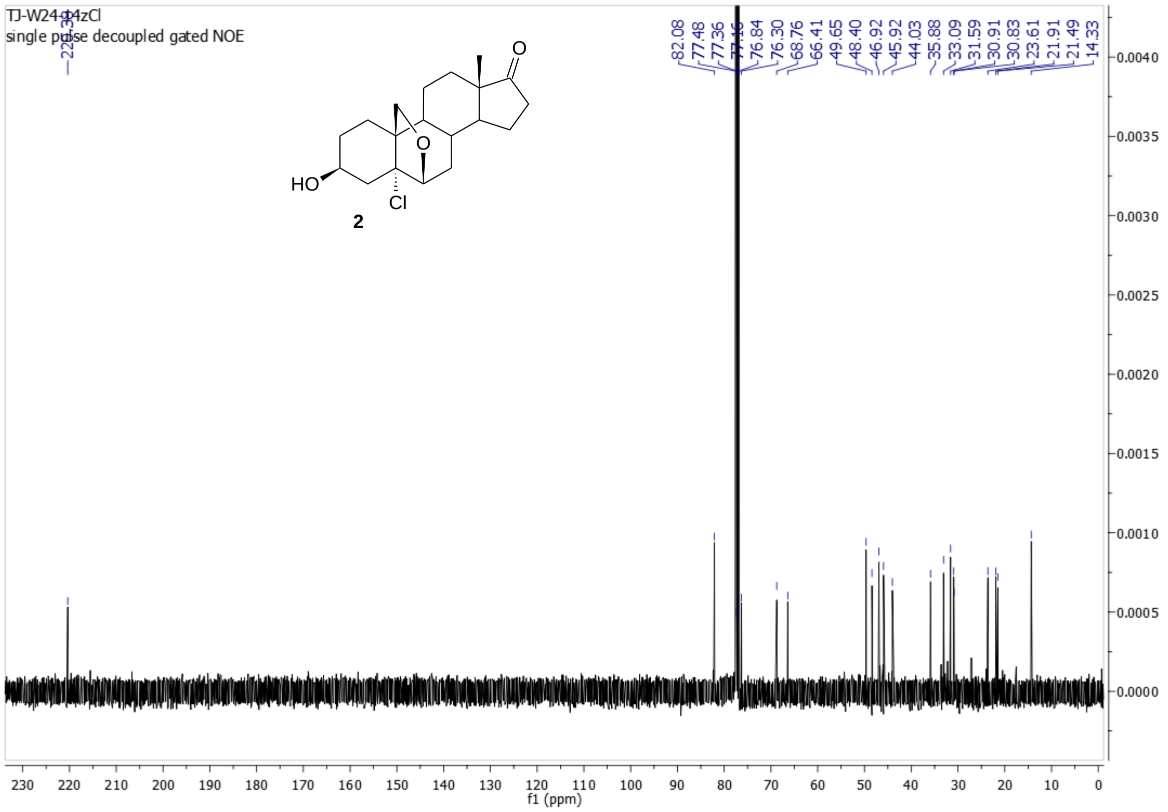

Fig.S14. HMQC spectral of 3β-hydroxy-5α-chloro-6,19-oxidoandrostan-17-one (**2**) ($CDCl_3$, 151 MHz)

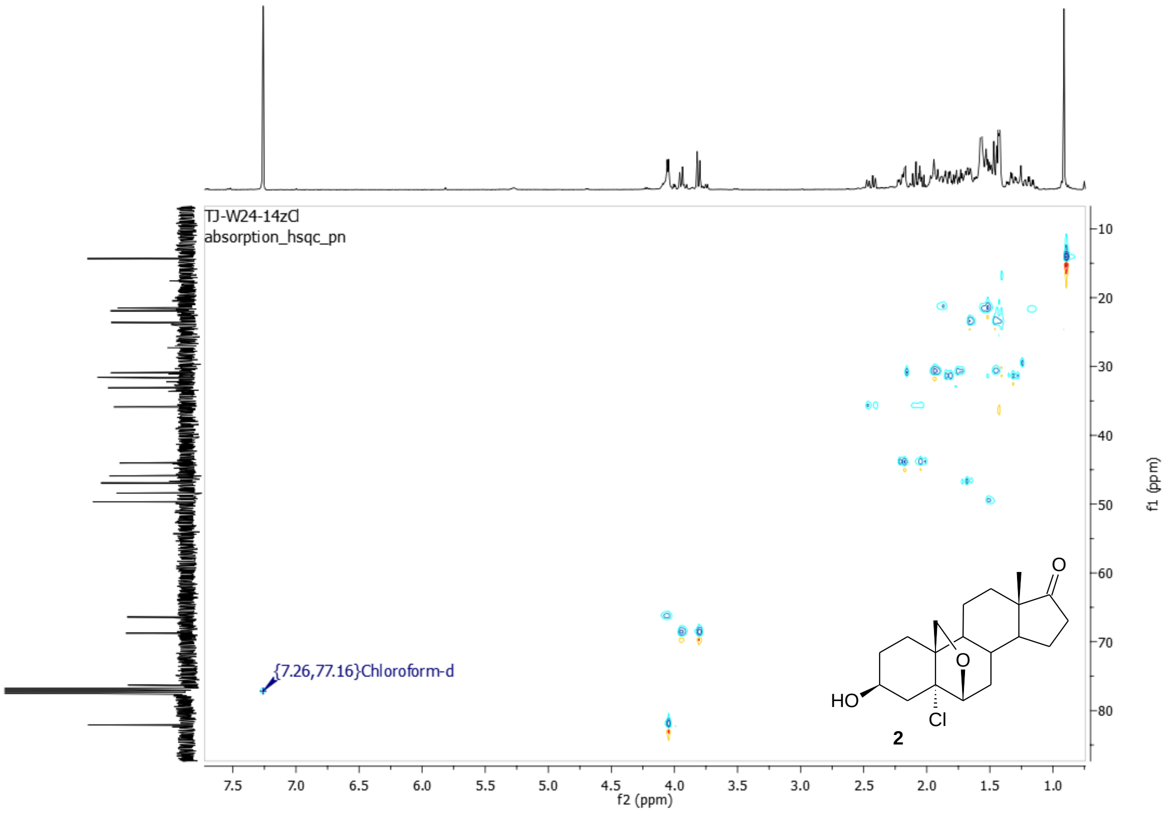

Fig.S15. HMBC spectral of 3β-hydroxy-5α-chloro-6,19-oxidoandrostan-17-one (**2**) ($CDCl_3$, 151 MHz)

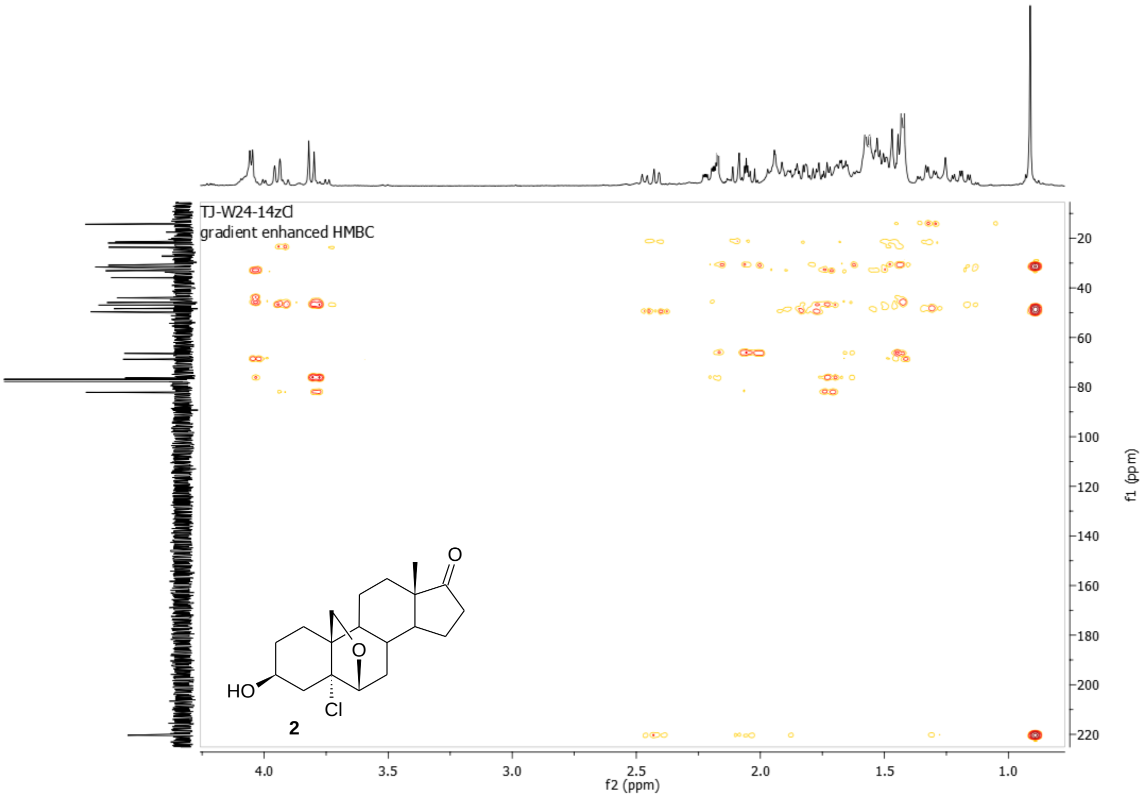

Fig.S16. COSY spectral of 3β-hydroxy-5α-chloro-6,19-oxidoandrostan-17-one (**2**) ($CDCl_3$, 151 MHz)

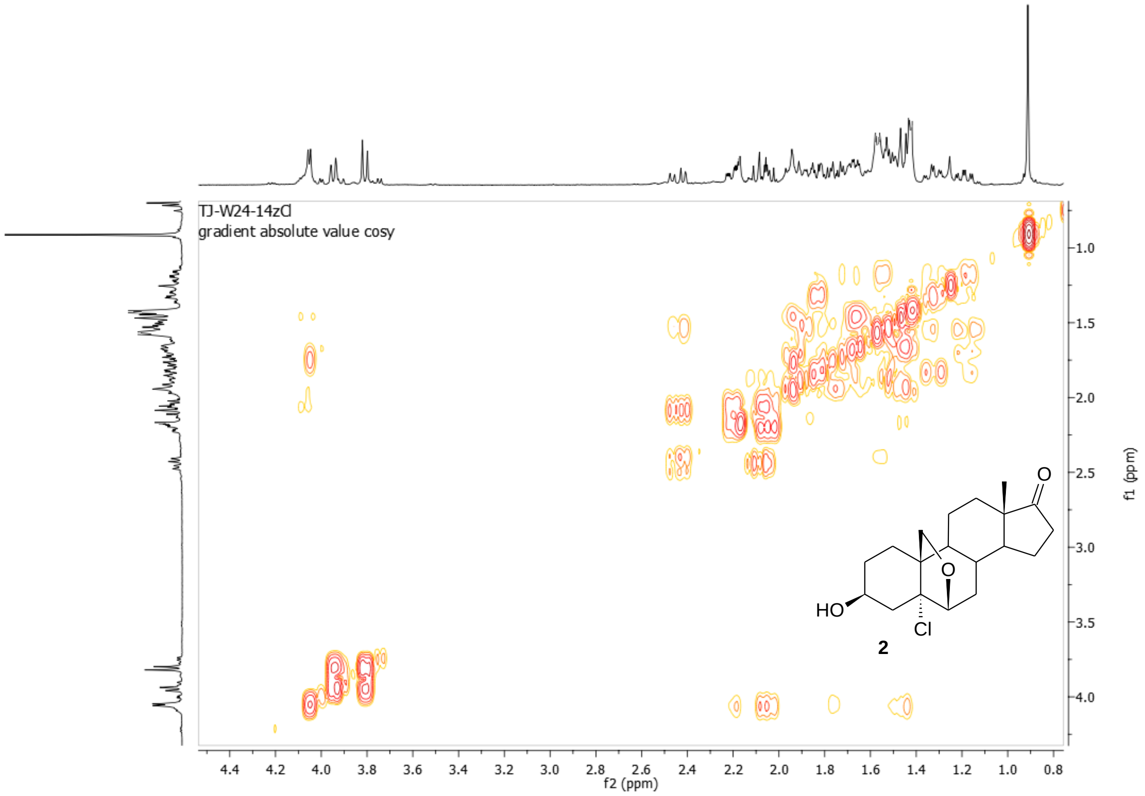

Fig.S17. GC-MS spectra of 3β,17α-dihydroxy-5α-chloro-6,19-oxidoandrostan (**3**)

Molecular Formula = $C_{19}H_{29}ClO_3$
Formula Weight = 340.88476

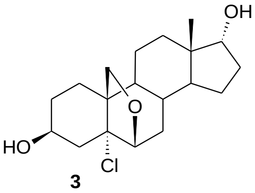

3

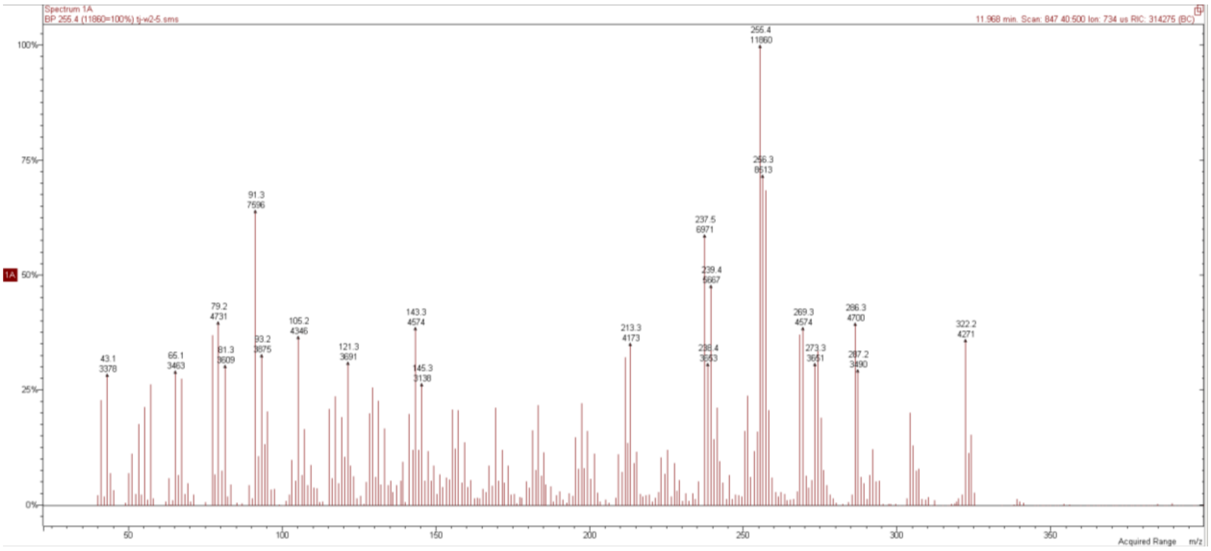

Fig.S18. ¹H NMR spectral of 3β,17α-dihydroxy-5α-chloro-6,19-oxidoandrostan (**3**) ($CDCl_3$, 600 MHz)

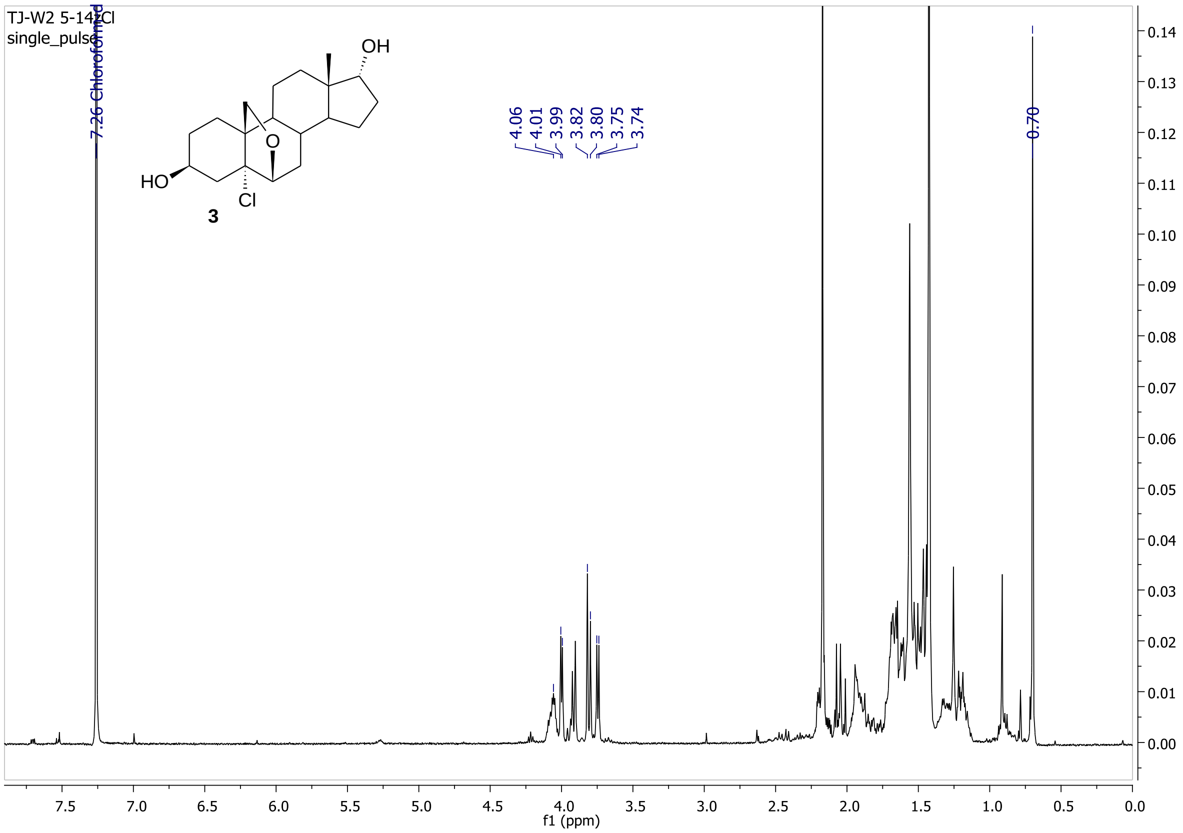

Fig.S19. ^{13}C NMR spectral of 3β,17α-dihydroxy-5α-chloro-6,19-oxidoandrostan (**3**) ($CDCl_3$, 151 MHz)

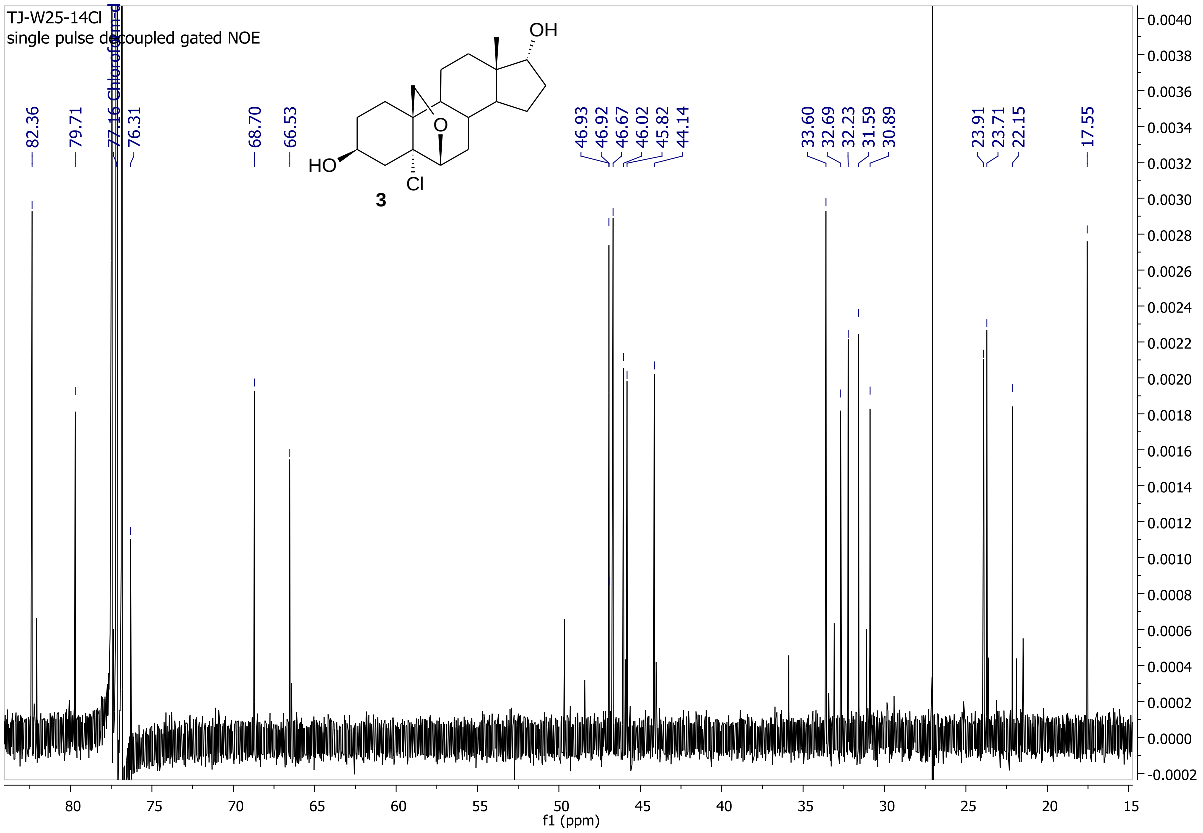

Fig.S20. HMQC spectral of 3β,17α-dihydroxy-5α-chloro-6,19-oxidoandrostan (**3**) ($CDCl_3$, 151 MHz)

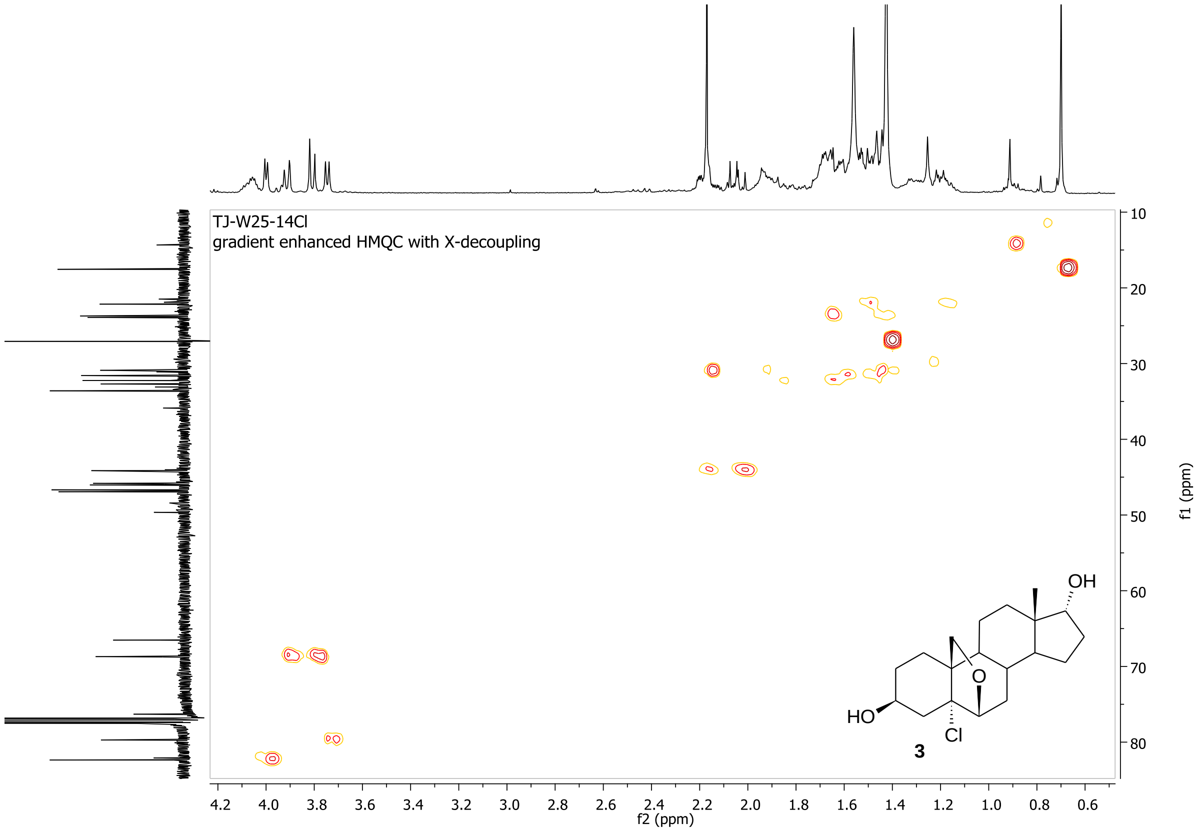

Fig.S21. HMBC spectral of 3β,17α-dihydroxy-5α-chloro-6,19-oxidoandrostan (**3**) ($CDCl_3$, 151 MHz)

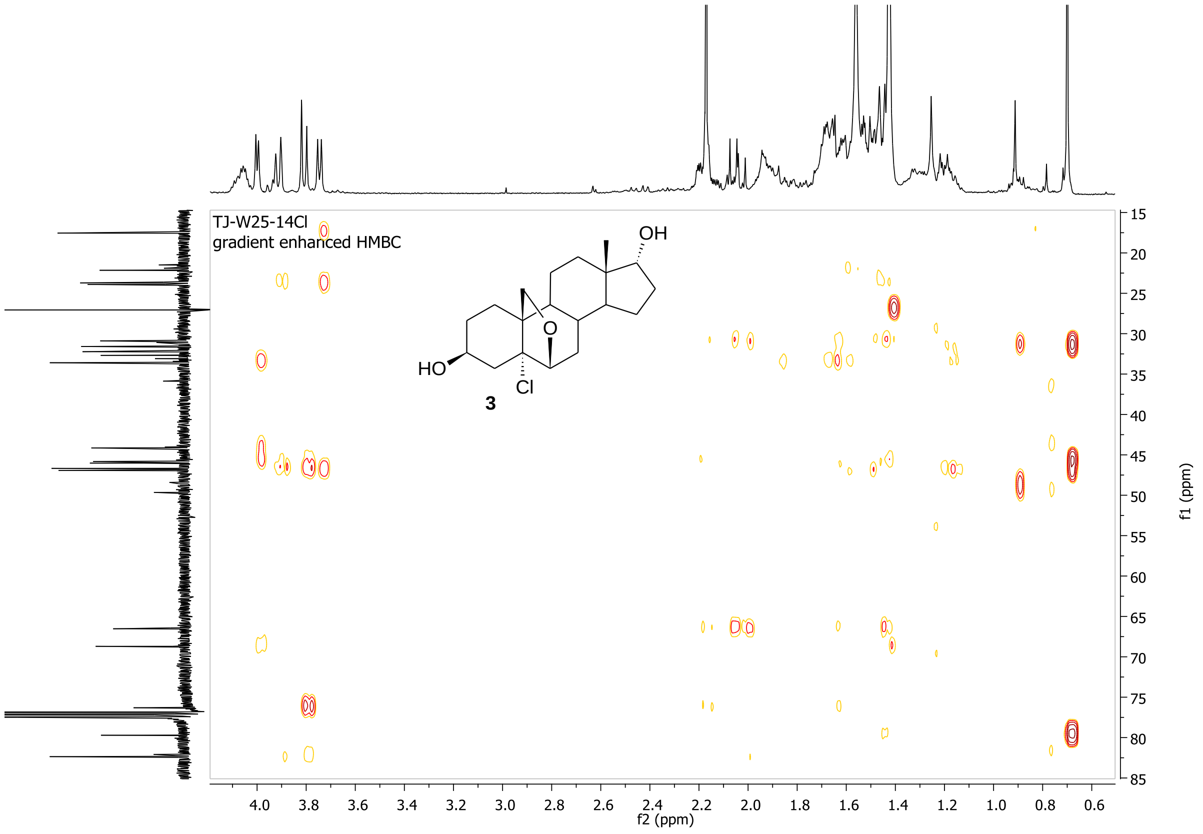

Fig.S22. COSY spectral of 3β,17α-dihydroxy-5α-chloro-6,19-oxidoandrostan (**3**) ($CDCl_3$, 151 MHz)

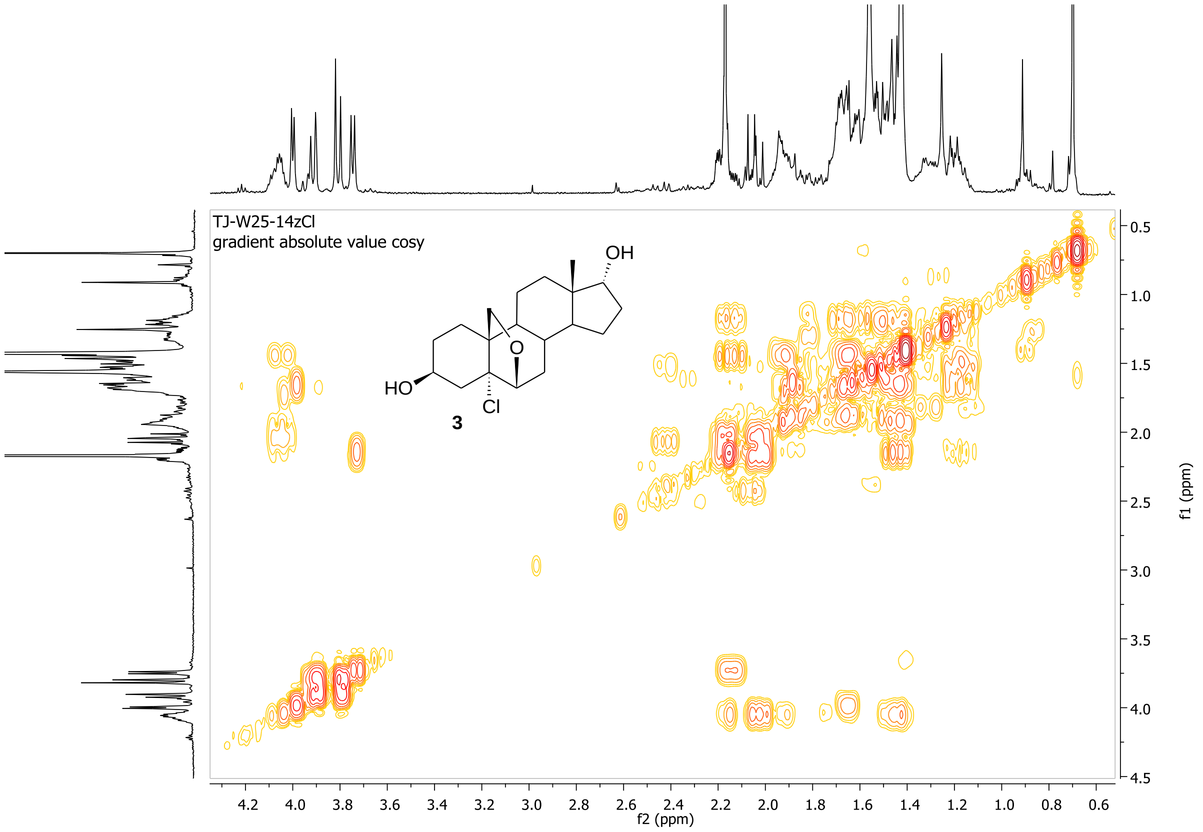

Fig.S23. ^{1}H NMR spectral of 3β,17α-dihydroxy-5α-chloro-6,19-oxidoandrostan (**3**) (DMSO-d_6, 600 MHz)

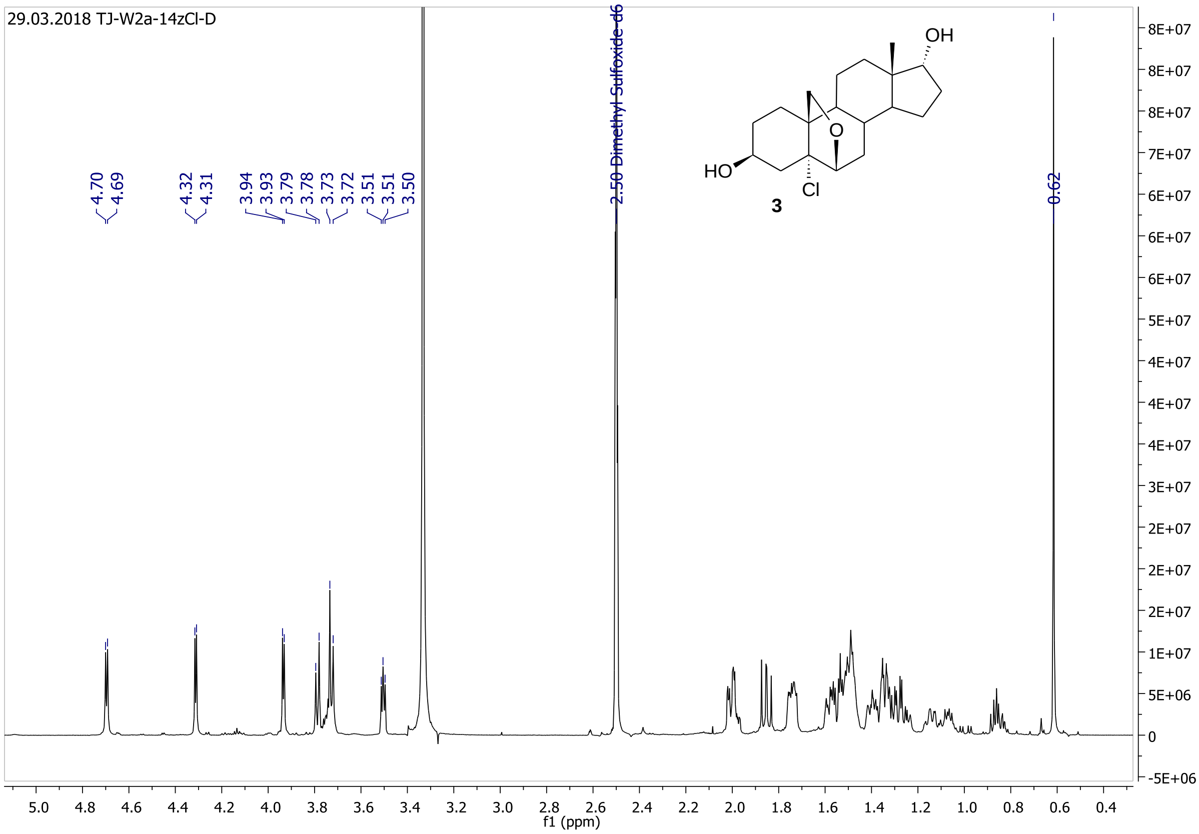

Fig.S24. ^{13}C NMR spectral of 3β,17α-dihydroxy-5α-chloro-6,19-oxidoandrostan (**3**) (DMSO-d_6, 151 MHz)

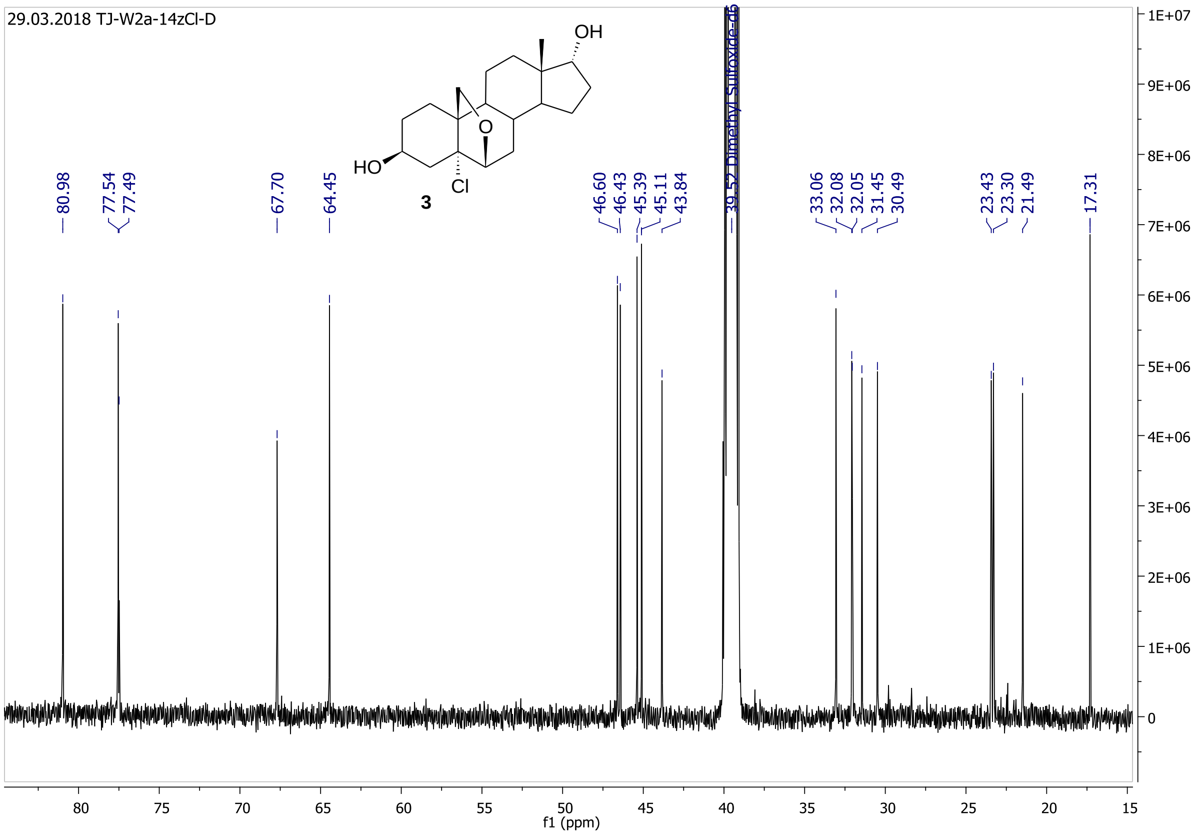

Fig.S25. HMQC spectral of 3β,17α-dihydroxy-5α-chloro-6,19-oxidoandrostan (**3**) (DMSO-d_6, 151 MHz)

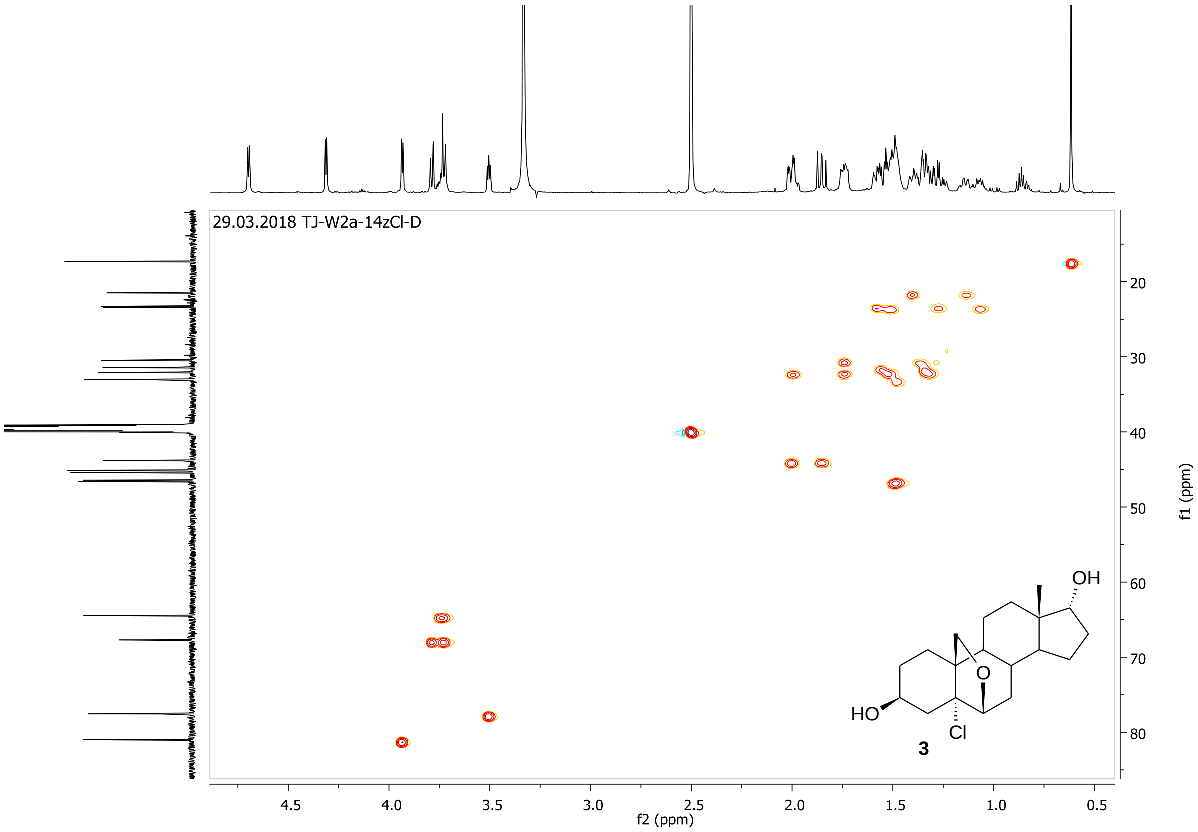

Fig.S26. HMBC spectral of 3β,17α-dihydroxy-5α-chloro-6,19-oxidoandrostan (**3**) (DMSO-d_6, 151 MHz)

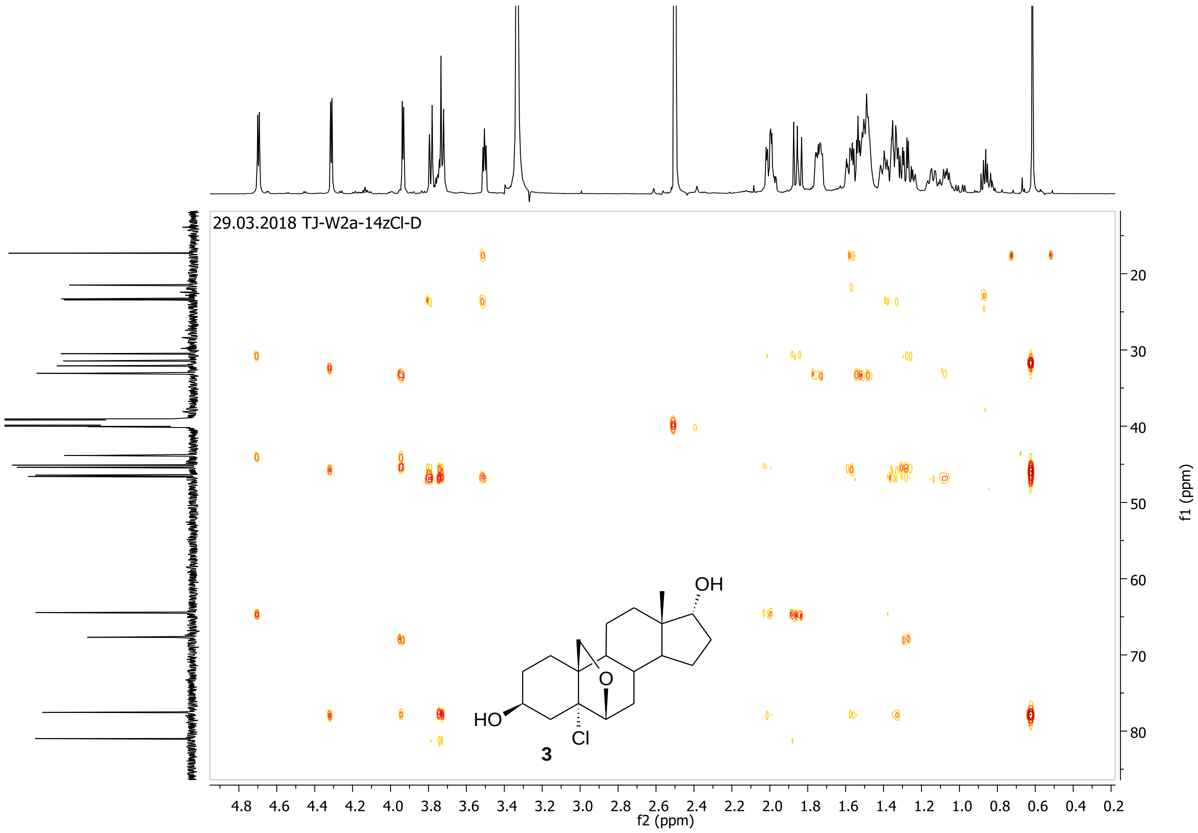

Fig.S27. COSY spectral of 3β,17α-dihydroxy-5α-chloro-6,19-oxidoandrostan (**3**) (DMSO-d_6, 151 MHz)

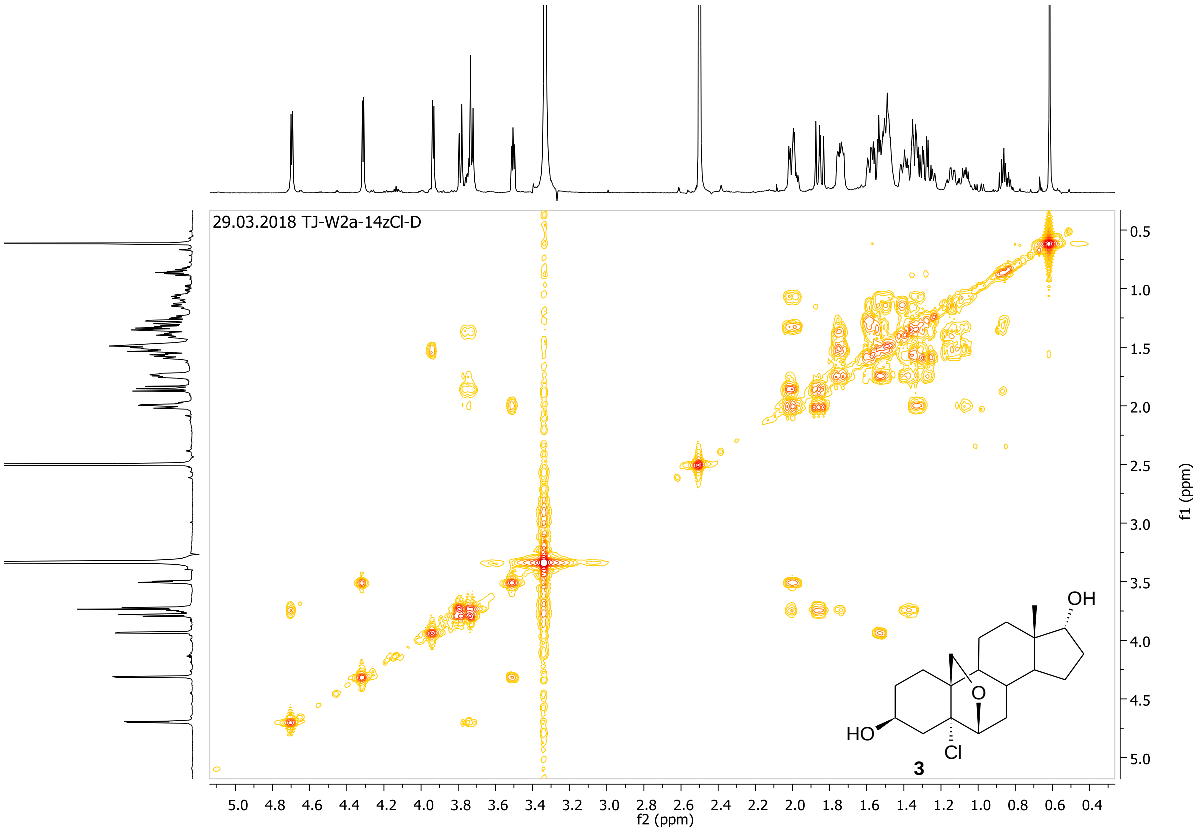

Fig.S28. GC-MS spectra of 3β-hydroxy-5α-chloro-17a-oxa-D-homo-6,19-oxidoandrostan-17-one (**4**)

Molecular Formula = $C_{19}H_{27}ClO_4$
Formula Weight = 354.86828

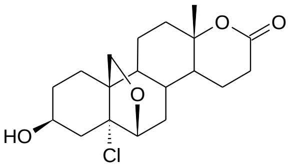

4

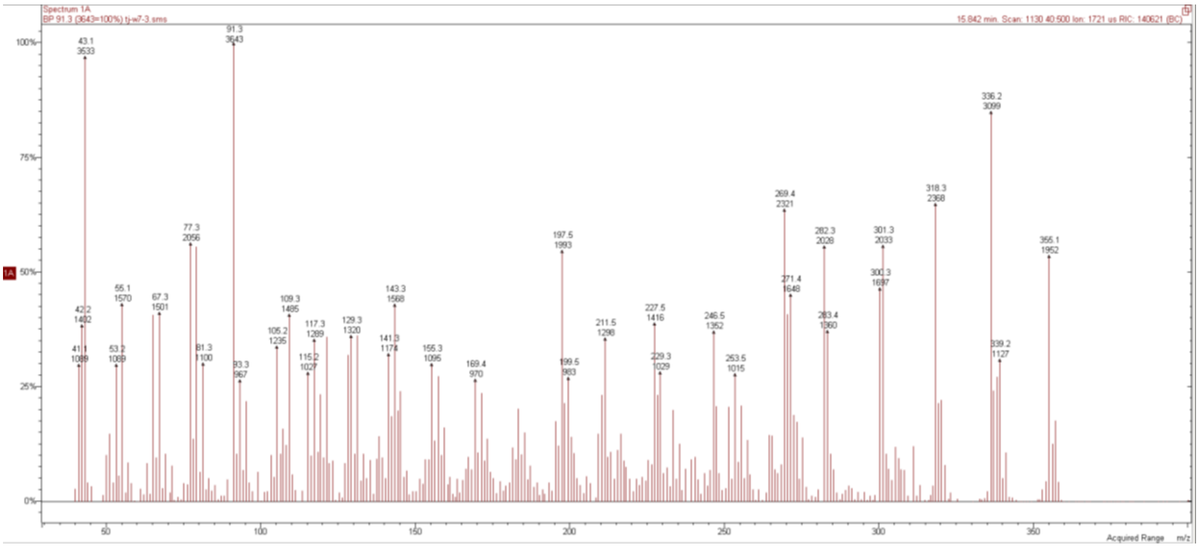

Fig.S29. ^{1}H NMR spectral of 3β-hydroxy-5α-chloro-17a-oxa-D-homo-6,19-oxidoandrostan-17-one (**4**) ($CDCl_3$, 600 MHz)

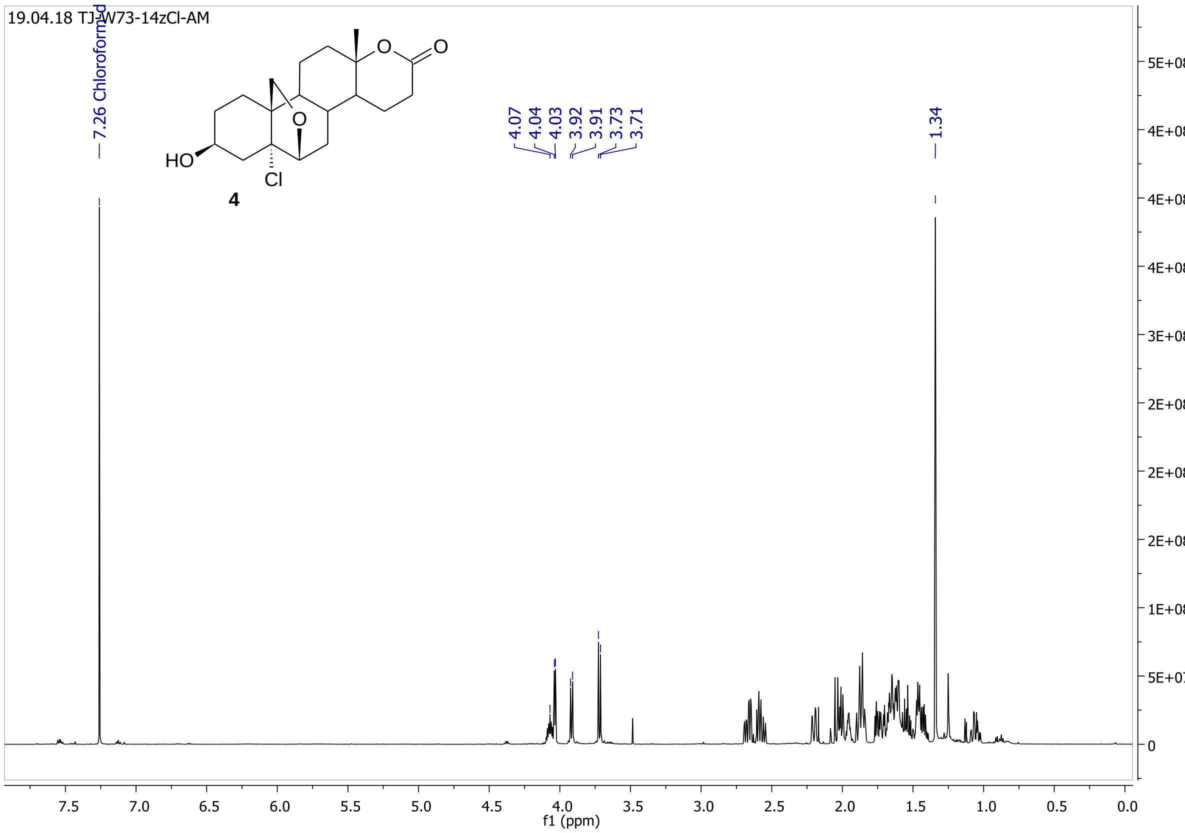

Fig.S30. ^{13}C NMR spectral of 3β-hydroxy-5α-chloro-17a-oxa-D-homo-6,19-oxidoandrostan-17-one (**4**) ($CDCl_3$, 151 MHz)

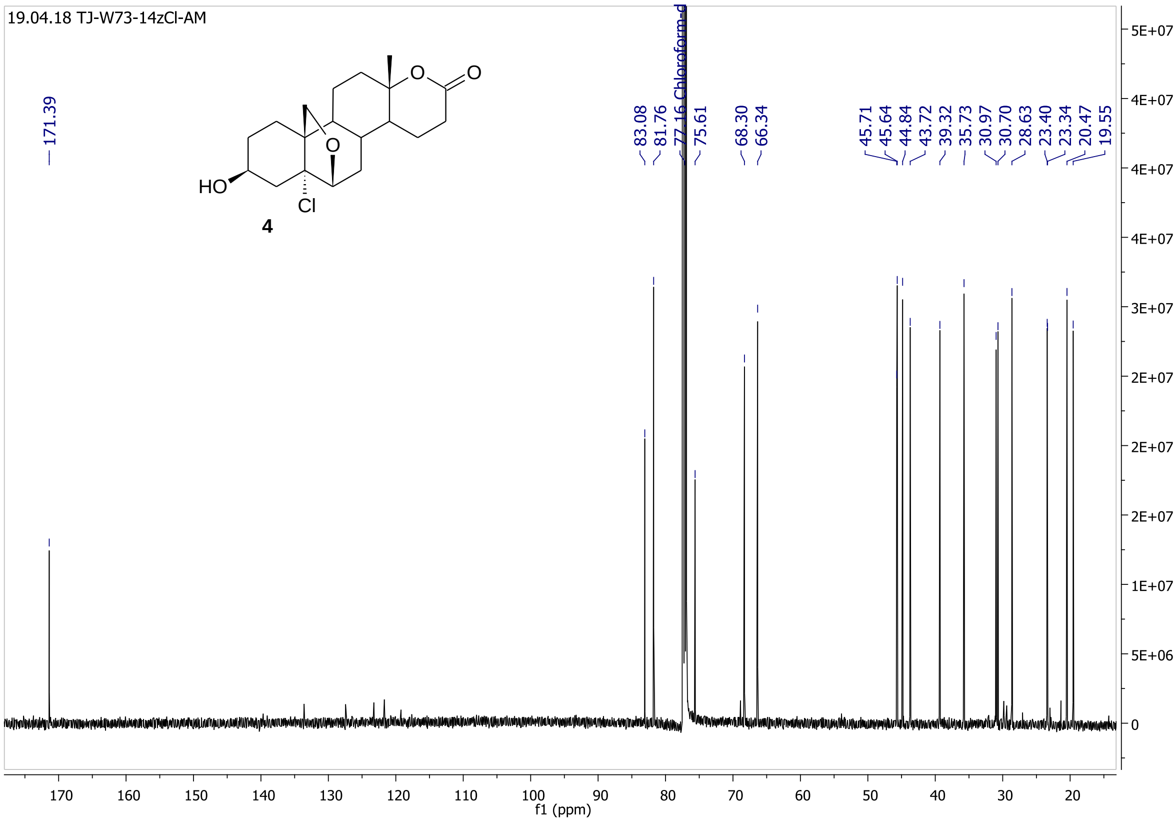

Fig.S31. HMQC spectral of 3β-hydroxy-5α-chloro-17a-oxa-D-homo-6,19-oxidoandrostan-17-one (**4**) ($CDCl_3$, 151 MHz)

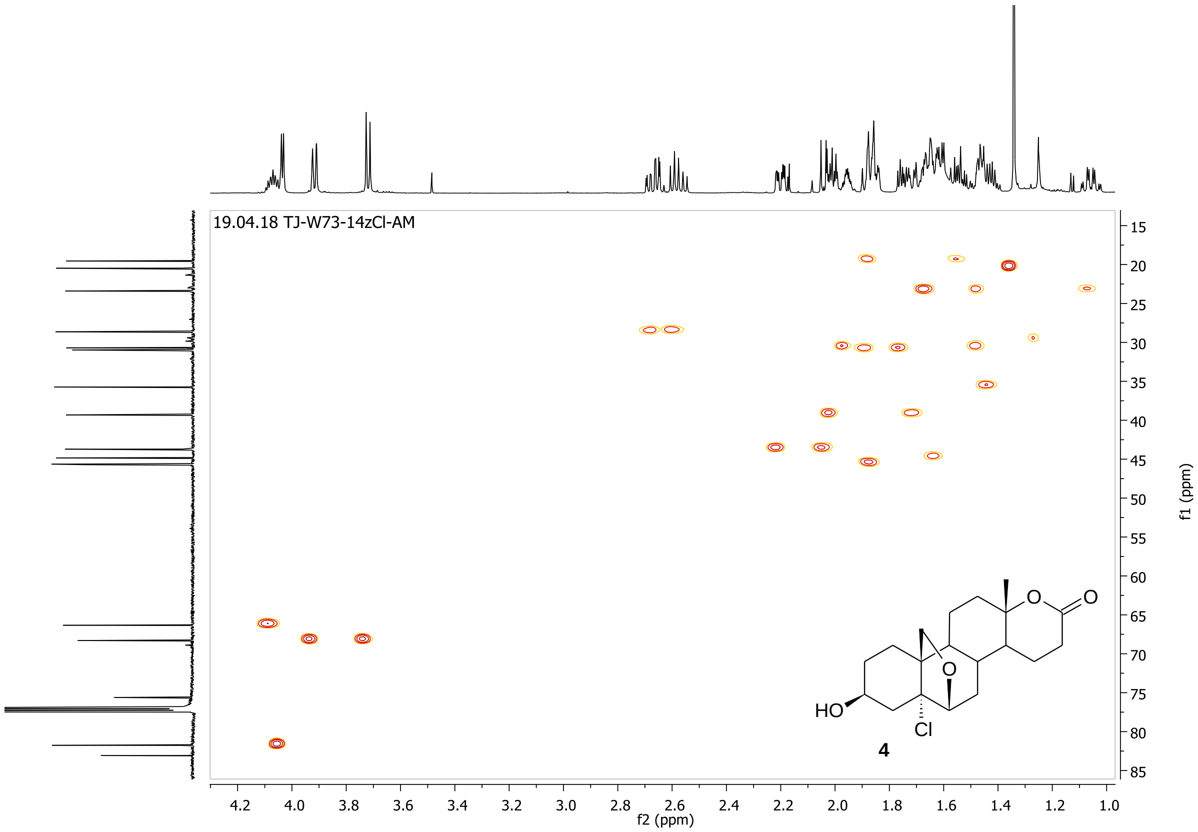

Fig.S32. HMBC spectral of 3β-hydroxy-5α-chloro-17a-oxa-D-homo-6,19-oxidoandrostan-17-one (**4**) ($CDCl_3$, 151 MHz)

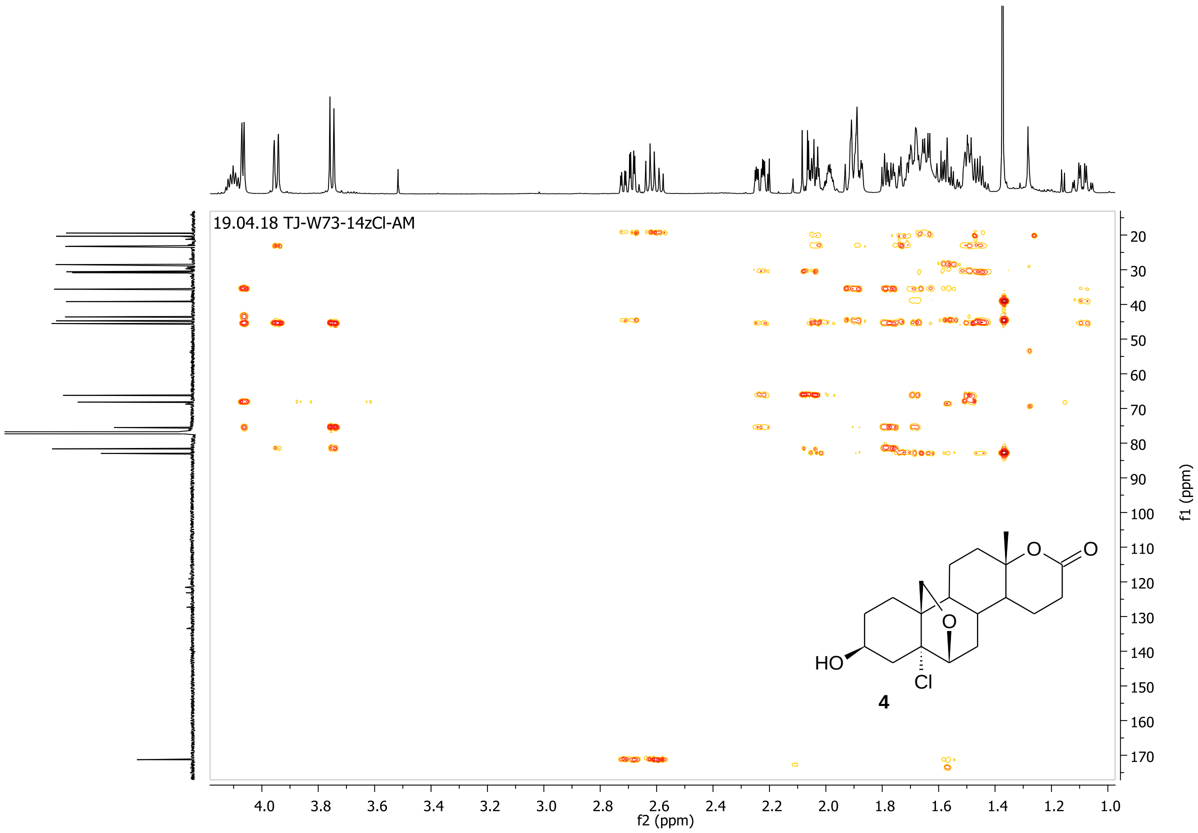

Fig.S33. COSY spectral of 3β-hydroxy-5α-chloro-17a-oxa-D-homo-6,19-oxidoandrostan-17-one (**4**) ($CDCl_3$, 151 MHz)

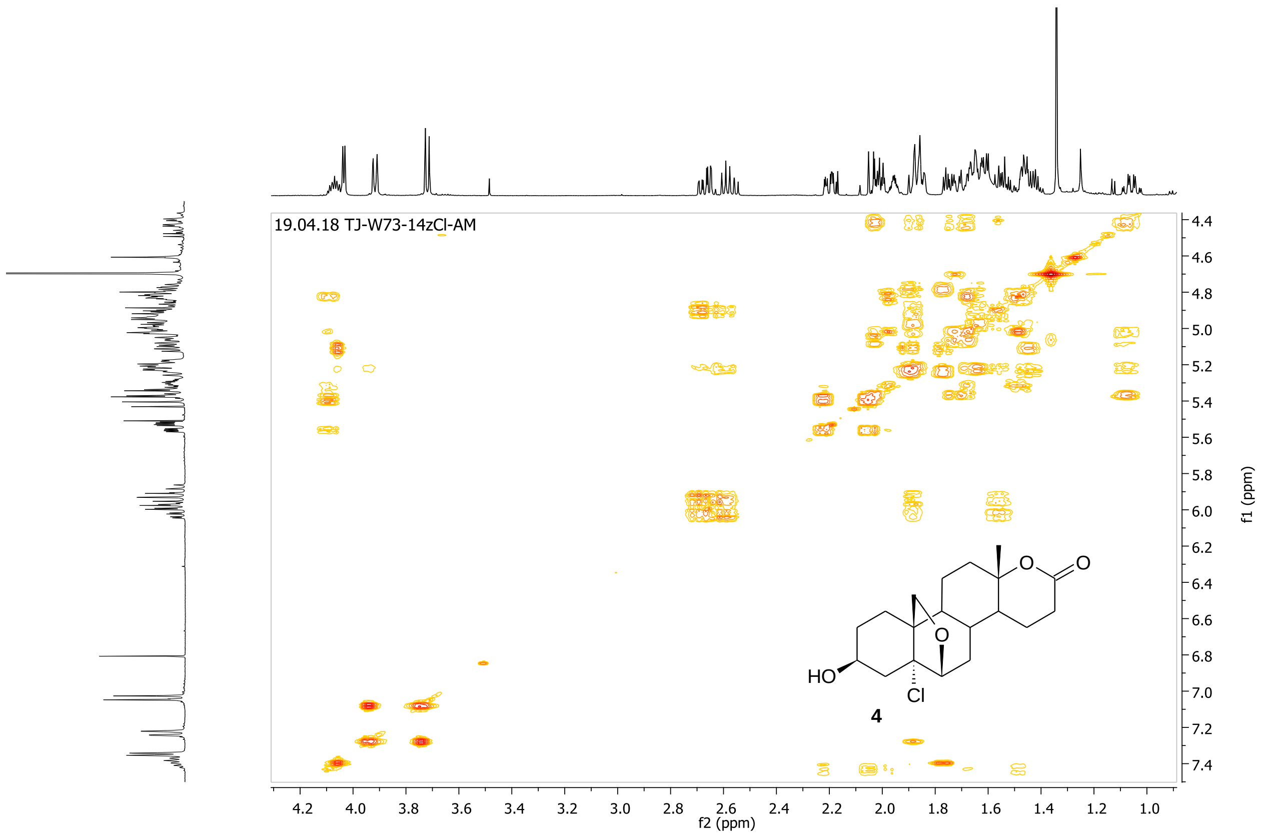

Fig.S34. GC-MS spectra of 3β,11α-dihydroxy-5α-chloro-17a-oxa-D-homo-6,19-oxidoandrostan-17-one (**5**)

Molecular Formula = $C_{19}H_{27}ClO_5$
Formula Weight = 370.86768

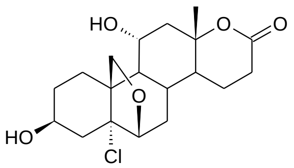

5

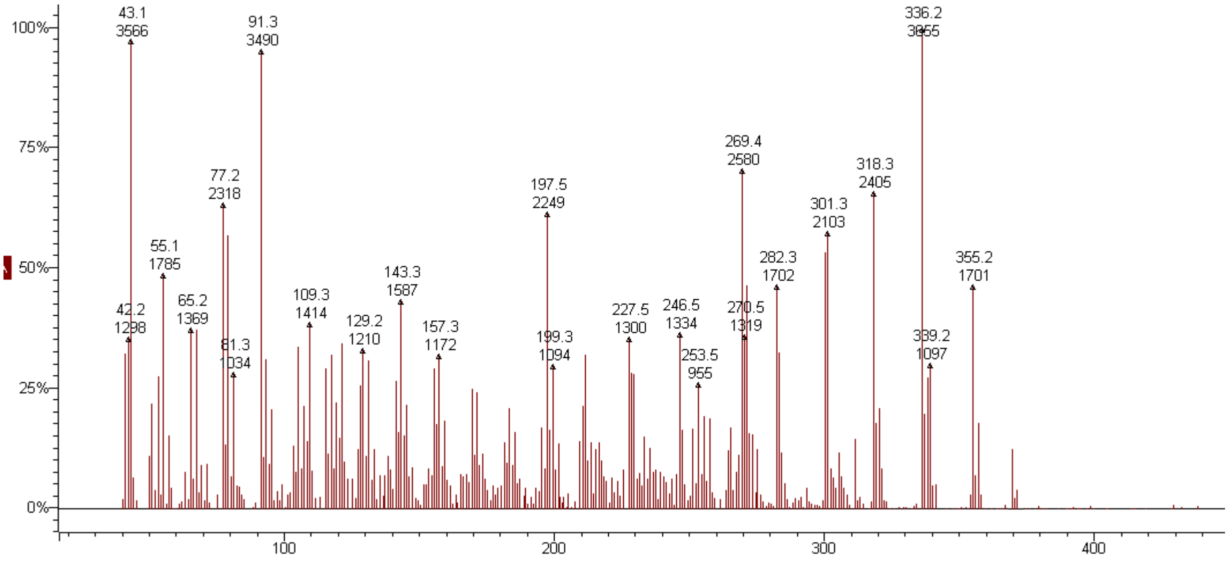

Fig.S35. ^{1}H NMR spectral of 3β,11α-dihydroxy-5α-chloro-17a-oxa-D-homo-6,19-oxidoandrostan-17-one (**5**) ($CDCl_3$, 600 MHz)

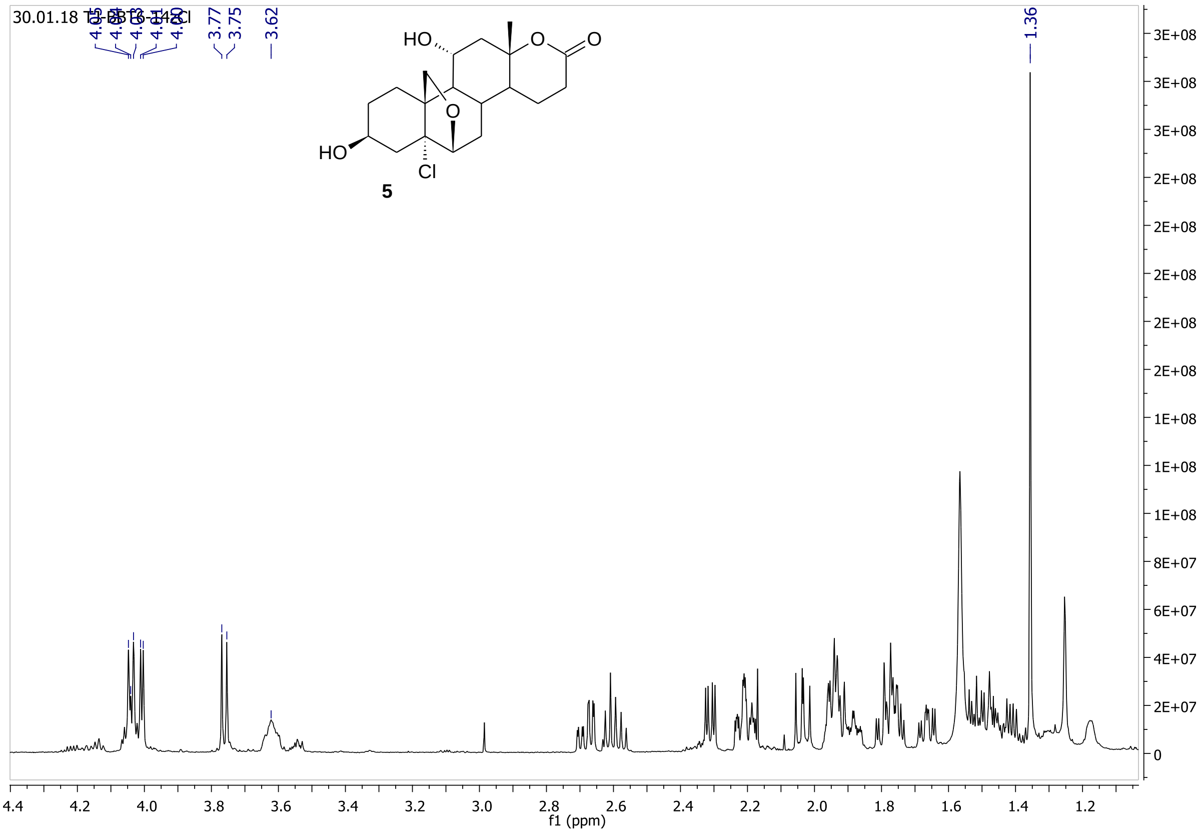

Fig.S36. ^{13}C NMR spectral of 3β,11α-dihydroxy-5α-chloro-17a-oxa-D-homo-6,19-oxidoandrostan-17-one (**5**) ($CDCl_3$, 151 MHz)

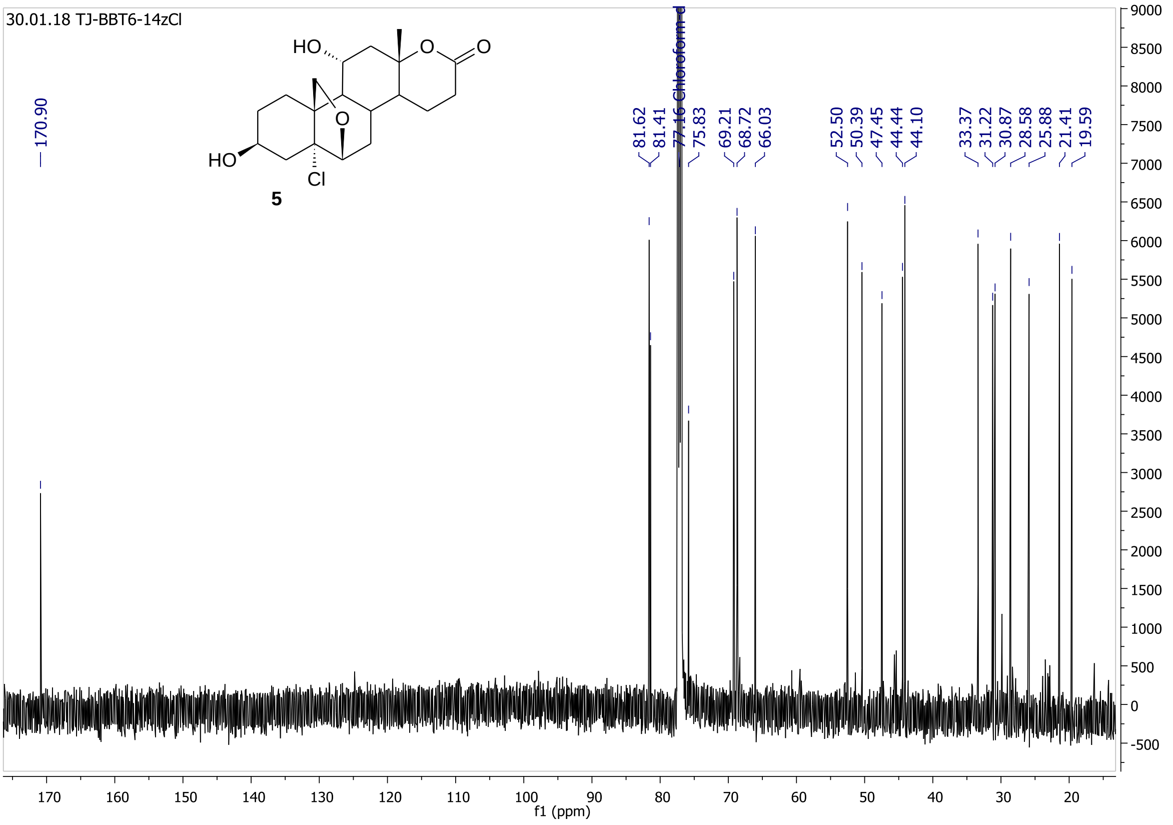

Fig.S37. HMQC spectral of 3β,11α-dihydroxy-5α-chloro-17a-oxa-D-homo-6,19-oxidoandrostan-17-one (**5**) (CDCl$_3$, 151 MHz)

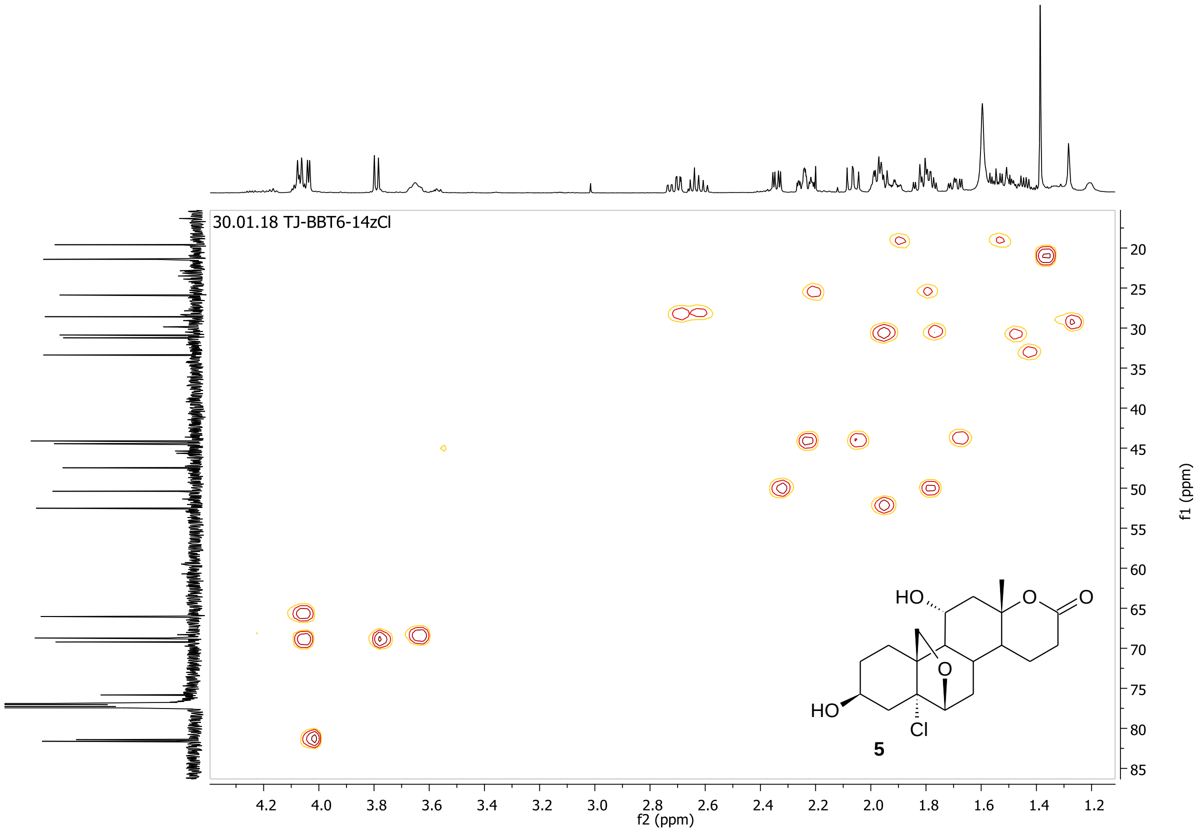

Fig.S38. HMBC spectral of 3β,11α-dihydroxy-5α-chloro-17a-oxa-D-homo-6,19-oxidoandrostan-17-one (**5**) (CDCl$_3$, 151 MHz)

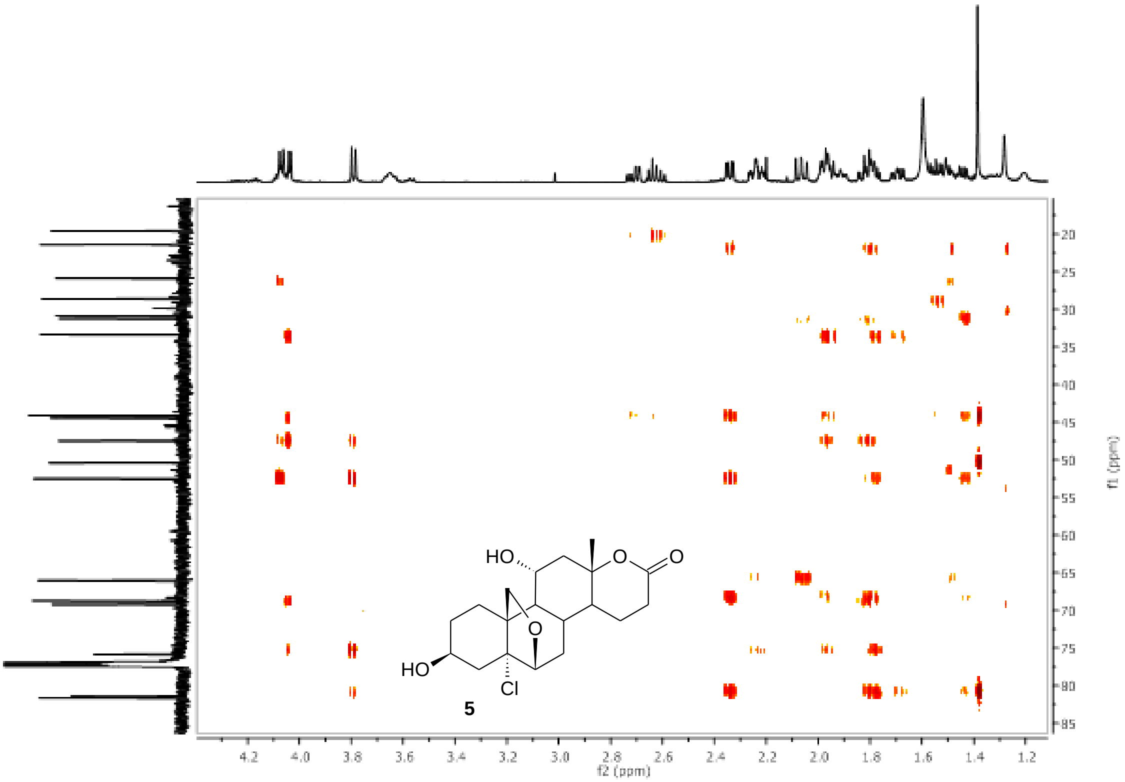

Fig.S39. COSY spectral of 3β,11α-dihydroxy-5α-chloro-17a-oxa-D-homo-6,19-oxidoandrostan-17-one (**5**) ($CDCl_3$, 151 MHz)

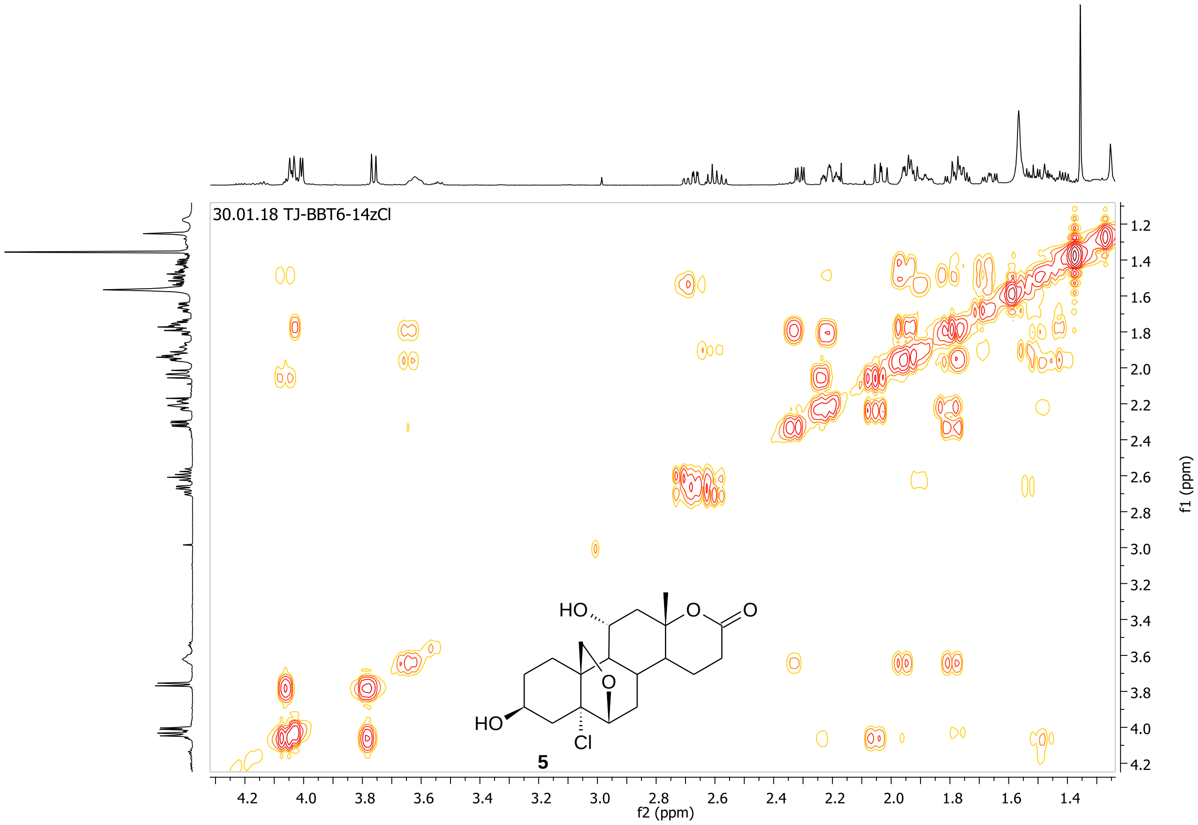

Fig.S40. GC-MS spectra of 3β,11α-dihydroxy-5α-chloro-6,19-oxidoandrostan-17-one (**6**)

Molecular Formula = $C_{19}H_{27}ClO_4$
Formula Weight = 354.86828

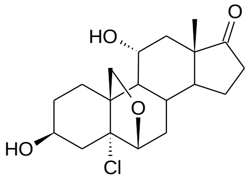

6

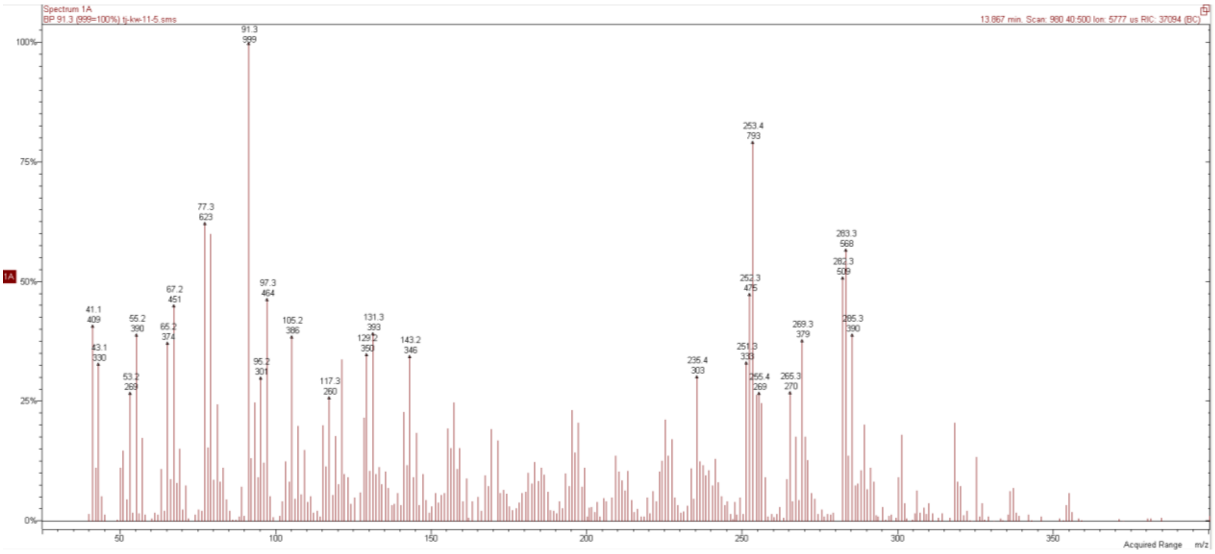

Fig.S41. ^{1}H NMR spectral of 3β,11α-dihydroxy-5α-chloro-6,19-oxidoandrostan-17-one (**6**) ($CDCl_3$, 600 MHz)

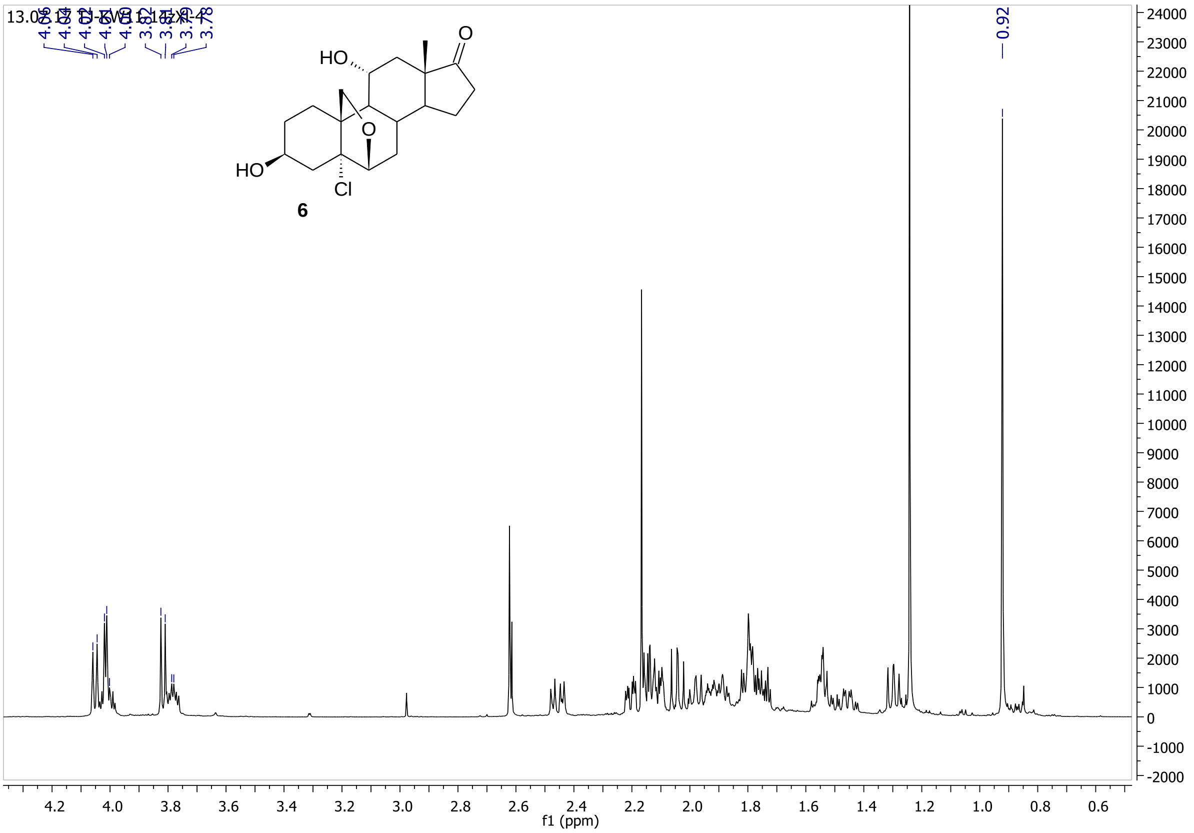

Fig.S42. ^{13}C NMR spectral of 3β,11α-dihydroxy-5α-chloro-6,19-oxidoandrostan-17-one (**6**) ($CDCl_3$, 151 MHz)

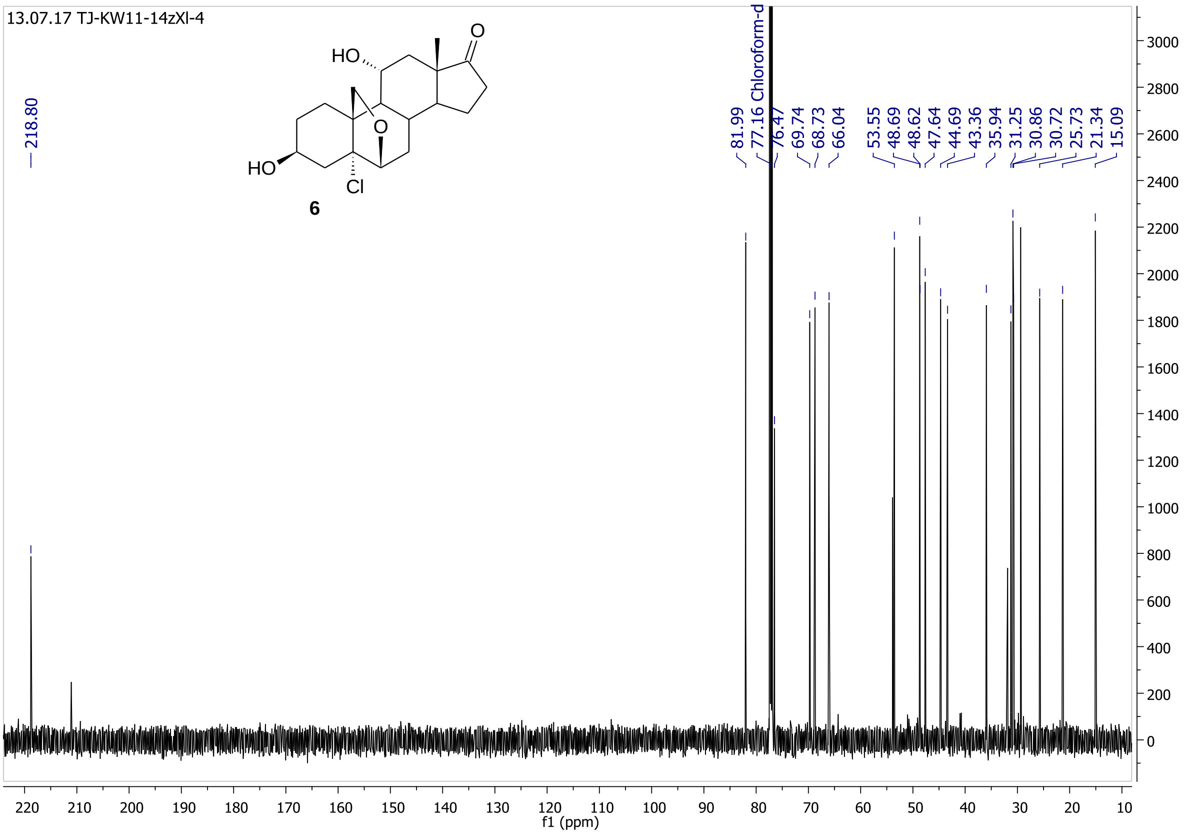

Fig.S43. HMQC spectral of 3β,11α-dihydroxy-5α-chloro-6,19-oxidoandrostan-17-one (**6**) ($CDCl_3$, 151 MHz)

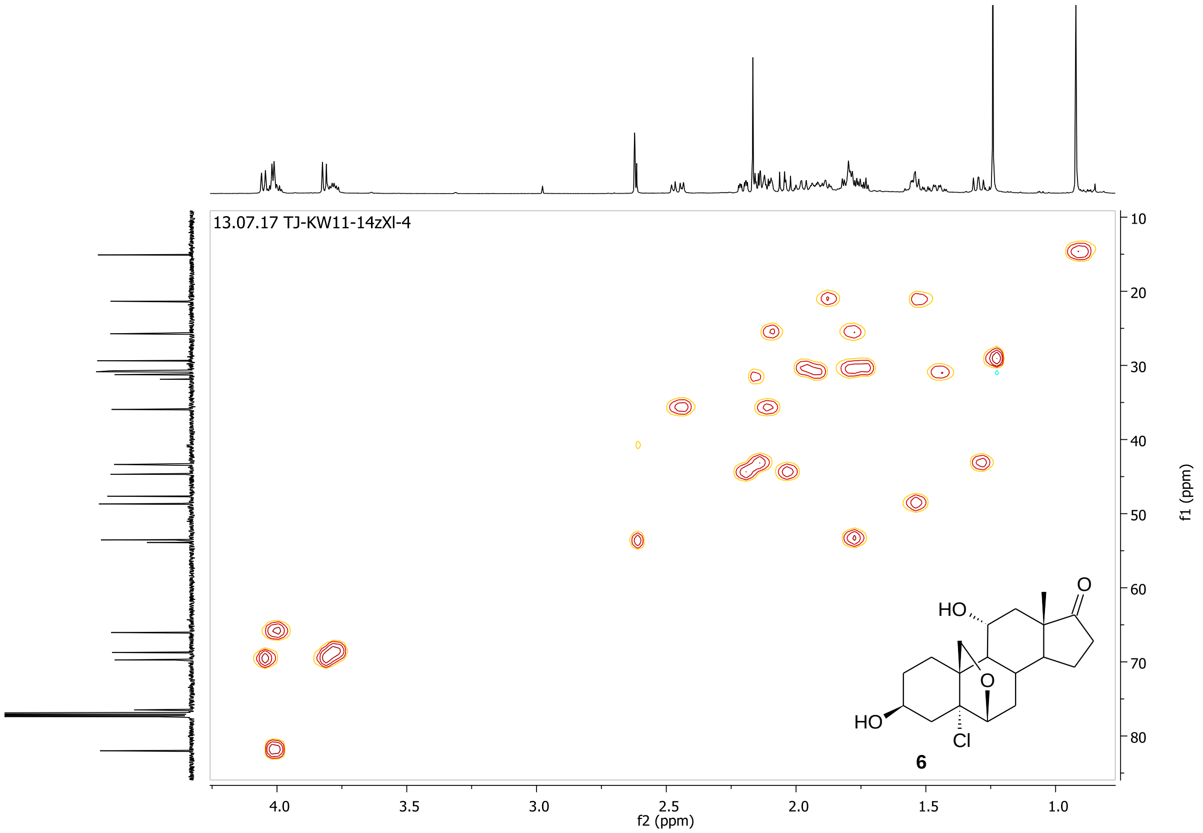

Fig.S44. HMBC spectral of 3β,11α-dihydroxy-5α-chloro-6,19-oxidoandrostan-17-one (**6**) ($CDCl_3$, 151 MHz)

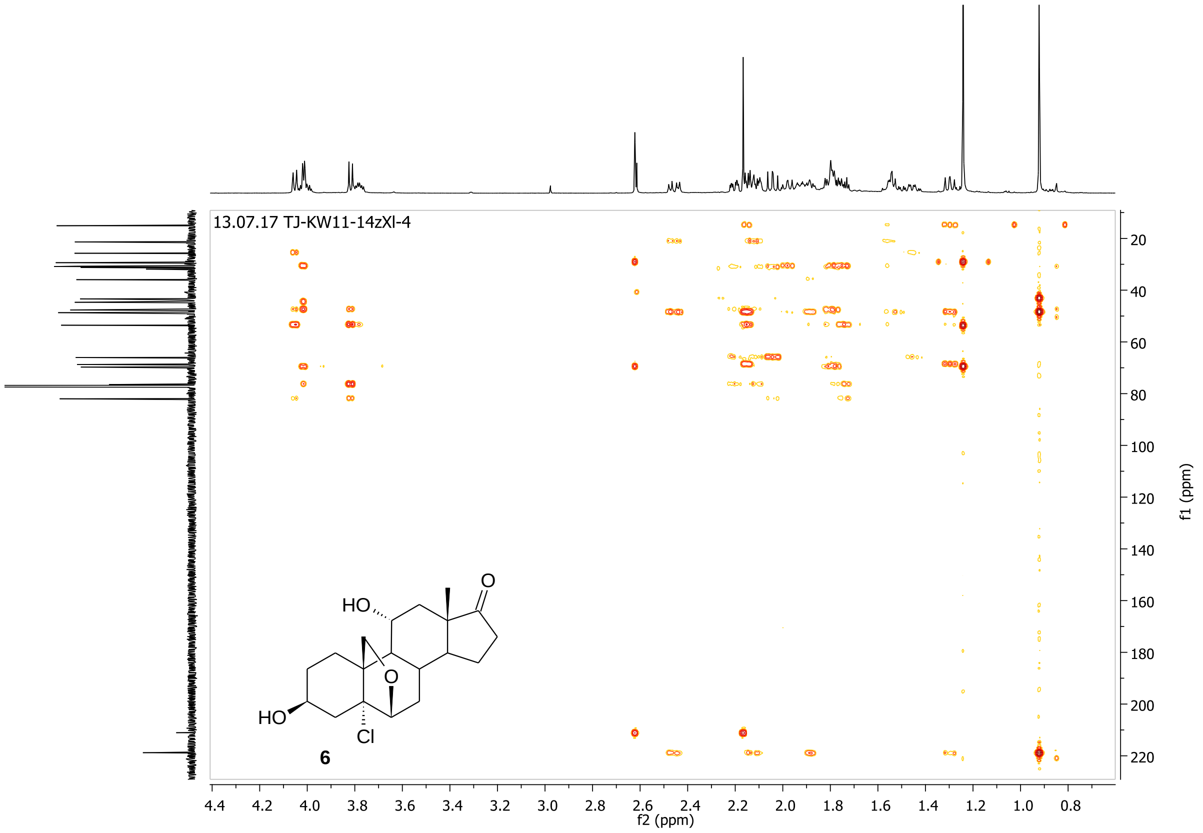

Fig.S45. COSY spectral of 3β,11α-dihydroxy-5α-chloro-6,19-oxidoandrostan-17-one (**6**) ($CDCl_3$, 151 MHz)

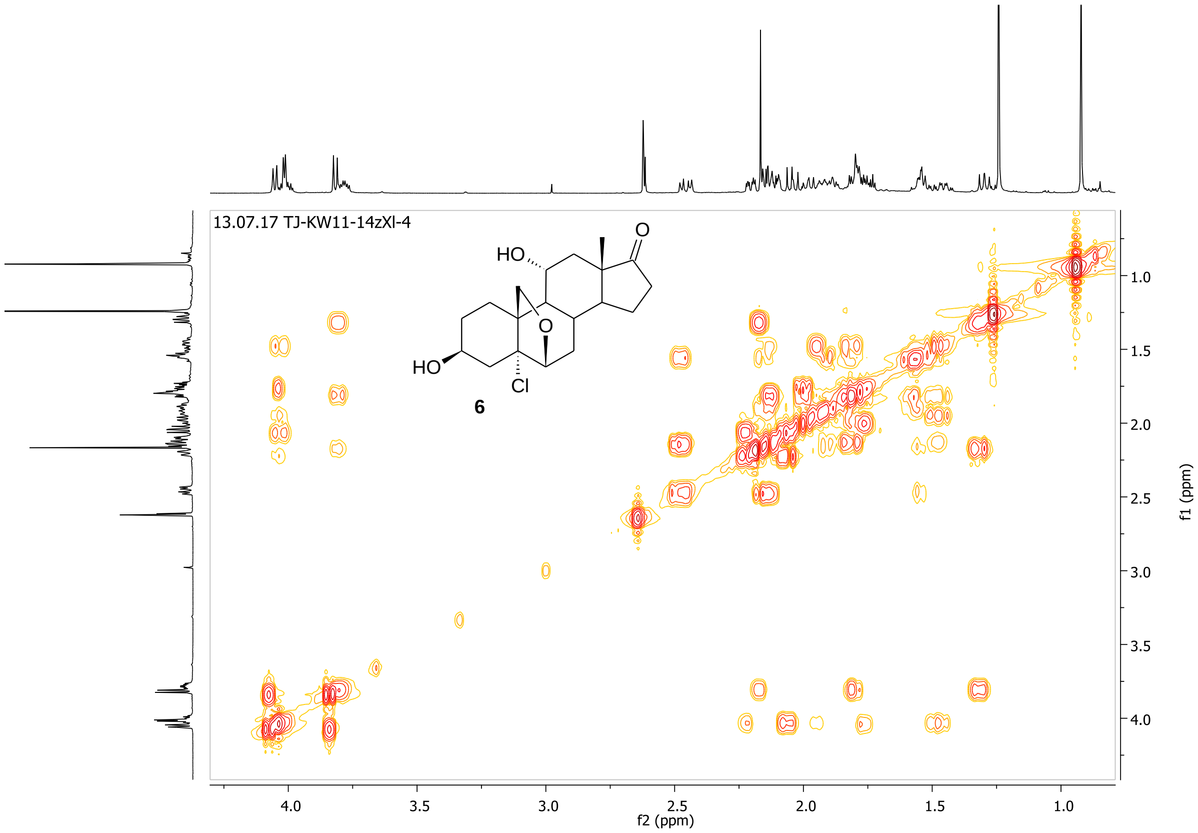

Fig.S46. GC-MS spectra of 3β,11α,19-trihydroxy-5α-chloro-6,19-oxidoandrostan-17-one (**7**)

Molecular Formula = $C_{19}H_{27}ClO_5$
Formula Weight = 370.86768

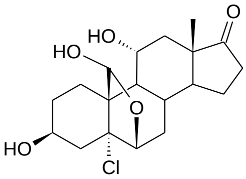

7

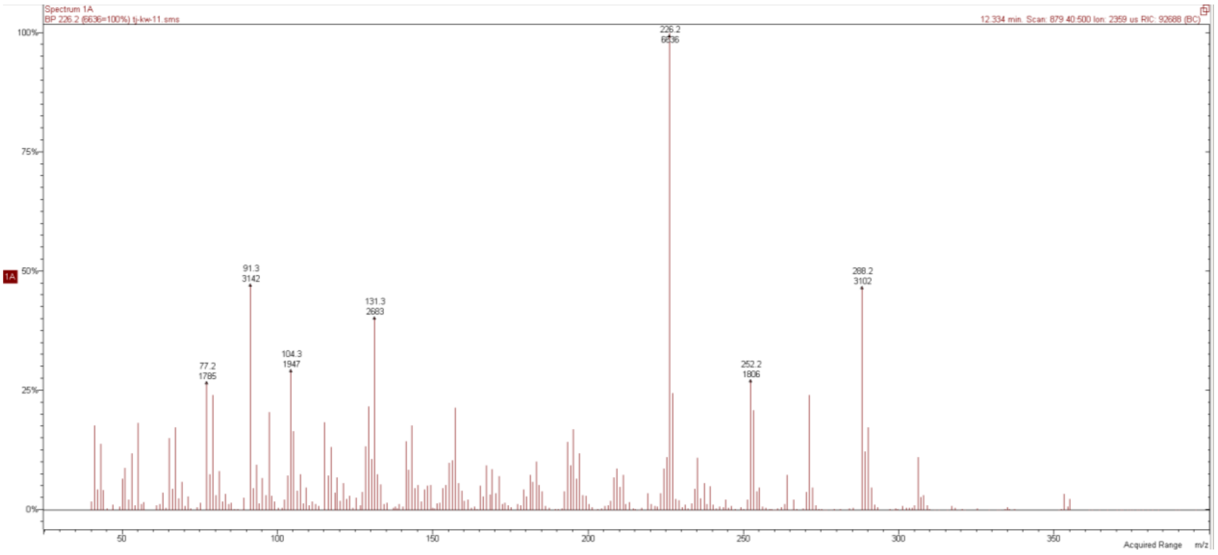

Fig.S47. ^{1}H NMR spectral of 3β,11α,19-trihydroxy-5α-chloro-6,19-oxidoandrostan-17-one (**7**) ($CDCl_3$, 600 MHz)

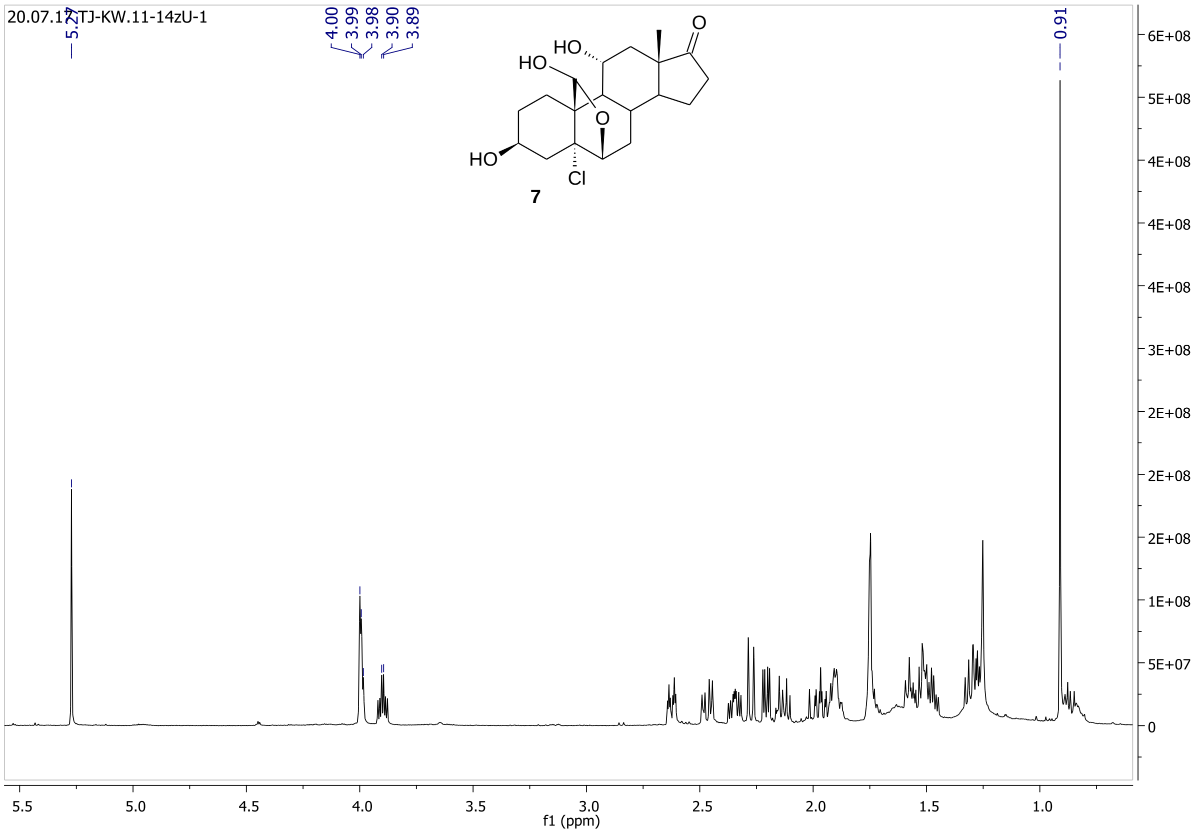

Fig.S48. ^{13}C NMR spectral of 3β,11α,19-trihydroxy-5α-chloro-6,19-oxidoandrostan-17-one (**7**) ($CDCl_3$, 151 MHz)

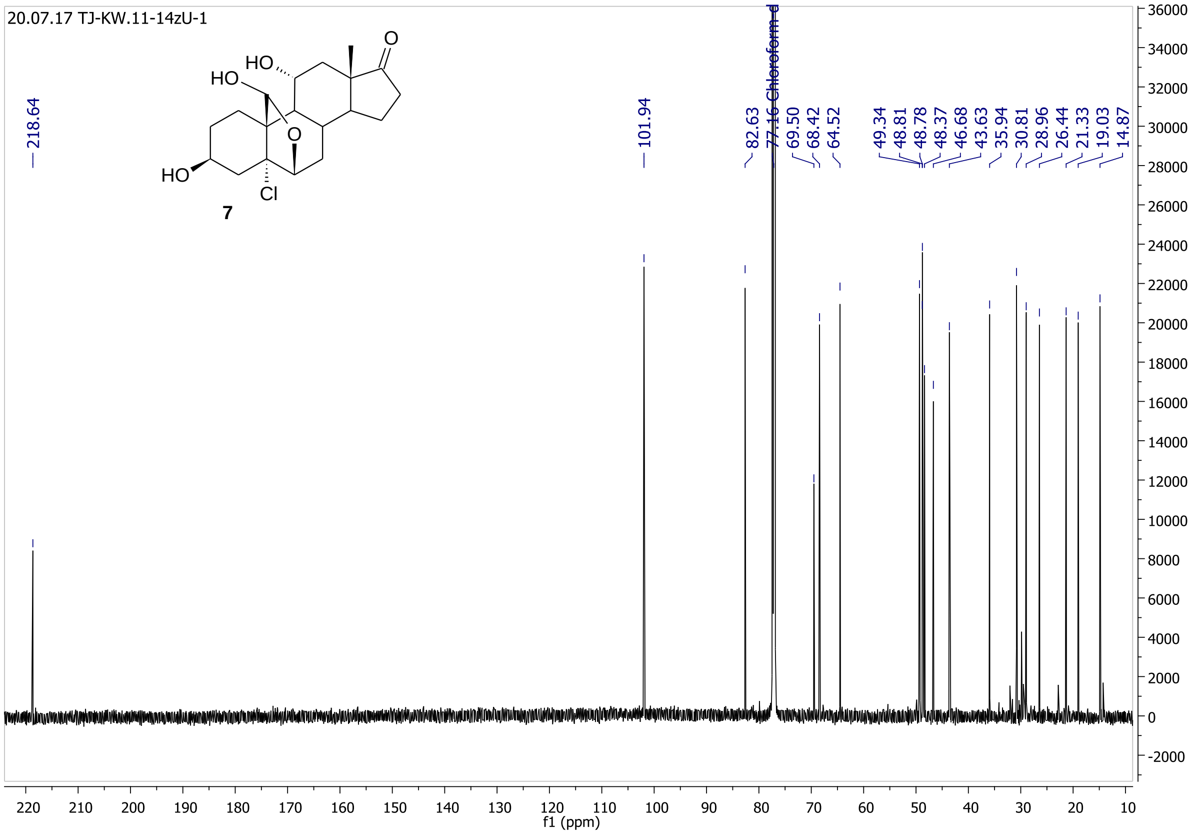

Fig.S49. HMQC spectral of 3β,11α,19-trihydroxy-5α-chloro-6,19-oxidoandrostan-17-one (**7**) ($CDCl_3$, 151 MHz)

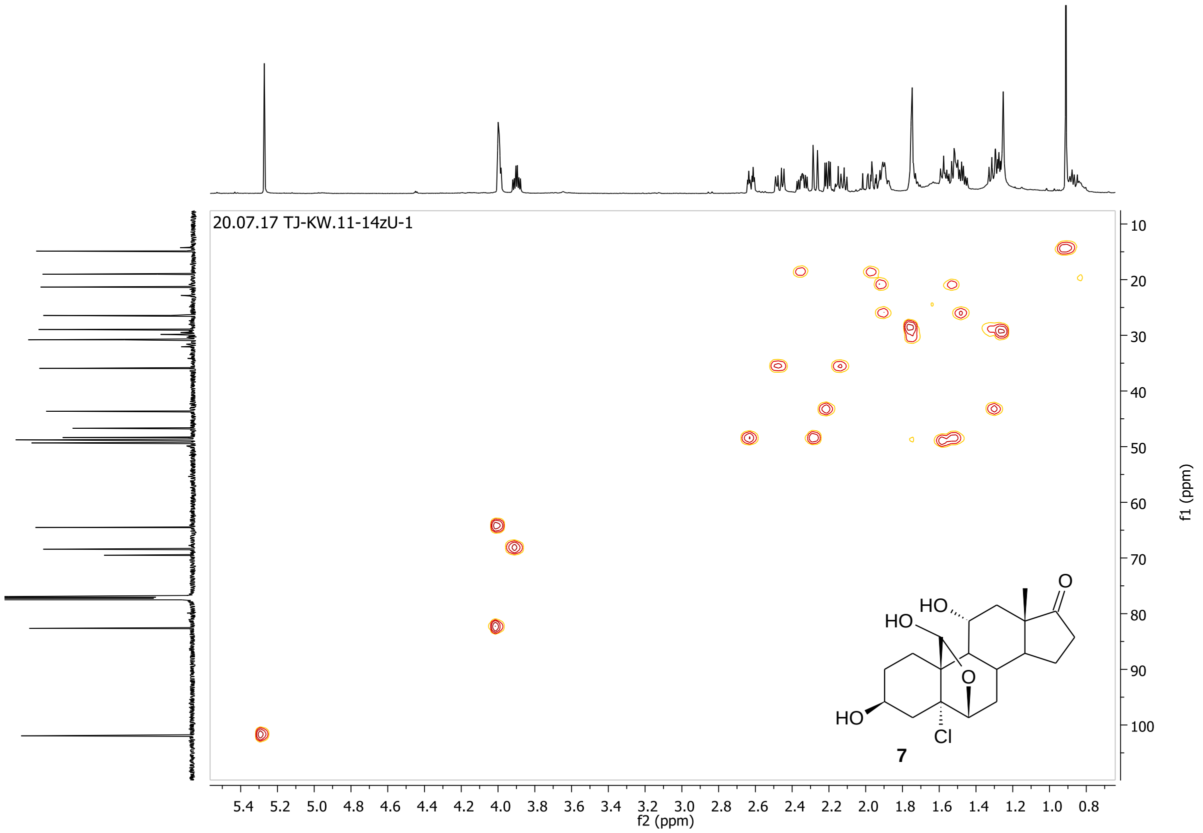

Fig.S50. HMBC spectral of 3β,11α,19-trihydroxy-5α-chloro-6,19-oxidoandrostan-17-one (**7**) ($CDCl_3$, 151 MHz)

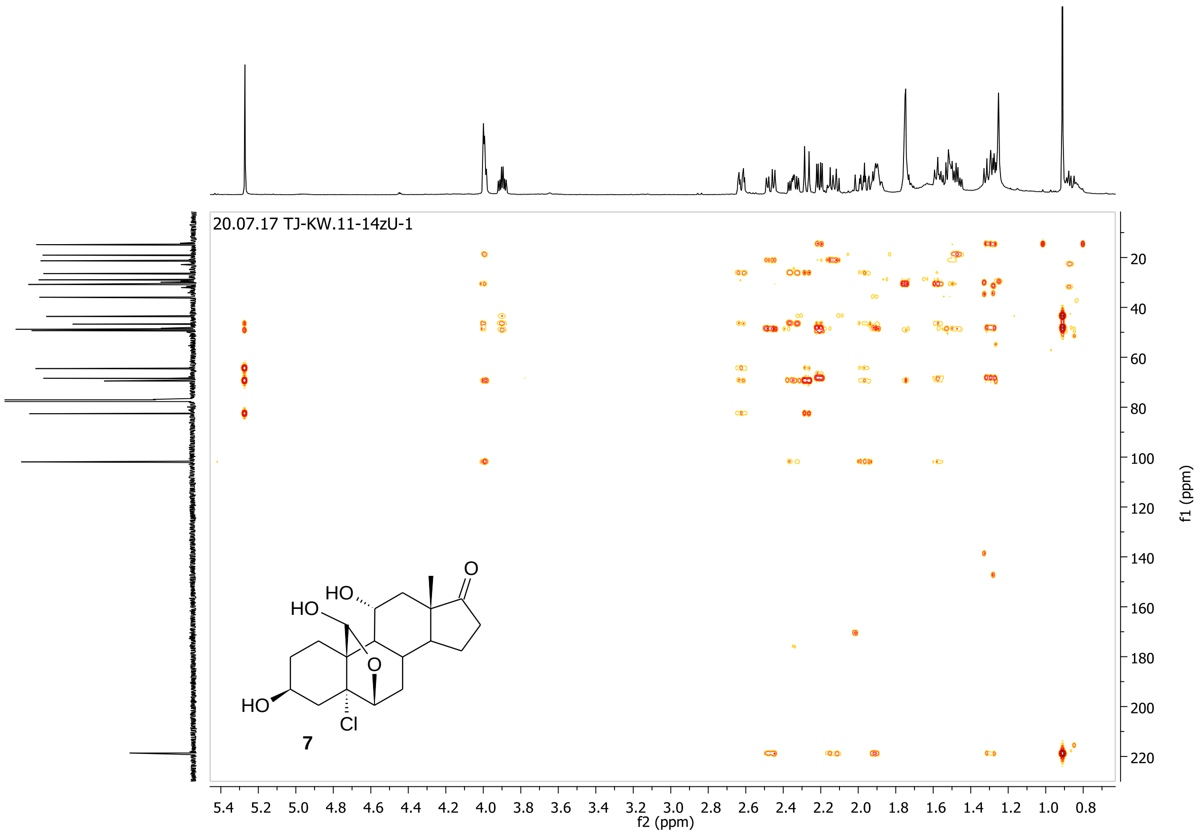

Fig.S51. COSY spectral of 3β,11α,19-trihydroxy-5α-chloro-6,19-oxidoandrostan-17-one (**7**) ($CDCl_3$, 151 MHz)

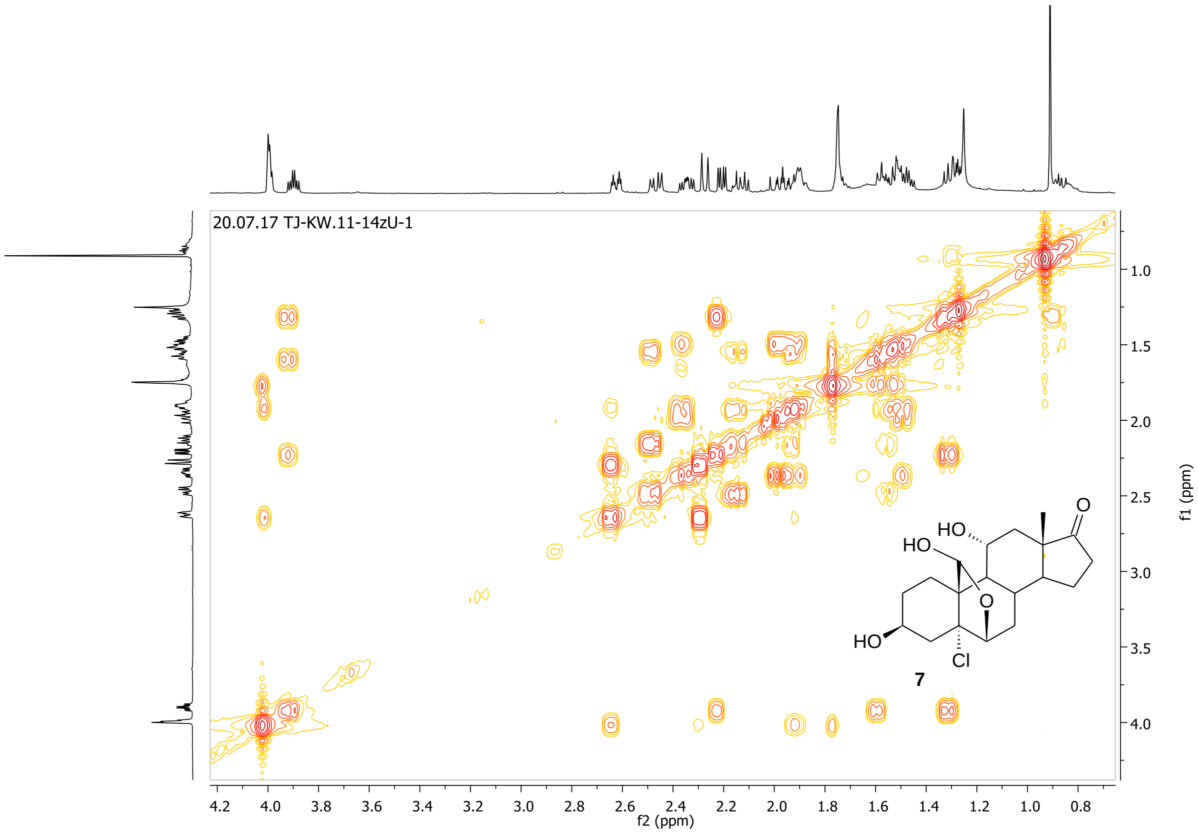

Fig.S52. GC-MS spectra of 3β,11α-dihydroxy-5α-chloro-6,19-oxidoandrostan-17,19-dione (**8**)

Molecular Formula = $C_{19}H_{25}ClO_5$
Formula Weight = 368.8518

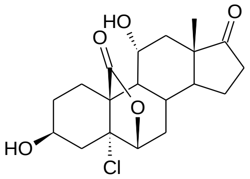

8

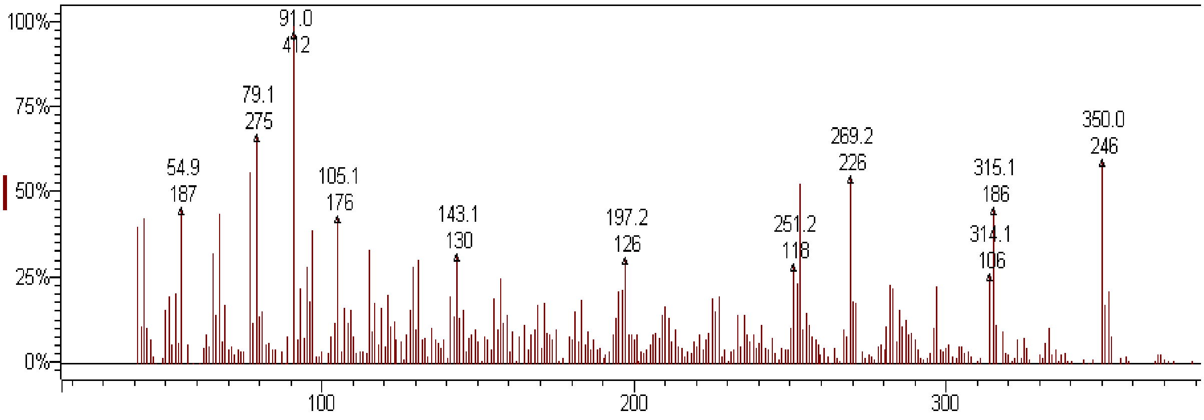

Fig.S53. ^{1}H NMR spectral of 3β,11α-dihydroxy-5α-chloro-6,19-oxidoandrostan-17-one (**6**) and 3β,11α-dihydroxy-5α-chloro-6,19-oxidoandrostan-17,19-dione (**8**) (DMSO-d_6, 600 MHz)

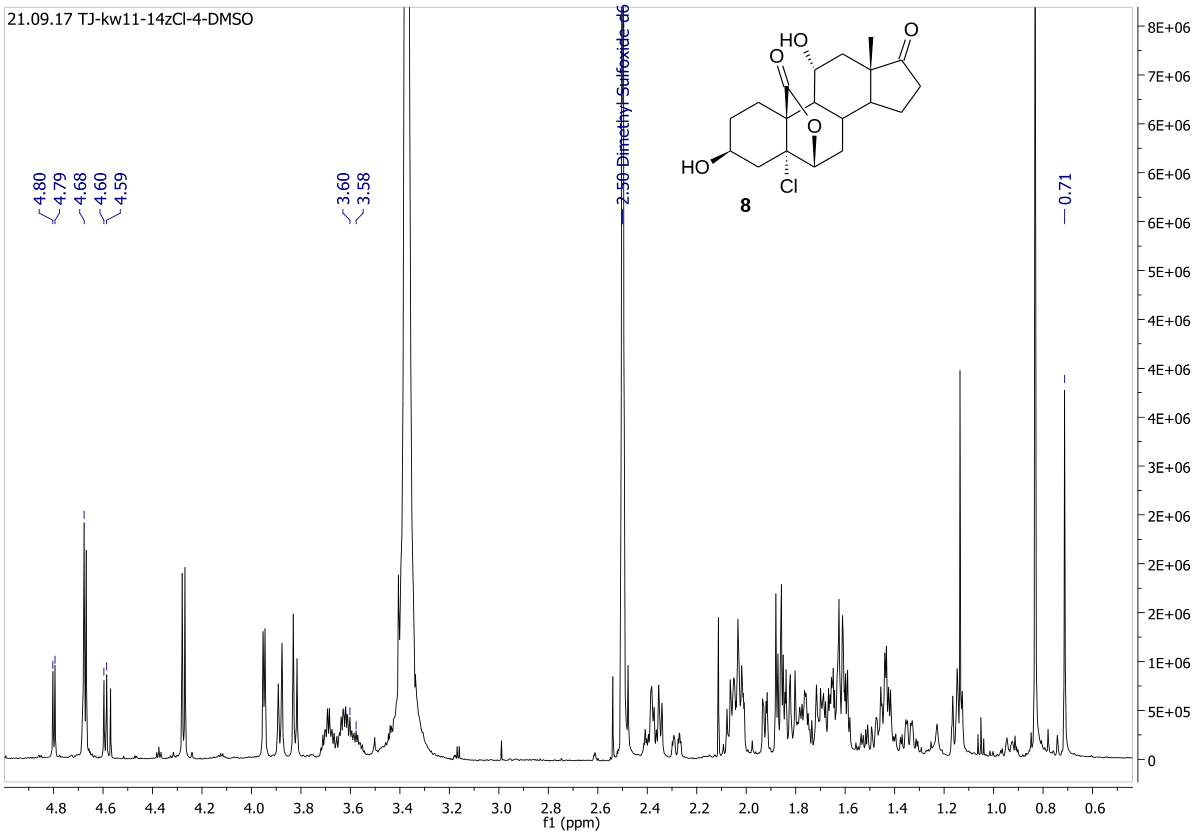

Fig.S54. ^{13}C NMR spectral of 3β,11α-dihydroxy-5α-chloro-6,19-oxidoandrostan-17-one (**6**) and 3β,11α-dihydroxy-5α-chloro-6,19-oxidoandrostan-17,19-dione (**8**) (DMSO-d_6, 151 MHz). Signals from carbons of compound **8** were assigned.

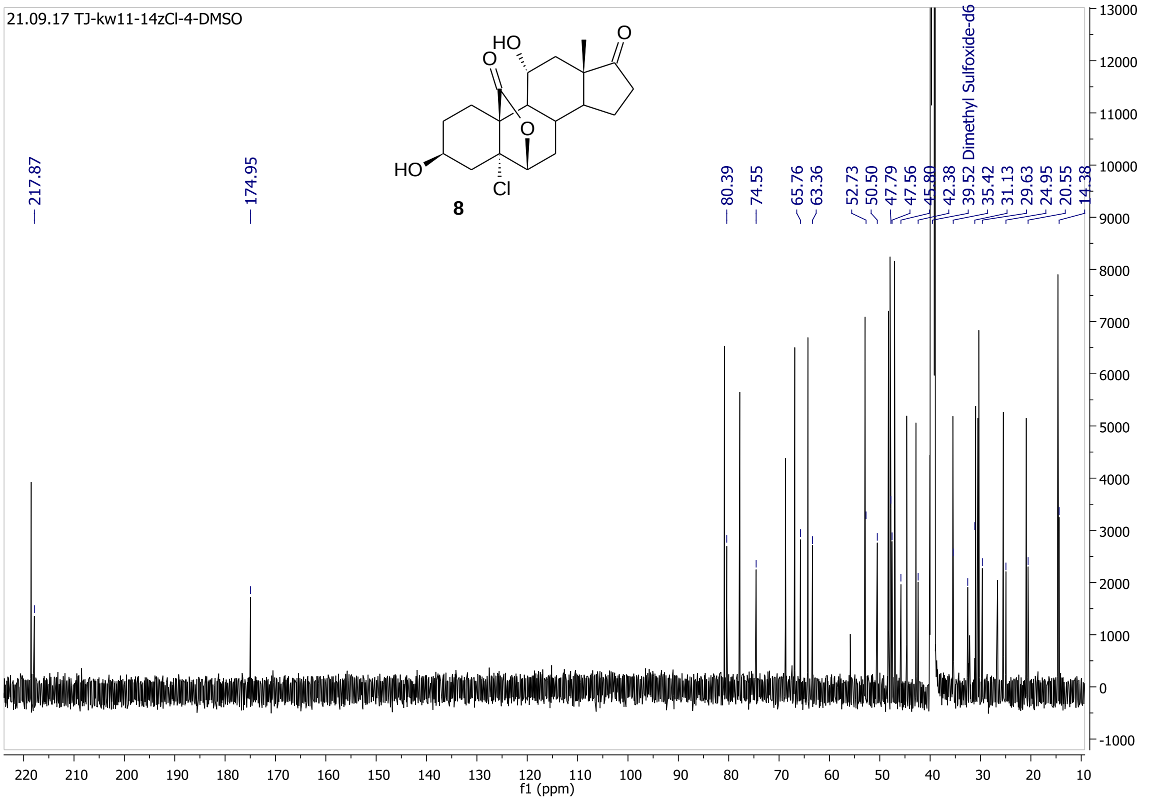

Fig.S55. ^{13}C NMR spectral of 3β,11α-dihydroxy-5α-chloro-6,19-oxidoandrostan-17-one (**6**) and 3β,11α-dihydroxy-5α-chloro-6,19-oxidoandrostan-17,19-dione (**8**) (DMSO-d_6, 151 MHz). Signals from carbons of compound **6** were assigned.

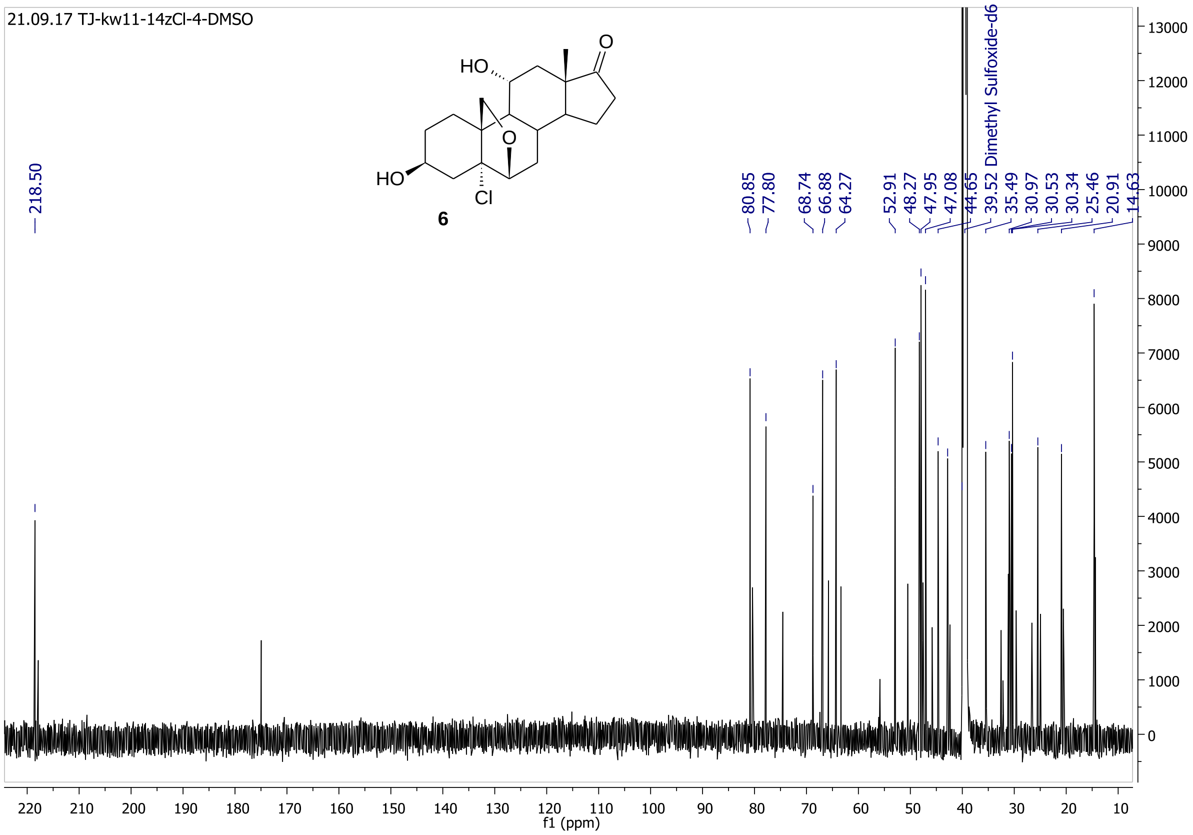

Fig.S56. HMQC spectral of 3β,11α-dihydroxy-5α-chloro-6,19-oxidoandrostan-17-one (**6**) and 3β,11α-dihydroxy-5α-chloro-6,19-oxidoandrostan-17,19-dione (**8**) (DMSO-d_6, 151 MHz)

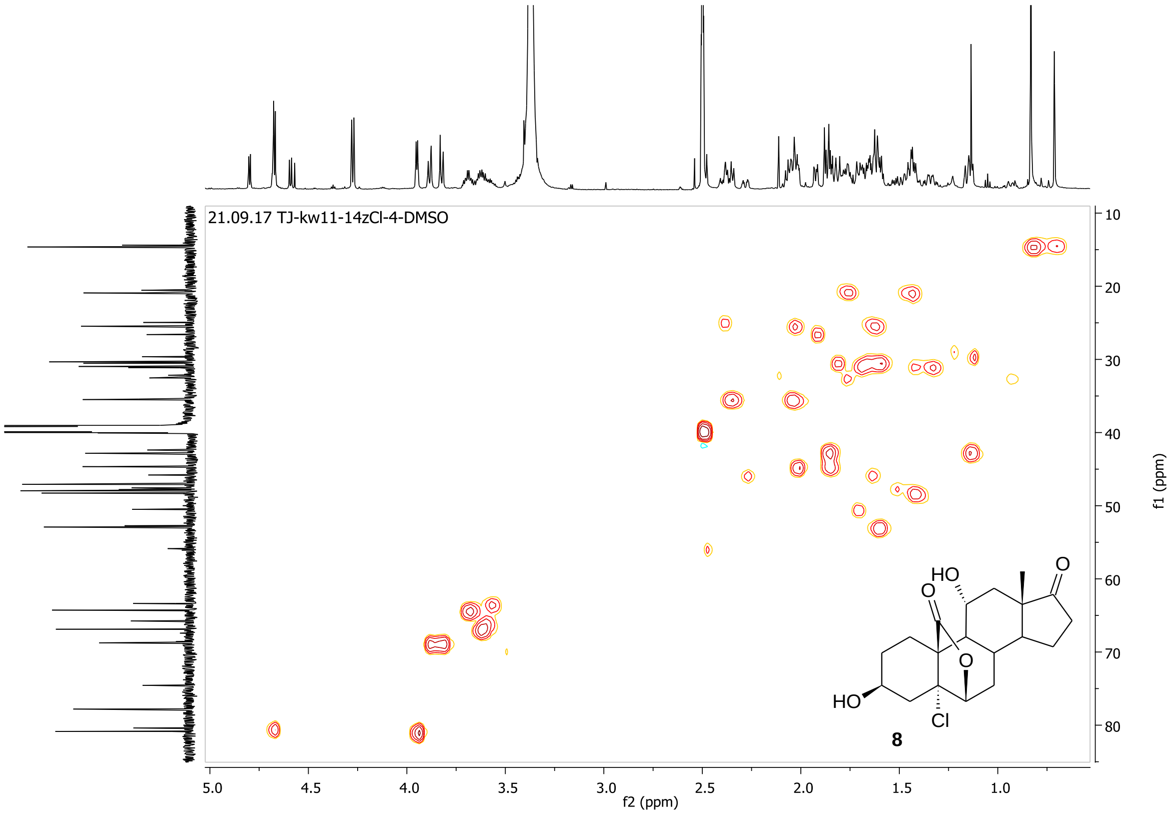

Fig.S57. COSY spectral of 3β,11α-dihydroxy-5α-chloro-6,19-oxidoandrostan-17-one (**6**) and 3β,11α-dihydroxy-5α-chloro-6,19-oxidoandrostan-17,19-dione (**8**) (DMSO-d_6, 151 MHz)

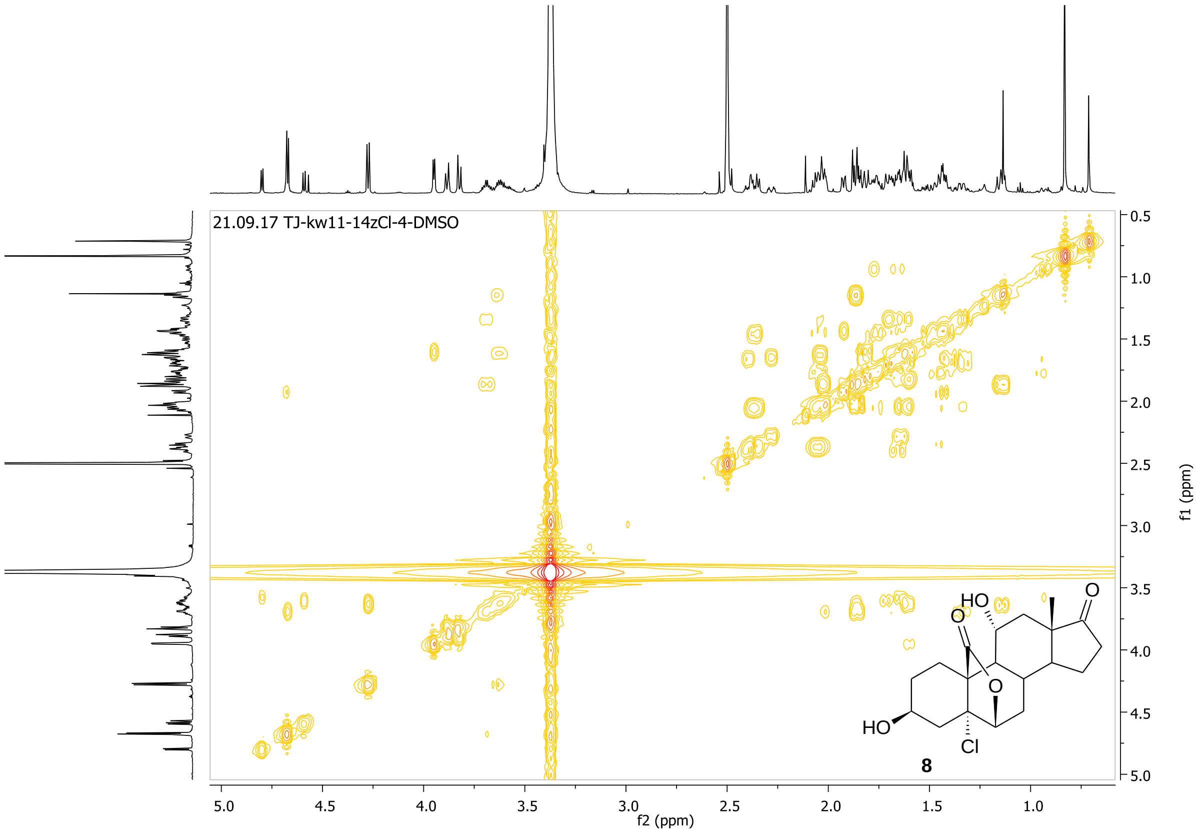

Supplement: Supplementary file 1 — Additional file 1. Spectral data of the substrate and all obtained products. [file 12934_2020_1303_MOESM1_ESM.pdf]
